# A mononuclear nonheme iron complex with higher affinity for O_2_ than CO via hydrogen bonding

**DOI:** 10.1038/s41467-026-72599-y

**Published:** 2026-05-05

**Authors:** Matthias Jux, Stefan Mebs, Michael Haumann, Sagie Katz, Ricardo Garcia-Serres, Peter Hildebrandt, Yong Wang, Wonwoo Nam, Kallol Ray

**Affiliations:** 1 Department of Chemistry, Humboldt-Universität zu Berlin, Berlin, Germany; 2 Department of Physics, Freie Universität Berlin, Berlin, Germany; 3 Institute of Chemistry, Technische Universität Berlin, Berlin, Germany; 4 Laboratoire Chimie et Biologie des Métaux, Grenoble, France; 5 Institute of Drug Discovery Technology, Ningbo University, Ningbo, China; 6 Department of Chemistry and Nano Science, Ewha Womans University, Seoul, South Korea

**Keywords:** Ligands, Catalyst synthesis, Chemical bonding

## Abstract

Dioxygen activation at iron centers is central to many biological and synthetic oxidation processes. In proteins, the reactivity and stability of iron–dioxygen intermediates are often controlled by secondary-sphere interactions such as hydrogen bonding. For example, hemoglobin stabilizes a Fe−O_2_ adduct through distal hydrogen bonding, while hemerythrin employs hydrogen bonding to stabilize reduced oxygen species within a diiron active site, enabling reversible O_2_ binding. Here we show that a mononuclear nonheme iron complex, [Fe^II^(DIG_3_tren)]^2+^ (DIG_3_tren = tris(N’,N”-diisopropylguanidinyl-2-ethyl)amine), reversibly reduces O_2_ by two electrons to generate an iron(IV)-peroxido species. Strong hydrogen bonds from N − H groups of the ligand stabilize the O_2_^2−^ ligand, while the electron-rich guanidine donors promote the unusual Fe^II^-mediated two-electron reduction of O_2_. As a result, the complex exhibits higher affinity for O_2_ than for CO due to preferential hydrogen-bond stabilization of the peroxido intermediate. These results demonstrate how secondary-sphere design can control both O_2_ activation and ligand selectivity at iron centers.

## Abstract (web summary)

A challenge in designing a synthetic system that can reversibly bind O_2_ is to prevent irreversible oxidation to oxo-bridged dimers. Here, the authors report a mononuclear nonheme iron(II) complex where the second coordination sphere is engineered to ensure a reversible two-electron reduction of dioxygen to form an iron(IV)-peroxido species through the inclusion of hydrogen bonding substituents.

## Introduction

**Fig. 1:**
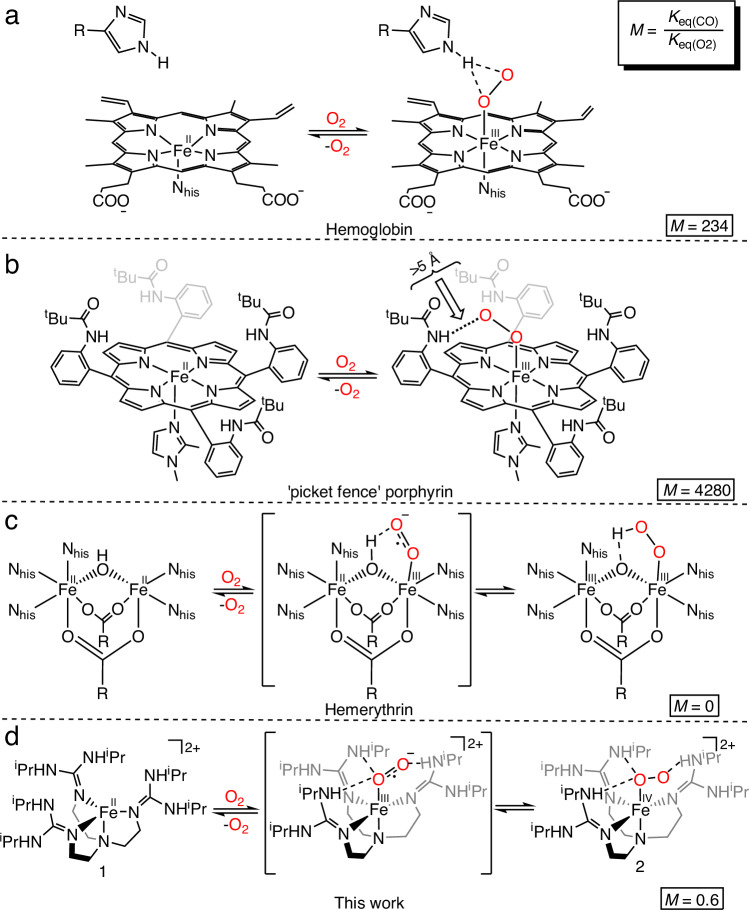
Oxygen binding at iron centers. Oxygen binding at the iron center in **a** hemoglobin, **b** picket fence porphyrin, **c** hemerythrin and **d** [Fe(DIG_3_tren)](OTf)_2_. Dashed lines represent H-bonding.

The binding of dioxygen at a ferrous site in proteins is an indispensable step in a variety of physiologically important reactions involved in dioxygen metabolism^1–7^. A common mechanistic hypothesis for dioxygen activation has been established based on intense research in this field over the past several decades^8–33^. The one-electron reduction of dioxygen to form an iron(III)-superoxido species^34–39^, which is energetically endergonic, is invariably the first step proposed during dioxygen activation. It is followed by fast proton-coupled electron-transfer steps that drive O–O bond cleavage to afford high-valent iron-oxido species, which are typically considered as the reactive intermediates responsible for mono- or dioxygenation of substrates. For reversible binding of dioxygen, in contrast, it is warranted that the high-energy iron(III)-superoxido intermediate is stabilized and prevented from further reduction. In hemoglobin (Hb) and myoglobin (Mb)^40,41^, for example, the protein structure surrounding the heme active site may play a significant role in enhancing superoxido stability. X-ray crystallographic studies suggest a role for H-bonding between the “distal histidine” site and the bound superoxido anion (Fig. 1a)^42^. Notably, the distal histidine in Hb/Mb is ideally situated to H-bond with bound oxygen, but out of place for optimal interaction with a bound CO, which is believed to be crucial for increasing its affinity for dioxygen compared to carbon monoxide.

In the absence of any protective protein scaffold, one of the most difficult aspects of creating a synthetic system capable of reversibly binding O_2_ is preventing irreversible oxidation to oxo-bridged dimers, which act as thermodynamic sink in these reactions^43,44^. Collman and coworkers^19,45,46^ addressed this challenge synthetically by constructing a protective fence around one face of the corresponding iron-porphyrin complex using an atropisomer of an ortho-substituted meso-tetraphenylporphyrin (Fig. 1b). Additionally, by employing a sterically encumbered imidazole, this fence limited the coordination of any axial ligand to the unhindered side of the iron center, without hindering the binding of small molecules, such as dioxygen, within the cavity of the hindered face. Furthermore, the coordinated O₂ unit was protected from further reacting with a second iron center, thereby preventing the bimolecular decay pathway. These properties made the iron-dioxygen adduct stable enough to be characterized by single-crystal X-ray crystallography, which provided structural insight into the coordination mode of dioxygen in Hb and Mb^47–50^. However, although the amide linkages that form the fence could potentially act as H-bond donors to the Fe(III)-superoxido unit in a similar manner as the histidine residue in Hb/Mb, these groups are too far removed from the Fe(III)-superoxido unit (a distance of over 5 Å; Fig. 1b) to form intramolecular H-bonds. Accordingly, it is found to be much poorer at binding O_2_, relative to Hb.

In addition to heme-based respiratory proteins, the necessity for intramolecular H-bonds in enabling reversible O_2_ binding is also proposed in hemerythrin (Hr), which involves a nonheme diiron (Fe^II^-Fe^II^) active site^51^. Here, a proton-coupled electron-transfer process (Fig. 1c), which effects a superoxido-to-hydroperoxido conversion with the distal Fe^II^ center being the electron donor, has been put forward to rationalize reversible O_2_ binding in hemerythrin. Furthermore, the hydroperoxide species is proposed to be stabilized against irreversible O–O bond cleavage through a H-bond with the resulting µ-oxo bridge (Fig. 1c)^2,51–54^. Incorporation of these non-covalent interactions has therefore been a goal for synthetic chemists who desire to replicate the reactivity of metalloproteins. However, this proved to be challenging, as in addition to incorporating H-bond donors/acceptors, it also required their proper positioning that would allow their interaction with small molecules coordinated to the metal center(s). Although Collman’s porphyrin picket fence was unable to participate in H-bonding with the coordinated O_2_ unit^45,46^, this system established the feasibility of positioning functional groups within a molecule to facilitate H-bonding through incorporation into a rigid scaffold. Other groups have also extended this concept in nonheme ligand systems in the design of rigid ligand frameworks that establish local C_3_ symmetry around a metal center^34,36,55–68^. However, the Fe-O_2_ adducts in these complexes are mostly unstable against irreversible O−O cleavage to yield Fe–O moieties, which are stabilized by H-bonding interactions. Herein, we report a ligand system, referred to as DIG_3_tren (tris(N’,N”-diisopropylguanidinyl-2-ethyl)amine), which simultaneously provides three neutral diisopropyl guanidine (DIG) donors to enforce a trigonal pyramidal coordination environment at a metal ion and positions three NH groups proximal to a metal center in the secondary coordination sphere. This ligand design allowed us to prepare an Fe(II) complex that performs a unique H-bond mediated two-electron transfer between Fe^II^ and end-on bound O_2_ to form a rare example of an iron(IV)-peroxido complex in a reversible manner (Fig. 1d). The steric shielding and H-bond donors of the second coordination sphere in [Fe^II^DIG_3_tren] also enabled reversible CO binding. With an *M* value (*K*_eq(CO)_/*K*_eq(O2)_) of 0.6, which is an index of the discrimination between oxygen and carbon monoxide bindings^46^, [Fe^II^DIG_3_tren] has a higher affinity for O_2_ than for CO. [Fe^II^DIG_3_tren], therefore, represents a suitable Hb/Mb and Hr model complex (for Hb, *M* = 234; for Hr, *M* = 0; Hr does not bind carbon monoxide)^69^, which, mediated by intramolecular H-bonds in the secondary coordination sphere, reversibly binds O_2_ in an end-on fashion with a high tolerance for CO.

## Results and discussion

### Synthesis and characterization of iron(II) complexes

**Fig. 2:**
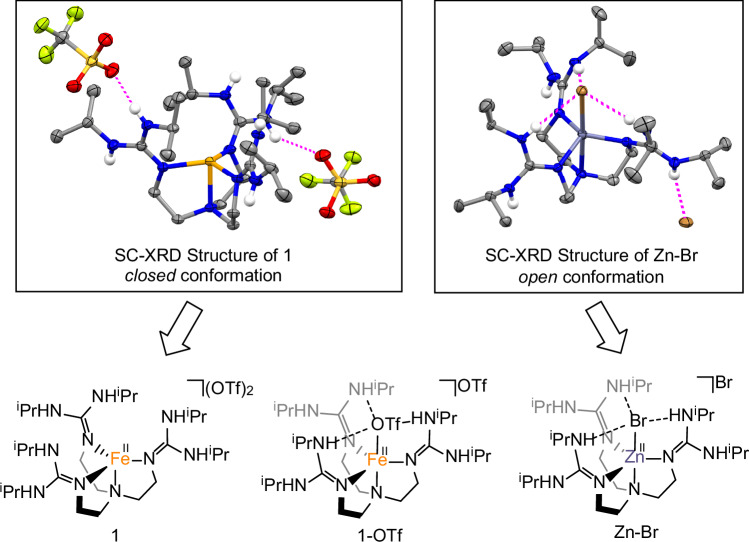
SC-XRD structures 1 and Zn-Br with schematic representations. Top: molecular structures of 1 (left) and Zn-Br (right) obtained by XRD. Atoms are displayed as thermal ellipsoids at 50% probability level; H atoms are omitted for clarity except nitrogen-bound H atoms. Structural parameters are summarized in Supplementary Table 1. Dashed lines represent H-bonding. (Atom types: Fe: orange; N: blue; C: gray; O: red; S: yellow; F: green; H: white; Zn: violet; Br: brown). Bottom: schematic representations of 1, 1-OTf and Zn-Br.

Combination of equimolar amounts of the reported ligand DIG_3_tren^70^ and [Fe(MeCN)_2_(OTf)_2_] (OTf=CF_3_SO_3_^−^) yields the complex [Fe^II^(DIG_3_tren)](OTf)_2_ (1, Supplementary Figs. 1–7), which shows a four coordinate trigonal pyramidal geometry (*τ*_4_ = 0.86)^71^ with the OTf groups involved in intra- and intermolecular H-bonding interactions with the guandine NH moieties (Fig. 2). Each triflate anion forms H-bonds with three complex molecules via the NH groups, creating a network of H-bonds (Supplementary Fig. 8). The zero-field Mössbauer spectrum (Supplementary Fig. 9) of solid 1 at 14 K reveals two quadrupole doublets with isomer shifts (δ), quadrupole splittings (Δ*E*_Q_) of 1.01, 2.98 mm/s and 0.97, 1.67 mm/s, respectively, consistent with the presence of two Fe(II) high-spin (*S* = 2) centers. Based on the Mössbauer parameters (δ = 1.00 mm/s; Δ*E*_Q_ = 1.66 mm/s, Supplementary Fig. 10) of the related [Fe^II^(TMG_3_tren)(OTf)](OTf) (TMG_3_tren = 1,1,1-tris{2-[N2-(1,1,3,3-tetramethylguanidino)]ethyl}amine) complex, we attribute the doublet with δ = 0.97 mm/s and Δ*E*_Q_ = 1.67 mm/s to [Fe^II^(DIG_3_tren)(OTf)](OTf) (1-OTf) with an axial OTf ligand. The doublet with δ = 1.01 mm/s and Δ*E*_Q_ = 2.98 mm/s then corresponds to the four-coordinate Fe(II) center observed in the X-ray structure of 1, which is also confirmed by Mössbauer measurements on the separated crystals of 1 (Supplementary Fig. 11). The four-coordinate iron center with vacant axial coordination site evident in the solid-state structure presumably contributes to the increased Δ*E*_Q_ value in 1. Expectedly, in the presence of a non-coordinating BF_4_^−^ anion, the four-coordinate species 1 is solely formed (because of strong H-bonding interaction), as evident from Mössbauer spectroscopy, which reveals a single quadrupole doublet with δ = 1.28 mm/s, and Δ*E*_Q_ = 3.01 mm/s (Supplementary Figs. 12 and 13). A four-coordinate iron site was also evident from the EXAFS analysis of powder samples of 1 (Supplementary Figs. 14 and 15, Supplementary Table 9). Although, single crystals suitable for X-ray diffraction studies could not be obtained for 1-OTf, the structure of the corresponding [Zn^II^(DIG_3_tren)(Br)]Br (Zn-Br) complex (Fig. 2) established the ability of the tetradentate DIG_3_tren ligand to enforce a trigonal bipyramidal coordination environment at a metal ion and position three guanidine NH groups for H-bonding interaction with an axially bound small molecule (Br^−^ in Zn-Br).

### Dioxygen reactivity and characterization of Fe-O_2_ adduct

**Fig. 3:**
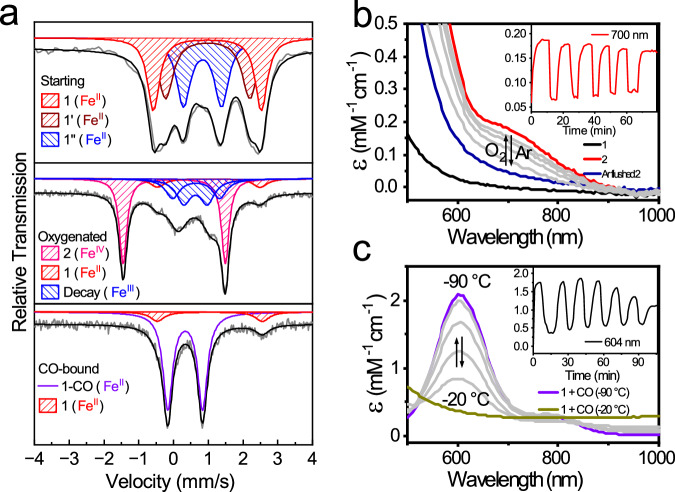
Mössbauer and UV/vis characterizations. **a** Top: zero-field Mössbauer spectrum of frozen acetone solution of 1 at 14 K (gray line: experimental spectrum; black line: simulated spectrum; red line: 1 with δ = 1.01 mm/s, Δ*E*_Q_ = 2.98 mm/s, 29%; brown line: 1’ with δ = 0.99 mm/s, Δ*E*_Q_ = 2.42 mm/s, 30%; blue line: 1” with δ = 0.83 mm/s, Δ*E*_Q_ = 1.10 mm/s, 41%); Middle: zero-field Mössbauer spectrum of an oxygenated acetone solution of ^57^Fe enriched 1/1-OTf at 14 K (gray line: experimental spectrum; black line: simulated spectrum; blue lines: Fe(III) decay products with δ = 0.65 mm/s, Δ*E*_Q_ = 1.35 mm/s, 20% and δ = 0.63 mm/s, Δ*E*_Q_ = 0.68 mm/s, 25%; pink line: 2 with δ = 0.01 mm/s, Δ*E*_Q_ = 2.94 mm/s, 46%; red line: residual 1, 9%). Bottom: zero-field Mössbauer spectrum of a CO flushed acetone solution of ^57^Fe enriched 1/1-OTf at 14 K (gray line: experimental spectrum; black line: simulated spectrum; purple line: 1-CO with δ = 0.34 mm/s, Δ*E*_Q_ = 1.00 mm/s, *Γ* = 0.33 mm/s, 88%; red line: 1, *Γ* = 0.50 mm/s, 12%). **b** UV/vis spectra showing the reversible formation of 2 by oxygen and argon flushing of 1/1-OTf in acetone at −80 °C, with time trace of the band at 700 nm shown as an inset. **c** UV/vis spectra of the temperature-dependent binding of CO to 1/1-OTf to form 1-CO, with the time trace of the band at 604 nm shown as an inset.

**Fig. 4:**
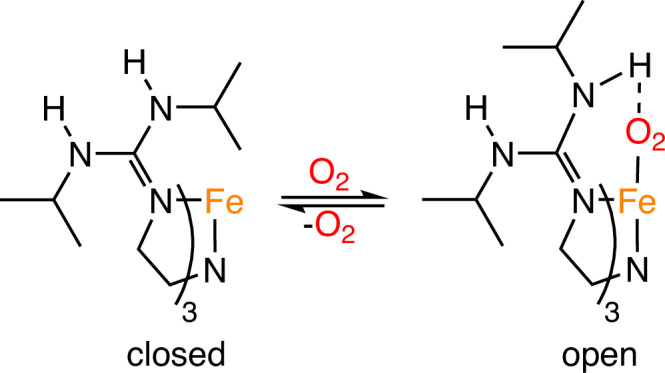
Scheme of proposed ligand-framework motion prior to O_2_ binding in 1. Preorganization of the N–H groups in 1 enables hydrogen-bond-assisted stabilization of the peroxido intermediate 2 upon O_2_ binding, whereas altered hydrogen-bonding environments in acetone prevent productive O_2_ reduction and instead leads to Fe(III) decay products.

While the four coordinate iron(II) center of 1 would appear to be well set up to bind O_2_, the stabilization of the bound OTf ligand by H-bonding interaction in 1-OTf (as also observed recently by Szymczak et al.^63^) may obstruct access of O_2_ to the Fe-site, thereby impacting its binding ability. The ^19^F-NMR spectrum (Supplementary Fig. 3) of a solution of 1/1-OTf in acetone, however, displayed a single resonance at −76.0 ppm, which confirmed that OTf is not bound to the Fe-center. Thus, in solution the Fe-center stays either four-coordinate with vacant axial coordinate site or five-coordinate with an axial acetone ligand (Supplementary Fig. 16). The Mössbauer spectrum of a frozen acetone solution of 1/1-OTf (Fig. 3a, top) reveals in addition to the quadruple doublet with δ = 1.01 mm/s, and Δ*E*_Q_ = 2.98 mm/s, corresponding to 1 (29%), two additional doublets (1’, δ = 0.99 mm/s, and Δ*E*_Q_ = 2.42 mm/s (30%) and 1”, *δ* = 0.83 mm/s, and Δ*E*_Q_ = 1.10 mm/s (41%)), which are also assigned to Fe(II) high-spin (*S* = 2) centers associated with the different conformational isomers of 1. The formation of different species in solution is attributed to the intrinsic flexibility of the DIG_3_tren ligand, where, as demonstrated previously^70^, the isopropylamino group from each DIG moiety acts as a flap that can either close (closed conformation) over iron or rotate away from iron (open conformation) to open up a site for auxiliary ligand binding (Fig. 4). Based on their similar Mössbauer parameters, the structure of complex 1’ can be considered identical to 1 with a four-coordinate Fe(II) center and containing all the DIG moieties in a closed conformation (Supplementary Fig. 16); the slight differences in δ and Δ*E*_Q_ values may presumably be attributed to the differences in the secondary coordination sphere, involving H-bonding interactions with acetone and OTf anion. In contrast, the Mössbauer parameters of 1” with lower Δ*E*_Q_ values represent a more symmetric Fe(II) center, similar to that observed for 1-OTf. Accordingly, a five-coordinate trigonal bipyramidal structure similar to Zn-Br (Fig. 2) is proposed for 1” ( Supplementary Fig. 16) with an axial acetone ligand; it is presumably a conformational isomer, where one or more DIG moieties exist in the open conformation, thus enabling the binding of solvent.

Interestingly, Mössbauer studies (Fig. 3a top and middle) reveal that upon bubbling of O_2_ through an acetone solution of a mixture of 1, 1’ and 1” at −95 °C, the five-coordinate Fe(II) species 1” with Δ*E*_Q_ < 2.0 mm/s that corresponds to 41% of Fe(II) in solution perform fast irreversible electron transfer process to yield two high-spin iron(III) decay products (Fig. 3a middle, green traces, 45%) with δ = 0.65 mm/s, Δ*E*_Q_ = 1.35 mm/s and δ = 0.63 mm/s, Δ*E*_Q_ = 0.68 mm/s. We propose that the Fe(II) centers with at least one of the DIG groups in open conformation react rapidly with O_2_ to form a transient superoxido species that undergoes oxidative decomposition via a ligand-centered hydrogen-atom abstraction process (Supplementary Fig. 17). This assignment is supported by ESI-MS analysis, which reveals three signals that are sensitive to ^16/18^O isotope-labeling (Supplementary Fig. 18). Of these, two signals at *m/z* = 744.4 and 297.7 are assigned to [Fe(O)(DIG_3_tren-H)(OTf)]⁺ and [Fe(O)(DIG_3_tren-H)]²⁺ (calcd. *m/z* = 744.37 and 297.71) ions, consistent with the formation of an alkoxoiron(III) species. A third signal at *m/z* = 594.4 corresponds to the dehydrogenated product [Fe(O)(DIG_3_tren-2H)]⁺ (calcd. *m/z* = 594.41). Together, these Fe(III) products indicate ligand-based hydrogen-atom abstraction coupled to O–O bond cleavage, accounting for the rapid oxidative decomposition observed for Fe(II) species derived from 1” in solution.

The four coordinate complexes 1 or 1’, with large Δ*E*_Q_ values of 2.98 and 2.42 mm/s, respectively, represent 59% of Fe(II) in solution and contain all three DIG moieties in closed conformation, as evident from the crystal structure of 1 (Fig. 2). They are converted slowly (*t* = 4200 s) to an equilibrium mixture of a red intermediate 2 (Fig. 3a middle, pink trace, 46%; Fig. 3b; *λ*_max_ = 700 nm; *ε* = 0.20 mM^−1^ cm^−1^; half-life at −70 °C = 100 s) with δ = 0.01 mm/s and Δ*E*_Q_ = 2.94 mm/s and residual 1 (9%). The low δ value of 2 is strongly indicative of an iron(IV) complex, as it is similar to that of the Fe^IV^ site of [Fe^IV^(O)(6-Me_3_tpa)(μ-O)Fe^III^(6-Me_3_tpa)(H_2_O)] (0.10 mm/s)^72^ and [Fe^IV^(O)(TMG_3_tren)]^2+^ (0.09 mm/s)^73^, reflecting the nitrogen-rich ligand environment of 2. Remarkably, bubbling of Ar through the acetone solution of 2 for 2 min at −95 °C regenerates 1, showing that 2 is a reversible Fe−O_2_ adduct; several cycles of alternating Ar/O_2_ purges can be achieved (Fig. 3b and Supplementary Fig. 19).

**Fig. 5:**
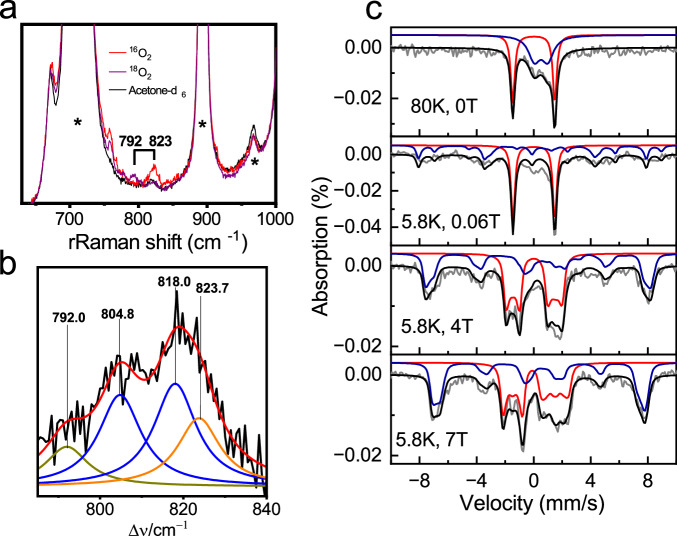
rRaman and Mössbauer characterizations of 2. **a** rRaman spectra (−90 °C, λ_exc_ = 568 nm) of acetone-d_6_ (black) and 2 prepared at −90 °C in acetone-d_6_, generated by using ^16^O_2_ (red) and ^18^O_2_ (purple). **b** Band fitting analysis of the spectral region between 785 and 840 cm^−1^ of 2, prepared at −90 °C in acetone-d_6_ using a mixture of ^16^O_2_, ^18^O_2_, ^16^O–^18^O. The weak band of the solvent at 817 cm^−1^ was subtracted from the experimental spectrum (black trace). Four Lorentzian functions were fitted to the experimental spectrum, representing the complexes with ^16^O_2_ (orange), ^16/18^O_2_ and ^18/16^O_2_ (blue), and ^18^O_2_ (dark yellow). The red trace shows the cumulative fit. Details of the fitting procedure are given in the SI. **c** Mössbauer spectra of 2 (red component) recorded at 80 K without applied magnetic field or at 5.8 K in a magnetic field of 0.06, 4, or 7 T applied parallel to the direction of the γ-rays. Dark blue lines correspond to the Fe^III^ decay products formed during the reaction. Spin-Hamiltonian simulation parameters in Supplementary Table 2.

The samples were analyzed by applied-field Mössbauer spectroscopy (Fig. 5c), with the precursor complex being measured separately in acetone and subtracted from the spectrum obtained after reaction of the mixture with O_2_ (Fig. 3a, top). After subtraction of the minor amount of the remaining precursor, the spectra were simulated with two species: an integer-spin (*S* = 1) species corresponding to 2 that yields a doublet in the low-field spectra, and a half-integer spin (*S* = 5/2) corresponding to the high-spin Fe^III^ decay product that yields a six-line pattern. The splitting of the six-line pattern matches to what is expected for an *S* = 5/2 Fe(III) center. The 80 K spectrum was simulated with two doublets with parameters δ = 0.01 mm/s, Δ*E*_Q_ = +2.94 mm/s (2, red line) and δ = 0.51 mm/s, Δ*E*_Q_ = 0.94 mm/s (green line), consistent with Fe(IV) and high-spin Fe(III), respectively. The faint, broad absorptions under the baseline indicate that all the paramagnets of the *S* = 5/2 species have not switched to fast relaxation at 80 K, pointing to the formation of aggregates. The fitted value of the hyperfine coupling constant for 2 (Fe^IV^) falls well within the expected range, which confirms the *S* = 1 ground state^3,74–77^. The large value for the zero-field splitting (*D* = +20.8 cm^−1^) is consistent with other *S* = 1 Fe^IV^=O complexes, and implies a relatively well isolated Ms = 0 ground state within the *S* = 1 multiplet. This brings up the question of the possibility of an *S* = 0 ground state. A fit with an *S* = 0 spin Hamiltonian, however, fails to accurately reproduce the positions of the absorptions in the high-field spectra. We can therefore confidently assign the ground spin state as *S* = 1 for 2.

The presence of a peroxido moiety in 2 is fully consistent with resonance-Raman (rRaman) measurements, as shown in Fig. 5a. A vibrational mode at 823 cm^−1^ is observed that shifts to 792 cm^−1^ upon ^18^O isotopic labeling. The frequency of 823 cm^−1^ is in line with the values expected for an O–O stretching of an iron-peroxido in side-on or end-on binding modes or the Fe=O stretching of an iron-oxido species^10,13,15,17,44,78–81^. To evaluate these possibilities, complex 2 was generated with mixed isotope O_2_ (^16,18^O_2_), corresponding to a mixture of ^16^O_2_, ^16/18^O_2_, and ^18^O_2_. If the 823 cm^−1^ band is an O–O stretching mode, one will expect, in addition to the bands at 792 (^18^O_2_) and 823 cm^−1^ (^16^O_2_), one or two further bands around 810 cm^−1^^82,83^. While for the symmetrical side-on binding mode, ^16^O^18^O and ^18^O^16^O stretches are expected to appear at identical energies (one band), they will be different for the end-on mode (two bands). For an Fe=O vibration, the ^16,18^O_2_ spectrum will lack any band at ca. 810 cm^−1^. Figure 5b shows the rRaman spectrum of the active species prepared with ^16,18^O_2_. The region of the oxygen-sensitive modes between 785 and 840 cm^−1^ clearly involves at least three bands, such that an assignment to a Fe=O species can be ruled out. Thus, O_2_ must be bound to the Fe in the complex. Although three Lorentzian functions provide a mathematically good fit, it does not reproduce the bands of the ^16^O_2_- and ^18^O_2_-bound species (further details of the band fitting analysis are given in the SI; Supplementary Fig. 20). Thus, a side-on complex can be discarded as well. A similar good fit is achieved on the basis of four Lorentzians (Fig. 5b). Here, the bands of the ^16^O_2_- and ^18^O_2_-bound species are very well reproduced and also the two additional bands have the expected frequencies at 804.8 and 818.0 cm^*−*1^ of the Fe(^16^O–^18^O) and Fe(^18^O–^16^O) vibrational modes of an end-on [Fe^IV^(η^1^-O_2_)(DIG_3_tren)]^2+^ moiety in 2.

Additional evidence for the end-on binding mode is demonstrated by extended X-ray absorption fine structure (EXAFS) data (Supplementary Figs. 14 and 15), which for 2 are compatible with the presence of iron-oxygen distances of about 1.83 and 2.76 Å due to an end-on peroxido species, respectively (Supplementary Table 9). The K-edge energy of 2 is in between the energies of the Fe(II) (1) and Fe(IV) (see below) containing complexes, which may reflect the presence of Fe(III) decay products, as seen in the Mössbauer spectrum.

**Fig. 6:**
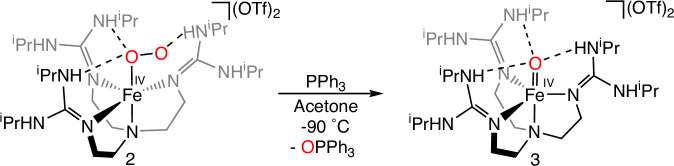
Oxygen atom transfer reactivity of 2. Reaction of 2 with triphenylphosphine in acetone at −90 °C, forming complex 3 and triphenylphosphine oxide.

To additionally verify the presence of the two oxygen atoms in 2, the reaction of 2 with 30 equivalents of triphenylphosphane (PPh_3_) was investigated at −90 °C in acetone (Fig. 6), leading to the quantitative formation of an iron(IV)-oxido intermediate 3 and triphenylphoshane oxide (O=PPh_3_) (Supplementary Fig. 21). To verify the nature of 3, we synthesized it directly from 1/1-OTf using artificial oxygen atom donor reagents and characterized it spectroscopically (see below).

**Fig. 7:**
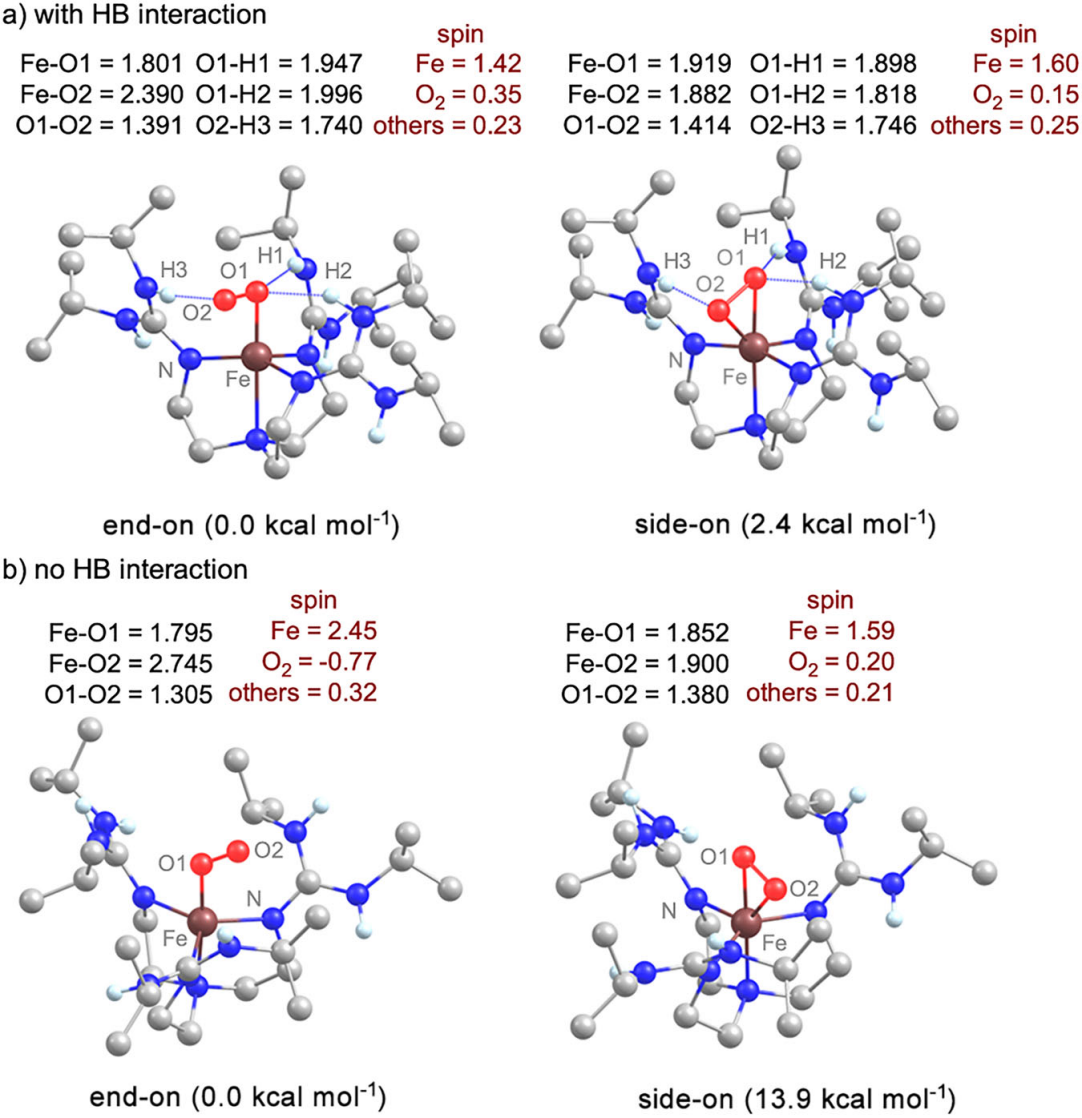
DFT calculated structure of 2. **a** Optimized structures of [Fe(O_2_)DIG_3_tren]^2+^ in the presence of H-bonding. **b** Optimized structures of [Fe(O_2_)DIG_3_tren]^2+^ in the absence of H-bonding. Calculations were done at the UBP86-D3(BJ)/def2-TZVPP level in solvent. Hydrogen atoms of the ligand are omitted for clarity, except nitrogen-bound H atoms. Lengths are in Å units. Values in parentheses are Gibbs free energies.

Density functional theoretical (DFT) calculations were performed to investigate the electronic and geometric structures of the [Fe^IV^(O_2_)(DIG_3_tren)]^2+^ moiety in 2 in the presence (Fig. 7a) and absence (Fig. 7b) of H-bonding (HB) interaction. The ground spin state is a triplet state (*S* = 1), which is consistent with the experimental results. All side-on isomers lie higher in energy than the end-on isomer. The H-bonding interaction reduces the energy gap of the end-on and the side-on isomers from 13.9 to 2.4 kcal mol^−1^. With the three H-bondings (a strong one with a (N)H-O_distal_ distance of 1.74 Ă and two weak ones with (N)H-O_proximal_ distances of 2.02 and 1.92 Å; Supplementary Figs. 22 and 23), the end-on isomer lies 3.2 kcal mol^−1^ lower than the side-on isomer. It is important to note that H-bonding to the distal oxygen atom is necessary to stabilize the experimentally determined end-on structure; when only the proximal oxygen atom takes part in H-bonding, a side-on binding mode of O_2_^2−^ is stabilized (Supplementary Fig. 24). The calculated Fe–O (1.802 Å) and O–O (2.391 and 1.391 Å) distances of the end-on peroxido complexes are in good agreement with the experiment. The spins of the iron and the dioxygen moieties are 1.42 and 0.33, respectively. Without the H-bonding interaction, altered spin densities of 2.45 and −0.78 are observed at iron and oxygen, respectively. The increase of approximately one spin on iron and the location of nearly one spin on the dioxygen moiety demonstrates that H-bonding interaction causes the modification of the electronic structure of the iron-dioxygen intermediate from an iron(III)-superoxido species to an iron(IV)-peroxido structure. Thus, a second electron transfer from Fe(III) to O_2̄_ ®^⦁^ is coupled to the H-bonding stabilization of the resultant O_2_^2−^ moiety (Fig. 1c). Notably, a similar proton coupled electron-transfer process is reported in Hr (Fig. 1b) and model Cu and Mn complexes, which effects a superoxo-to-hydroperoxo conversion^51,53,54,84–86^.

### Spectroscopic characterization of 3

**Fig. 8:**
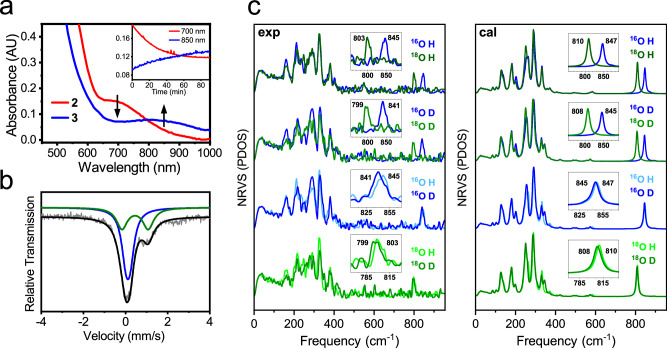
UV/vis, NRVS, and Mössbauer characterizations of 3. **a** UV/vis spectrum of the reaction of 2 with 30 equivalents of PPh_3_ in acetone at −90 °C with time traces as inset. **b** Zero-field Mössbauer spectrum of the reaction of 2 with 30 equivalents of PPh_3_ in acetone at −90 °C (gray line: experimental spectrum; black line: simulated spectrum; blue line: 3 with δ = 0.09 mm/s; Δ*E*_Q_ = 0.17 mm/s, *Γ* = 0.45 mm/s, 50%; green line: decay product with δ = 0.45 mm/s, Δ*E*_Q_ = 1.24 mm/s, *Γ* = 0.60 mm/s, 50%). **c** Experimental (left) and calculated (right) NRVS spectra of 3 (^16^O H), ^18^O labelled 3 (^18^O H), 3-d_6_ (^16^O D) and ^18^O labelled 3-d_6_ (^18^O D) generated by reacting 1 or 1-d_6_ with ^s^PhI^16^O and ^s^PhI^18^O (spectra are vertically stacked for clarity; insets: magnified Fe=O vibration bands). Calculated NRVS spectra corresp to a general structure with 4 equatorial N-ligands at Fe(IV) and an axial Fe=O bond opposite to a vacant axial site.

Reaction of 1/1-OTf with 2-(tert-butylsulfonyl)iodosobenzene (^s^PhIO) or tetrabutylammonium periodate (Bu_4_NIO_4_) in acetone at −70 °C also yielded the temperature sensitive orange species 3 (half-time at −20 °C = 270 s) with absorption maxima at *λ*_max_ = 395 nm (*ε* = 8.92 mM^−1^ cm^−1^) and λ_max_ = 805 nm (*ε* = 0.25 mM^−1^ cm^−1^) with no apparent intensity changes observed within 30 min at −70 °C. The ^19^F-NMR spectrum of 3 at 203 K (Supplementary Fig. 25) shows only one singlet at δ = −77.73 ppm, which is typical of free triflate. In conjunction with the fact that the UV/vis spectra of 3 in both acetone (typically non-coordinating) and acetonitrile (coordinating) are identical (Supplementary Fig. 26) in the relevant range (350 to 1000 nm), it is confirmed that no other exogenous ligands are bound at the iron center. This indicates a five-coordinate geometry for 3, leading to the formulation as [Fe^IV^(O)(DIG_3_tren)]^2+^. It is worth noting that at −70 °C, 3 shows no reaction with PPh_3_, allowing its isolation in the reaction of 2 and PPh_3_ by UV–vis (Fig. 8a) and Mössbauer (Fig. 8b) spectroscopic methods. The latter reaction, when followed by Mössbauer spectroscopy (Fig. 8b) reveals the formation of a new doublet (δ = 0.09 mm/s; Δ*E*_Q_ = 0.17 mm/s, 50%) with the concomitant disappearance of the doublet corresponding to 2. The low isomer shift of 3 suggests another iron(IV) species formed, and the low quadrupole splitting is consistent with other trigonal bipyramidal iron(IV)-oxido complexes in the *S* = 2 spin state^16^. The greatly reduced reactivity of 3 towards OAT is plausibly attributed to the steric shielding and H-bond stabilization of the Fe^IV^=O core by the secondary coordination sphere in [Fe^IV^(O)DIG_3_tren]. This is also supported by the calculated DFT structure of 3 that shows three short N−H···O distances of approximately 1.7 Å, indicative of strong hydrogen-bonding interactions (Supplementary Fig. 27).

The zero-field Mössbauer spectrum of 3 (Supplementary Fig. 28) generated in the reaction of 1/1-OTf with sPhIO reveals a major species (δ = 0.09 mm/s; Δ*E*_Q_ = 0.17 mm/s) with the same parameters as that obtained in the OAT reaction of 2 and PPh_3_ (Fig. 8b). High magnetic field Mössbauer spectra (Supplementary Fig. 29) confirm the assignment of the *S* = 2 ground state of 3 with a typical small zero field splitting (*D* = +5.17 cm^−1^). The minor doublet is best simulated with *S* = 0. The magnetic measurements also reveal a third species with similar features to the *S* = 5/2 component of 2. It is therefore also assigned to high spin Fe(III). The Mössbauer parameters of the *S* = 0 component are similar to the latter. An assignment as dimeric high spin Fe(III) would explain both the Mössbauer parameters and the *S* = 0 spin multiplicity.

The trigonal-bipyramidal geometry for 3 is also supported by XAS analysis (Supplementary Figs. 14 and 15), where a short iron-oxygen bond length of ca. 1.64 Å was obtained, typical of an iron(IV)-oxido double bond and in good agreement with the DFT value (1.69 Å; Supplementary Tables 9 and 10). The difference in Fe–O bond lengths between 2 and 3 highlights the distinction between the iron peroxido and iron oxido units in 2 and 3, respectively. The average Fe–N distances in both complexes (2.00–2.02 Å), which belong to the N donors of DIG_3_tren, are likewise consistent between experiment and theory. The Fe K-edge X-ray absorption spectrum (Supplementary Fig. 14) of 3 shows an edge energy of 7123.1 eV (vs. 7120.4 eV for 1 and 7122.4 for 2), which supports an Fe^IV^ oxidation state. Nuclear resonance vibrational spectroscopy (NRVS) shows an Fe=O vibration mode for 3 (Fig. 8c) at 845 cm^−1^ that shifts to 803 cm^−1^ by ^18^O isotopic labeling, thereby clearly confirming the presence of an iron-oxido unit.

In order to experimentally verify the H-bonding interaction of the Fe^IV^=O moiety in 3 with the DIG_3_tren ligand, the deuterated complex [Fe(DIG_3_tren-d_6_)](OTf)_2_ (1-d_6_) was synthesized with the -NH groups of the DIG_3_tren ligand being deuterated (N−D). Subsequently, 3-d_6_ was synthesized in acetone-d_6_ by following a procedure analogous to that of the synthesis of 3. A comparison of the self-decay kinetics of 3 at −20 °C in acetone (*t*_1/2_ = 270 s), in acetone-d_6_ (*t*_1/2_ = 381 s) and 3-d_6_ in acetone-d_6_ (*t*_1/2_ = 558 s) shows a significant increase in the stability of 3 upon deuteration of the ligand and the use of deuterated solvent (Supplementary Fig. 30) with a two-step kinetic profile. This reveals that the decay of 3 occurs via hydrogen atom abstraction (HAA) of one of the amine groups, providing evidence for close proximity of the N−H moieties to the oxido unit due to H-bonding. Electrospray ionization mass spectrometry recorded after completion of the decay shows signals at *m/z* = 744.3 and 297.7, assigned to [Fe(O)(DIG_3_tren-H)(OTf)]⁺ and [Fe(O)(DIG_3_tren-H)]²⁺, respectively (calcd. *m/z* = 744.37 and 297.71). Both species are lighter by one hydrogen atom relative to 3 and shift accordingly upon use of ^s^PhI¹⁸O, confirming retention of the oxido ligand (Supplementary Fig. 31). The formation of −1 H products is consistent with intramolecular hydrogen-atom abstraction by the oxido unit, as reported for the related iron(IV)–oxido complex [Fe^IV^(O)(TMG_3_tren)]^2+^. Although an N–H group is involved in initiating the decay, the final product reflects oxygenation at a carbon site within the secondary coordination sphere, yielding an alkoxylated ligand framework (Supplementary Fig. 32).

The presence of H-bonds to the oxido ligand was also confirmed by NRVS data (Fig. 8c). The iron oxido vibration was shown to down-shift by about 4 cm^−1^ upon deuteration of the -NH groups of 3 in 3-d_6_, both in the ^16^O and ^18^O complexes (Fig. 8c), similar to the DFT predicted shifts of ca. 2 cm^−1^. This small shift may be expected since the iron-oxido vibration is likely only slightly influenced by the presence of H-bonds.

Although 1-d_6_ could be converted to 3-d_6_ in the presence of ^s^PhIO, its reaction with O_2_ in acetone-d_6_ led to the formation of 2 in significantly reduced yields (5%; Supplementary Fig. 33). Notably, H/D kinetic isotope effects (KIEs) are expected to be pronounced when quantum tunneling is of importance^87,88^, such as in the case of low-temperature binding of O_2_ to 1 involving the motion of hydrogen/deuterium atoms (from a position where the N–H/D groups point away from the O_2_ binding site in 1 (closed conformation) to that in 2 where N–H/D points towards the O_2_ binding site (open conformation); see Fig. 4). In addition to affecting the kinetics of O_2_ binding, deuteration of a hydrogen bond by replacing protium (H) with deuterium (D) can cause geometric changes in the hydrogen bond, known as the geometric H/D isotope effect (GIE)^89,90^. Upon deuteration, the donor acceptor distances in the N–H---O_2_^2−^ hydrogen bonds in 2 is expected to increase (“normal” Ubbelohde effect)^91,92^, which may also contribute to the mitigated access of O_2_ to the Fe^II^ center in 1.

### Binding of carbon monoxide

We also examined the reaction of 1 with CO to detect any iron−carbonyl intermediate similar to carboxyhemoglobin^40,46,93,94^. An acetone solution of 1, when treated with CO at −90 °C, results in the formation of a blue intermediate 1-CO (*λ*_max_ = 604 nm; *ε* = 2.05 mM^−1^ cm^−1^). CO coordination to the metal center occurs without a change in oxidation state, but induces a spin-state transition from high-spin (*S* = 2) to low-spin (*S* = 1)^95–97^ as evident from the zero-field Mössbauer spectroscopy (Fig. 3a bottom). The Mössbauer spectrum reveals one major doublet (δ = 0.34 mm/s; Δ*E*_Q_ = 1.00 mm/s, 88%) corresponding to 1-CO and some residual 1 (12%). ^1^H-NMR spectrum of 1-CO (Supplementary Fig. 34) shows paramagnetically shifted bands confirming the presence of a *S* = 1 Fe(II) low-spin center in a TBP geometry. The resonance Raman spectrum (in acetone at −90 °C upon excitation at 568 nm) shows the CO vibration at 1975 cm^−1^, which disappears when the sample is warmed to room temperature (Supplementary Fig. 35); it reappears again upon exposure to CO at −90 °C. Notably, the *ν*_CO_ stretching frequency of 1-CO is slightly higher than that of carboxyhemoglobin (1951 cm^−1^)^98^. DFT calculations on the Fe(II)−CO adduct show that CO binds in a linear fashion with the presence of three hydrogen bonds from N–H donors in the secondary coordination sphere. The calculated N−H···O(CO) distances (ca. 2.3–2.4 Å) indicate weak hydrogen-bonding interactions that do not significantly perturb the Fe–C–O geometry (Supplementary Fig. 36). The calculated *ν*_CO_ vibration at 1896 cm^−1^ is in good agreement with the experiment. As the temperature is increased, the absorption band at 604 nm due to 1-CO decreased (Fig. 3c). This process is reversible in the temperature range −90 to −20 °C. The equilibrium constant (*K*_eq(CO)_) was determined (Supplementary Figs. 37 and 38) to be 0.09 mM^−1^ at −60 °C. Interestingly, under similar conditions, 1 binds O_2_ with a *K*_eq(O2)_ of 0.15 mM^−1^. This gives a *M* value of 0.6, which is an index of the discrimination between oxygen and carbon monoxide bindings. Thus, the secondary coordination sphere provided by the DIG_3_tren ligand functions similarly as the distal histidine in Hb/Mb; it provides an ideal environment for strong H-bonds (N−H···O distances of 1.73, 1.95, and 2.00 Å; Fig. 7) with bound oxygen in [Fe(η^1^-O_2_)(DIG_3_tren)]^2+^, but out of place for optimal interaction with a linear bound CO (very weak N−H···O(CO) H-bonding interactions with 2.37, 2.36, and 2.40 Å; Supplementary Fig. 36a for ^3^1-CO) in [Fe(CO)(DIG_3_tren)]^2+^. This is believed to be crucial for increasing the dioxygen affinity for [Fe^II^DIG_3_tren] compared to carbon monoxide.

This study establishes a new milestone in connecting H-bonding with reversible oxygen and carbon monoxide bindings in a mononuclear nonheme system, demonstrating an unusual iron mediated two-electron reduction of oxygen. Peroxide dianion is known to form strong hydrogen bonds to N–H groups from the hexacarboxamide cryptand^99^ or a zinc tripod^65^, which has led to two-electron reduction of dioxygen in the presence of an external reductant like cobaltocene. Closely related hydrogen-bond-assisted two-electron O_2_/H_2_O_2_ interconversion has also been demonstrated in saddle-distorted porphyrin/isophlorin redox couples^100^. In this report, we have now extended this effect to iron-dioxygen chemistry. DFT studies suggest that three H-bonding interactions between the O_2_^2−^ dianion and N–H groups from the DIG_3_tren ligand sufficiently reduce the barrier for the two-electron reduction of O_2_ by Fe(II) to form a rare nonheme Fe(IV)-peroxido intermediate (*S* = 1) in the reaction of 1 with dioxygen. The two-electron transfer from Fe^II^ to O_2_ is further facilitated by the electron-rich nature of [Fe(DIG_3_tren)]^2+^. This is evident from the low Fe^III/II^ potential of +0.17 V vs. Fc^+/o^ for 1 and a Fe^IV/III^ reductive wave for 3 at a large negative potential with *E*_p,c_ of −1.63 V vs. Fc^+/o^ (determined by cyclic voltammetry; Supplementary Figs. 39 and 40), and can be attributed to the excellent donor quality of DIG_3_tren containing the superbasic guanidine moieties^101^. In the presence of none or weak H-bonding interaction, one-electron chemistry would prevail, as has been previously observed in the stabilization of the iron(III)-O_2_^−•^ complexes in the Fe(II) and O_2_ reactions^34–36,38,66^. Notably, the ability to reversibly bind O_2_ is unique for four coordinate 1 in the presence of OTf anion, where all the DIG arms exist in the closed conformation (Fig. 2). The stabilization of the Fe(IV)-peroxido intermediate, however, warrants the conversion of the DIG arms into the open conformation (Figs. 4 and 7). Accordingly, O_2_ binding to the Fe(II) center and the isomerization of the DIG arms must occur at comparable rates, so that the transient Fe(III)-superoxido intermediate is converted to the more stable Fe(IV)-peroxido form via a H-bonding mediated electron-transfer process. Significant increase in the O_2_ binding rate relative to the ligand reorganization rate (for example, in the open conformation of one or more DIG units of the DIG_3_tren ligand), will allow the oxidative decomposition of ligand by the transient Fe(III)-superoxido intermediate, and complex 2 will be inaccessible. To further probe the role of the counterion, we examined complexes bearing weakly coordinating anions. While the solid-state Mössbauer spectrum of 1-BF_4_ shows a single Fe(II) species closely resembling 1, this complex does not form intermediate 2 upon exposure to O_2_, as evidenced by UV/vis spectroscopy (Supplementary Fig. 41). This behavior is attributed to stronger hydrogen-bonding interactions between BF_4_^−^ and the DIG_3_tren ligand, which disrupt the secondary-sphere preorganization required for peroxido stabilization (Fig. 4). In contrast, complexes containing truly non-coordinating anions (1-BPh_4_ and 1-NTf_2_) display multiple Fe(II) environments in the solid state, consistent with the increased conformational flexibility of the ligand scaffold, and likewise fail to form 2 (Supplementary Figs. 42–45). Collectively, these results highlight the unique role of the triflate anion: it is sufficiently weakly coordinating to leave the axial site accessible for O_2_, yet capable of preorganizing the hydrogen-bonding network in a manner that selectively stabilizes the iron(IV)-peroxido intermediate (Fig. 4). Deuteration of the N–H group in the DIG moiety led to the formation of 2 in a reduced yield of 5%, giving an N–D/N–H kinetic isotope effect of 0.11. This may further corroborate the motion of hydrogen/deuterium atoms during the formation of 2; the rate of DIG_3_tren-d_6_ rearrangement involving heavier N–D moieties becomes slower relative to O_2_ binding, yielding 2 in significantly reduced yields. Thermodynamic parameters for O_2_ binding were obtained from a van’t Hoff analysis. Owing to the limited thermal stability of intermediate 2 above −80 °C, equilibrium constants could only be determined over a narrow temperature range (−95 to −80 °C in acetone). The resulting van’t Hoff plot (Supplementary Fig. 46) yields Δ*H*° = −10.66 ± 0.37 kJ mol^−^^1^ and Δ*S*° = −16.5 ± 2.0 J mol⁻¹ K⁻¹. The negative values of Δ*H*° and Δ*S*° are consistent with the binding of O_2_ only at low temperature. Furthermore, a significantly negative Δ*S*° term may corroborate an associative mechanism characterized by a well-ordered transition state (TS).

The reversibility of the dioxygen binding is a remarkable property of 2, typically observed in mononuclear heme or dinuclear nonheme systems. The low *M* value of 0.6 further shows the importance of H-bonds in relation to the preference for oxygen bonding vs. carbon monoxide bonding. As has been proposed in hemoproteins^102–106^, the presence of weak secondary H-bond donors does not affect significantly the energy of the linear Fe-CO unit, concomitantly increasing the O_2_ affinity of the intrinsically bent Fe-O_2_ group. Nevertheless, the near-quantitative formation of a single Fe(II)–CO adduct upon exposure to CO demonstrates that all Fe(II) centers in acetone solution (corresponding to 1, 1’, and 1”; Supplementary Fig. 16) are accessible for O_2_/CO binding. The CO coordination proceeds without electron transfer, preserving the Fe(II) oxidation state and preventing ligand oxidation; consequently, all conformers converge cleanly to 1-CO (Fig. 3a, bottom). In contrast, the productive two-electron reduction of O_2_ occurs only for a specific isomer in which all N–H hydrogen-bond donors stabilize the bound O_2_^2−^ species, thereby enabling formation of intermediate 2. For other isomers lacking this preorganized hydrogen-bonding environment, O_2_ binding instead results in a highly reactive Fe(III)-superoxido species, which rapidly induces ligand oxidation, resulting in reduced yields for 2. Complex 2 reacts with PPh_3,_ leading to O–O bond cleavage and subsequent formation of a high-valent Fe(IV)-oxido species 3 (*S* = 2), which can also directly be synthesized from 1 using common oxygen atom transfer reagents. Thus, by elucidating the interplay between H-bonding and iron-based oxygen activation, our work expands the fundamental understanding of oxygen activation in biological and bioinspired systems, paving the way for novel catalyst design and biomimetic applications.

## Methods

### Chemicals and handling

Chemicals employed were purchased from the companies ABCR, ACROS, SIGMA-ALDRICH, MERCK, BLDpharm, and TCI, and used without further purification. Anhydrous solvents (acetonitrile, diethyl ether, dichloromethane and acetone) were purchased from CARL-ROTH GmbH under the tradename ROTIDRY (>99.5%, <50 ppm H_2_O), degassed by freeze-pump-thaw methods prior to use and stored over activated molecular sieves (except acetone). Deuterated solvents were purchased from EURISO-TOP. Preparation and handling of air or water sensitive compounds were performed under an inert atmosphere using either Schlenk techniques or a GS MEGA glovebox from GS GLOVEBOX Systemtechnik GmbH filled with N_2_. Nitrogen and Argon of quality 5.0 were used for this purpose and were purchased from AIR LIQUIDE. Commercially available O_2_ from AIR LIQUIDE (99.99% pure) and ^18^O_2_ (97.2% pure) from Euriso-Top were purchased and used as received.

### Elemental analysis

All elemental analyses were performed by the analytical service of the Institut für Chemie of the Humboldt-Universität zu Berlin. The percentages of carbon, hydrogen, nitrogen and sulphur were determined using an HEKAtech EURO EA 3000 analyzer.

### Nuclear magnetic resonance spectroscopy

All NMR spectra were recorded using a BRUKER AVANCE DPX 300 MHz or BRUKER AVANCE III 500 MHz spectrometer. Those of ^1^H, ^13^C, and ^19^F nuclei were recorded in deuterated solvents, and chemical shifts (ppm) referenced against residual protic solvent peaks. Unless otherwise stated, all spectra were recorded at room temperature.

### Electrospray ionization mass spectrometry

ESI-MS spectra of organic molecules and inorganic complexes in solution were recorded by using an ADVION EXPRESSION CMS spectrometer (under typical ionization conditions), and spectra in positive and negative mode were collected in parallel; acetonitrile was used as an eluent. For thermally unstable complexes, the freshly thawed solutions were directly injected into the instrument while the ionization source temperature was decreased to 50 °C. The analysis of the data was carried out with the ADVION DATA EXPRESS version 6.0.11.3.

### Gas chromatography

GC analysis was carried out by using an AGILENT 7890B gas chromatograph (HP5 25 column, 30 m) with a flame-ionization detector. GC-MS was performed on an AGILENT 5977B spectrometer with a triple-axis detector.

### Single crystal X-ray structure determinations

For the determination of the X-ray crystal structure of the complex, data collection was performed at 100 K on a BRUKER D8 VENTURE diffractometer by using Mo Kα radiation (λ = 0.71073 Å). Multi-scan absorption corrections implemented in SADABS^107^ were applied to the data. The structure was solved by the intrinsic phasing method (SHELXT 2014/5)^108^ and refined by full matrix least square procedures based on F2 with all measured reflections (SHELXL-2018/3)^109^ in the graphical user interface SHELXle^110^) with anisotropic temperature factors for all non-hydrogen atoms. All hydrogen atoms were added geometrically and refined by using a riding model.

### Mössbauer spectroscopy

Mössbauer spectra in the absence of magnetic field were recorded on a SEECO MS6 spectrometer that comprises the following instruments: a JANIS CCS-850 cryostat, including a CTI-CRYOGENICS closed cycle 10 K refrigerator, and a CTI-CRYOGENICS 8200 helium compressor. The cold head and sample mount are equipped with calibrated DT-670-Cu-1.4 L silicon diode temperature probes and heaters. Temperature is controlled by a LAKESHORE 335 temperature controller. Spectra are recorded using a LND-45431 Kr gas proportional counter with beryllium window connected to the SEECO W204 γ-ray spectrometer that includes a high voltage supply, a 10 bit and 5 μs ADC and two single channel analyzers. Motor control and recording of spectra is taken care of by the W304 resonant γ-ray spectrometer. For the reported spectra a RIVERTEC MCO7.114 source (^57^Co in Rh matrix) with an activity of about 1 GBq was used. All spectra were recorded in a plastic sample holder with a solid or frozen solution sample at 14 K, and data were accumulated for about 12–24 h. Spectra were simulated using the software WMOSS4^111^. Applied-field Mössbauer spectra were measured on an OXFORD INSTRUMENTS Spectromag 4000 cryostat containing an 8 T split-pair superconducting magnet. The spectrometer was operated in constant acceleration mode in transmission geometry, with the magnetic field applied parallel to the direction of the gamma rays. The isomer shifts are referenced against a room temperature metallic iron foil. Analysis of the data was performed using the in-house developed Python package easyMoss^112^. All spectra were modeled with slow relaxation.

### Resonance Raman (rRaman) spectroscopy

Resonance Raman spectra were measured in acetone-d_6_ at −90 °C (Bruker cryostat) with 568 nm excitation (Coherent cw Kr^+^-laser, 8 mW) using a Horiba Jobin-Yvon LabRAM HR800 confocal Raman spectrometer. The sample concentrations employed were 10–20 mM.

### Attenuated total reflection Fourier-transform infrared spectroscopy (ATR-FTIR)

IR spectra were measured at a Bruker ALPHA II FTIR spectrometer using the platinum ATR module (diamond crystal). Spectra were collected with a resolution of 4 cm^−1^ in the range of 350–4000 cm^−1^ and using 24 scans.

### UV–vis spectroscopy

The UV–vis absorption spectra were recorded with an 8453 UV–visible spectroscopy system from Agilent. The measurements were carried out in 10 mm precision cuvettes made of SUPRASIL® quartz glass, the closures of which were equipped with a septum. The measurements at low temperatures were carried out by cooling the cuvette holder using a cooling thermostat USP-203-A from Unisoku Scientific Instruments. The analysis of the spectra was carried out with the software UV–Visible Chemstation from Agilent.

### X-ray absorption spectroscopy

XAS at the Fe K-edge was performed at beamline KMC-3 at the BESSY-II synchrotron (Helmholtz Center Berlin, Germany) using a set-up including a Si[111] double-crystal monochromator, a 13-element energy resolving Si-drift detector (RaySpec) for X-ray fluorescence monitoring, and DXP-XMAP pulse-processing electronics (XIA). Samples were held at 20 K in a liquid-helium cryostat (Oxford). The energy axis of the monochromator was calibrated (accuracy ±0.1 eV) using the K-edge spectrum of an iron metal foil (fitted reference energy of 7112 eV in the first derivative spectrum). The spot size on the samples was ca. 1.5 × 3.0 mm (vertical × horizontal) as set by a focusing mirror and slits. X-ray fluorescence spectra were collected using a continuous scan mode of the monochromator (scan duration ca. 10 min). Up to 6 scans were averaged (1–2 scans per sample spot) for signal-to-noise ratio improvement. XAS data were processed (dead-time correction, background subtraction, normalization) to yield XANES and EXAFS spectra using our earlier described procedures and in-house software^113^. k^3^-weighted EXAFS spectra were simulated with in-house software and phase functions from FEFF9 (*S*_0_^2^ = 0.8)^114^.

### Nuclear resonance vibrational (X-ray) spectroscopy (NRVS)

NRVS data were collected at undulator beamline ID18 at the European Synchrotron Radiation Facility (ESRF, Grenoble, France) using the previously described set-up^115,116^ including a heat-load monochromator, a high-resolution monochromator (FWHM ~ 0.7 meV), gated APD detectors (~1 cm^2^ active area) for prompt and delayed inelastic and forward scattering detection, and a cold-finger liquid-helium cryostat. The storage ring was operated in 16-bunch mode (~90 mA). A sample temperature during the measurements of 30 ± 10 K was estimated from the ratio of NRVS counts in ±2.5–5 meV windows around the resonance. NRVS counts were detected with an APD at 90° to the incident X-ray beam and at ~4 mm distance from the sample. The energy axis of the high-resolution monochromator was calibrated using the CN^−^ vibrational band at 74.0 meV of a (NH_4_)_2_Mg^57^Fe(CN)_6_ powder sample as a reference. NRVS spectra were collected in a −10 meV to 120 meV energy region around the resonance (0.2 meV steps, 2.5 s per data point, spot size on sample ~1.5 × 0.5 mm^2^) and up to 20 scans were averaged for signal-to-noise ratio improvement (5 scans of ~30 min per sample spot). NRVS data were processed, and the normalized partial vibrational density of states (PDOS) was calculated with the software package available at ID18. Energy to frequency axis conversion was done (1 meV = 8.06554 cm^−1^) before comparison of NRVS spectra. For theoretical NRVS spectra from DFT calculations, NRVS vibrational frequencies were derived from normal mode analysis of geometry-optimized, relaxed structures from Gaussian calculations. NRVS and normalized (spectral area = 1) PDOS spectra were calculated using NISspec^117^. Calculated (stick) NRVS spectra were broadened by Lorentzians (FWHM 14 cm^−1^) for comparison with experimental spectra. In-house software and functionalized EXCEL-sheets were used to process Gaussian and NISspec output files. NRVS vibrational modes were visualized with ChemCraft.

### Cyclovoltammetry (CV)

Cyclic Voltammetry was carried out with a CHI 600E potentiostat using a 3 mm diameter glassy carbon disk electrode (ALS Co Ltd.) as working and a platinum wire (length 5 cm, diameter 0.5 mm; ALS Co Ltd.) as counter electrode. Prior to use, electrodes were polished with 0.05 μm alumina suspensions. Ag/AgNO_3_ (10 mM AgNO_3_ and 0.1 M [N(n-Bu)_4_]PF_6_ in MeCN) was used as the reference electrode for room temperature measurements and an Ag wire quasi-reference electrode for low temperature measurements. CV measurements were performed in a three-necked glass cell under an Ar atmosphere. Two milliliters of 1.5 mM compound solution in acetonitrile or acetone was taken in the presence of 100 mM TBAF as the supporting electrolyte. Unless noted otherwise, a scan rate of 100 mV/s was applied. Ferrocene (Fc) was used as an internal reference, and the potential scale is normalized with respect to the potential of the Fc^+/0^ couple.

### Computational methodology

Density functional theory (DFT) calculations were carried out using the Gaussian 16 suite of programs^118^. The spin-unrestricted BP86 functional corrected with Grimme 10’s dispersion^119^ using the Becke–Johnson damping scheme^120^ was employed with two series of basis sets: (a) the TZVP basis set for the iron core and the atoms of the first coordination sphere, and 6–31 G** for the rest atoms. This basis set is denoted as B1 and was used to optimize transition states (TS) and minima; (b) The basis def2-tzvpp basis set for all atoms. This basis set is denoted as B2, which was used for single point energy (SPE) corrections. Solvent effects (acetone, *e* = 20.493) were included in all calculations using the conductor-like polarizable continuum model (CPCM) as implemented in Gaussian 16. An experimental temperature of −90 °C was adopted for the Gibbs free energy calculations.

### Sample preparation

Complex 2 was usually formed by bubbling excess of dioxygen to 2 ml of a 1 mM solution of complex 1 in acetone at −90 °C under inert conditions in a UV–vis quartz cuvette (10 mm pathlength). The formation of 2 was monitored by UV–vis following the bands at 550 and 700 nm. Complex 1-CO was formed by purging an acetone solution with CO in an airtight UV/vis cuvette for 2 min. The solution was then cooled to −90 °C, and 0.05 ml of a 20 mM solution of 1 in acetone was added. The formation of 1-CO occurs immediately, as can be observed from the formation of the band at 604 nm. Complex 3 was usually formed by adding three equivalents of ^S^PhIO dissolved in 0.05 ml DCM (or three equivalents of Bu_4_NIO_4_ dissolved in 0.05 ml acetone) to 2 ml of a 1 mM solution of complex 1 in acetone at −70 °C under inert conditions in a UV–vis quartz cuvette (10 mm pathlength). The same procedure was followed with 3-d_6_ using acetone-d_6_ as solvent. The formation of 3 was monitored by UV–vis following the bands at 525 and 810 nm. The samples were transferred into precooled sample holders as soon as the formation was complete. For the preparation of GC samples, five equivalents of substrate were added. After the 2000 s, the reaction solution was quenched with NaHSO_3_ and biphenyl was added as an internal standard. The solution was filtered with a small column filled with silica and MgSO_4_, rinsing with EtOAc. From the obtained solutions, 1 ml of each was used for GC-analysis.

### Generation of ^16/18^O_2_

In a 500 ml Young Schlenk flask, 100 mbar of ^16^O_2_ and ^18^O_2_ were added by using a 3-way stopcock. The pressure was then reduced to approx. 70 mbar by carefully applying vacuum (note that low pressure is necessary, otherwise the applied energy in the next step will not lead to the breaking of the oxygen–oxygen bonds, but mainly to kinetic collisions). Then a High Frequency Generator (model BD-10ASV from Electro-Technic Products 4642 N Ravenswood Ave Chicago, IL 60640 USA) was used to apply high voltage (45 kV at a frequency of approx. 500 kHz with a current output about 1 mA) to the Schlenk flask (4 × 10 min with 20-min breaks in between to avoid overheating the generator), causing sparks to be observed. The gas mixture was then condensed into a 10 ml Young Schlenk flask containing a degassed, frozen solution of complex 1/1-OTf in acetone-d_6_ for intermediate formation. Intermediate formation took place at −90 °C using a cooling bath.

### Band fitting analysis of the rRaman spectra

The band fitting analysis was carried out on the basis of Lorentzian functions. First, a weak solvent band at 818.7 cm^−1^ (half width 24.2 cm^−1^), determined from the spectrum of the neat solvent, was subtracted from the spectral region of interest (785-840 cm^−1^) of the complexes. Subsequently, the spectrum of complex 2 generated with ^16^O_2_, was analyzed to yield a band at 823.4 cm^−1^ (half width 12.4 cm^−1^). The spectrum of the complex generated by the ^16^O_2_/^18^O_2_ gas mixture was then simulated by four Lorentzians. Here we fixed the band widths to 12.4 cm^−1^ as determined for the ^16^O_2_-complex (Supplementary Fig. 20A). The fit provides an excellent reproduction of the bands of ^16^O_2_- and ^18^O_2_-complexes and also yields two band components at positions expected for the mixed ^16^O–^18^O-complexes. The residuals (Supplementary Fig. 20B) correspond to the noise level underlining the high quality of the fit. Note that the experimental set up does not ensure a gas supply at 1:1 ratio of ^16^O_2_ and ^18^O_2_, which accounts for deviations from the corresponding intensity ratio of 1:1:1:1. The alternative fit using three Lorentzian functions (Supplementary Fig. 20C) yielded similar residuals (Supplementary Fig. 20D) and thus a mathematically equally good fit. However, it does not reproduce the bands positions of ^16^O_2_- and ^18^O_2_-complexes, such that the side-on configuration can be ruled out.

### Synthesis of tert-butylphenylsulfide

The compound has been previously reported^121,122^, and was independently synthesized as described below. A large excess of isobutene was condensed into a rapidly stirred suspension of Amberlyst H-15 (3 g) in thiophenol (11 g, 0.1 mol) and diethylether (80 mL) at –78 °C. The suspension was stirred overnight at –78 °C (dry ice bath). Upon completion, the mixture was allowed to warm to room temperature and subsequently filtered. The solvent was removed under reduced pressure to yield tert-butylphenylsulfide as colorless liquid (16.3 g, 95%) with a strong odor. ^1^H-NMR (300 MHz; CDCl_3_): δ = 7.47 (m, 2H), 7.28 (m, 2H), 7.20 (m, 1H), 1.22 (s, 9H) ppm.

### Synthesis of tert-butylphenylsulfone

The compound has been previously reported^121,122^, and was independently synthesized as described below. Sodium perborate tetrahydrate (20.8 g, 120 mmol) was added to a solution of tert-butylphenylsulfide (5 g, 30 mmol) in acetic acid (60 mL). The cloudy suspension was stirred at 50 °C for 3 h, cooled, poured into water (200 mL), and extracted with dichloromethane (4 × 50 mL). The combined extracts were washed repeatedly with aqueous sodium hydrogen carbonate and water until the acid had been neutralized, and then dried (MgSO_4_) and evaporated under reduced pressure. The residue was washed with pentane to yield tert-butylphenylsulfone (2.26 g, 38%) as a colorless solid. ^1^H-NMR (300 MHz; CDCl_3_): δ = 7.94–7.86 (m, 2H), 7.68–7.53 (m, 3H), and 1.34 (s, 9H) ppm.

### Synthesis of tert-butylsulfonyliodobenzene

The compound has been previously reported^121,123^, and was independently synthesized as described below. To tert-butylphenylsulfone (8.74 g, 44 mmol) in 100 mL THF at –78 °C was added 21.12 mL of n-BuLi in hexane (2.5 M, 52.8 mmol) over 5 min via syringe. After stirring for 40 min, a solution of I_2_ (17.5 g, 52.8 mmol) in 50 mL THF was added by cannula to the orange solution. The reaction mixture was stirred vigorously and allowed to warm up to room temperature. The excess iodine was quenched with aqueous sodium sulfite (Na_2_SO_3_). The organic layer was then separated and combined with another extraction of the aqueous layer with diethylether. The combined organic extracts were dried (MgSO_4_) and evaporated under vacuum to give tert-butylsulfonyliodobenzene (10.80 g, 75%) as a yellow solid. ^1^H-NMR (CDCl_3_, 300 MHz) δ = 8.21–8.14 (m, 1H), 8.12–8.06 (m, 1H), 7.60–7.52 (m, 1H), 7.24–7.18 (m, 1H), 1.42 (s, 9H) ppm.

### Synthesis of 2-(tert-butylsulfonyl)(diacetoxyiodo)benzene

The compound has been previously reported^121,123^, and was independently synthesized as described below. A mixture of hydrogen peroxide (5 ml, 30%) and acetic anhydride (21 ml, 222 mmol) was stirred at 40 °C for 4 h. The solution was cooled to room temperature. To this solution was added tert-butylsulfonyliodobenzene (5 g, 15.4 mmol). The resulting pale-yellow solution was stirred for 24 h at 25–30 °C (if temperature is allowed to increase above this range PhSO_2_^t^Bu is formed). The solvent was removed under reduced pressure to yield 2-(tert-butylsulfonyl)(diacetoxyiodo)benzene as a white solid (2.07 g, 33%). The product is best used directly for the synthesis of ^S^PhIO, but can be further purified, albeit in greatly reduced yields, by recrystallization from a cyclohexane/acetic acid mixture. ^1^H NMR (CDCl_3_, 300 MHz): δ = 8.58–8.55 (m, 1H), 8.29–8.26 (m, 1H), 7.87–7.82 (m, 1H), 7.72–7.67 (m, 1H), 1.95 (s, 6H), 1.42 (s, 9H) ppm.

### Synthesis of 2-(tert-butylsulfonyl)iodosylbenzene (^S^PhIO)

The compound has been previously reported^121,123^, and was independently synthesized as described below. To solid 2-(tert-butylsulfonyl)(diacetoxyiodo)benzene (2.4 g, 5.42 mmol) was added an aqueous solution of sodium hydroxide (20 ml, 3 M) dropwise with vigorous stirring. The resulting yellow suspension was stirred for another 25 min. The yellow solid was collected by filtration, washed with water and diethyl ether, and then dried under reduced pressure to give 2-(tert-butylsulfonyl)iodosylbenzene (300 mg, 16%) as a yellow powder. ^1^H-NMR (CDCl_3_, 300 MHz): δ = 8.14–8.06 (m, 1H), 7.94–7.83 (m, 2H), 7.69–7.60 (m, 1H), 1.42 (s, 9H) ppm. ^S^PhI^18^O was synthesized analogously using Na^18^OH, prepared from NaH and H_2_^18^O.

### Synthesis of Fe(MeCN)_2_(OTf)_2_

In a Schlenk flask, 6 ml of concentrated hydrochloric acid (37%) was degassed by bubbling argon through the solution (note: do not use a metal cannula for this step). Elemental iron (100 mg) was weighed using a nonmagnetic spatula, then collected with a magnetic stir bar and added to the acid solution under an inert atmosphere. Immediate gas evolution is observed. The closed reaction mixture was heated to 60 °C and stirred until a clear, colorless solution was obtained. To prevent overpressure from building up, the pressure was occasionally reduced slightly. Drying at 60 °C under vacuum yielded FeCl_2_·4H_2_O as a white solid, which was then suspended in dry acetonitrile (10 ml). An excess of (CH_3_)_3_SiOTf (4 g) was added slowly in a glovebox. The solution was stirred for 18 h, and the solvent was removed by vacuum. The remaining yellow solid was dissolved in a minimum amount of acetonitrile, and an excess of dry diethyl ether was added. The mixture was shaken vigorously during which time white precipitate was formed. The solvent was then removed by decantation. The remaining solid was washed five times with dry diethyl ether and dried, yielding [Fe(MeCN)_2_(OTf)_2_] as a white solid (700 mg, 90%). Mössbauer spectroscopy revealed a single iron species with δ = 1.36 mm/s and Δ*E*_Q_ = 3.30 mm/s. ^57^Fe(MeCN)_2_(OTf)_2_ was synthesized analogously using elemental ^57^Fe.

### Synthesis of [Fe(TMG_3_tren)(OTf)]OTf

A solution of TMG_3_tren (120 mg, 0.27 mmol) in 1 ml acetonitrile was added dropwise to a solution of Fe(MeCN)_2_(OTf)_2_ (110 mg, 0.25 mmol) in 1.5 ml acetonitrile. After stirring for 24 h at room temperature, the solution was dropped into 20 ml of diethyl ether, resulting in the precipitation of a white solid. The solvent was then removed, the solid was washed twice with diethyl ether and dried to yield [Fe(TMG_3_tren)(OTf)](OTf) as a white solid (145 mg, 72%). Mössbauer spectroscopy revealed a single iron species with δ = 1.00 mm/s and Δ*E*_Q_ = 1.66 mm/s.

### Synthesis of Fe(THF)_5_(NTf_2_)_2_

In a Schlenk flask, HNTf_2_ (1 g, 3.55 mmol) was dissolved in dry benzene (6 ml), cooled to 0 °C, and PhSiMe_3_ (0.51 ml, 3 mmol) was added dropwise. The reaction mixture was stirred for 1 h while warming to room temperature. The resulting solution was then transferred to a suspension of FeCl_2_ (172 mg, 1.36 mmol) in THF (6 ml), which turned yellow upon addition. After stirring for 16 h, the supernatant was removed by pipette, and the precipitate washed five times with THF and twice with hexane. Subsequent drying yielded Fe(THF)_5_(NTf_2_)_2_ (1.10 g, 83%) as a white solid. Mössbauer spectroscopy revealed a single iron species with δ = 1.39 mm/s and Δ*E*_Q_ = 3.01 mm/s.

### Synthesis of tris(N’,N”-diisopropylguanidinyl-2-ethyl)amine (DIG_3_tren)

The synthesis is based on a literature procedure^70^. A mixture of tris(2-aminoethyl)amine (0.3 ml, 2 mmol) and diisopropylcarbodiimide (DIC, 1 ml, 6.46 mmol) was heated under argon to 100 °C for 3 h The excess of DIC was removed under vacuum, yielding DIG_3_tren as a viscous, pale yellow oil (997 mg, 95%). ^1^H-NMR (500 MHz, CDCl_3_) δ = 3.53 (br, 6H), 3.10 (t, *J* = 6.48 Hz, 6H), 2.69 (t, *J* = 6.48 Hz, 6H), 1.11 (d, *J* = 6.33 Hz, 36H) ppm. ESI-MS (pos. mode): *m/z* 525.4 [(DIG_3_tren+H)^+^].

### Synthesis of [Fe(DIG_3_tren)](OTf)_2_ (1/1-OTf)

A solution of DIG_3_tren (255 mg, 0.48 mmol) in 1.5 ml acetonitrile was added dropwise to a solution of Fe(MeCN)_2_(OTf)_2_ (159 mg, 0.36 mmol) in 1.5 ml acetonitrile. After stirring for 20 h at room temperature, the yellow solution was dropped into 20 ml of diethyl ether, resulting in the precipitation of a light-yellow solid. The solvent was then removed, the solid was washed twice with diethyl ether and dried to yield [Fe(DIG_3_tren)](OTf)_2_ as a light-yellow solid (172 mg, 55%). Colorless crystals suitable for X-ray crystallography were obtained from an acetone/diethyl ether solution in the ratio (1:1). For Mössbauer and NRVS samples, [^57^Fe(DIG_3_tren)](OTf)_2_ was synthesized starting from ^57^Fe(MeCN)_2_(OTf)_2_. ^19^F-NMR (282 MHz, acetone-d_6_) δ = −78.86 (s) ppm. ESI-MS (pos. mode): *m/z* 729.3 [Fe(DIG_3_tren)(OTf)]^+^, 290.2 [Fe(DIG_3_tren)]^2+^. Elemental analysis calc. for C_29_H_60_F_6_FeN_10_O_6_S_2_: C 39.63, H 6.88, N 15.94, S 7.30; found: C 39.82, H 6.98, N 15.92, S 7.07.

### Synthesis of [Fe(MeCN)_6_](BPh_4_)_2_

A solution of NaBPh_4_ (706 mg, 2.06 mmol) in 5 ml acetonitrile was added dropwise to a solution of Fe(MeCN)_2_(OTf)_2_ (300 mg, 0.69 mmol) in 5 ml acetonitrile. A white solid precipitated immediately. After stirring for 2 h, the solid was filtered off, washed twice with acetonitrile and diethyl ether and dried to yield [Fe(MeCN)_6_](BPh_4_)_2_ as a white solid (380 mg, 58%). Mössbauer spectroscopy revealed a single iron species with δ = 1.28 mm/s and Δ*E*_Q_ = 0.78 mm/s.

### Synthesis of [Fe(DIG_3_tren)](BPh_4_)_2_ (1-BPh_4_)

A solution of DIG_3_tren (150 mg, 0.28 mmol) in 2 ml acetonitrile was added dropwise to a solution of [Fe(MeCN)_6_](BPh_4_)_2_ (220 mg, 0.23 mmol) in 1.5 ml acetonitrile. After stirring for 24 h at room temperature, the yellow solution was dropped into 20 ml of diethyl ether, resulting in the precipitation of a pale yellow solid. The solvent was then removed, the solid was washed twice with diethyl ether and dried to yield [Fe(DIG_3_tren)](BPh_4_)_2_ as a pale yellow solid (160 mg, 57%). Mössbauer spectroscopy revealed two iron species with δ = 1.00 mm/s and Δ*E*_Q_ = 2.62 mm/s and δ = 0.85 mm/s and Δ*E*_Q_ = 1.39 mm/s. Elemental analysis calc. for C_75_H_100_B_2_FeN_10_: C 73.89, H 8.27, N 11.49; found: C 74.18, H 8.29, N 11.45.

### Synthesis of [Fe(DIG_3_tren)](BF_4_)_2_ (1-BF_4_)

A solution of DIG_3_tren (500 mg, 0.95 mmol) in 1.5 mL MeCN was added dropwise to a suspension of Fe(BF_4_)_2_·6H_2_O (290 mg, 0.86 mmol) in 2 mL MeCN. The reaction mixture was stirred for 20 h at room temperature. The resulting dark green mixture was precipitated in 20 mL diethyl ether. The solvent was removed, and the precipitate was washed two times with diethyl ether and dried to yield [Fe(DIG_3_tren)](BF_4_)_2_ as a dark green solid (350 mg, 55%). Mössbauer spectroscopy revealed a single iron species with δ = 1.28 mm/s and Δ*E*_Q_ = 3.01 mm/s. Elemental analysis calc. for C_27_H_60_B_2_F_8_FeN_10_: C 42.99, H 8.02, N 18.57; found: C 43.12, H 8.04, N 18.51.

### Synthesis of [Fe(DIG_3_tren)](NTf_2_)_2_ (1-NTf_2_)

A solution of DIG_3_tren (140 mg, 0.26 mmol) in 1 ml acetonitrile was added dropwise to a solution of [Fe(THF)_5_](NTf_2_)_2_ (235 mg, 0.24 mmol) in 1.5 ml acetonitrile. After stirring for 24 h at room temperature, the yellow solution was dropped into 20 ml of diethyl ether, resulting in the precipitation of a pale yellow solid. The solvent was then removed, the solid was washed twice with diethyl ether and dried to yield [Fe(DIG_3_tren)](NTf_2_)_2_ as a pale yellow solid (190 mg, 69%). Mössbauer spectroscopy revealed three iron species with δ = 1.03 mm/s and Δ*E*_Q_ = 2.68 mm/s, δ = 0.89 mm/s and Δ*E*_Q_ = 1.92, δ = 0.84 mm/s and Δ*E*_Q_ = 1.06 mm/s, mm/s. Elemental analysis calc. for C_31_H_60_F_12_FeN_12_O_8_S_4_: C 32.63, H 5.30, N 14.73, S 11.24; found: C 32.75, H 5.32, N 14.69, S 11.28.

### Synthesis of [Zn(DIG_3_tren)(Br)]Br (Zn-Br)

A solution of DIG_3_tren (730 mg, 1.39 mmol) in 1.5 mL MeCN was added dropwise to a suspension of ZnBr_2_ (280 mg, 1.25 mmol) in 2 mL MeCN. The reaction mixture was stirred for 20 h at room temperature. The resulting white suspension was precipitated in 20 mL diethyl ether. To ensure complete precipitation, the solution was stored at −40 °C for 20 h. The solvent was removed, and the precipitate was washed two times with diethyl ether and dried to yield [Zn(DIG_3_tren)Br]Br as a white solid (580 mg, 60%). Colorless crystals suitable for X-ray crystallography were obtained by vapor diffusion from a MeCN solution with diethyl ether. Elemental analysis calc. for C_27_H_60_Br_2_N_10_Zn: C 43.24, H 8.06, N 18.68; found: C 43.40, H 8.09, N 18.62.

### Synthesis of tren-d_6_, DIG_3_tren-d_6_ and [Fe(DIG_3_tren-d_6_)](OTf)_2_ (1-d_6_)

Tris(2-aminoethyl)amine (0.6 ml, 4 mmol) was mixed with 1.3 ml D_2_O, stirred for 24 h, and then dried. This step was repeated to ensure complete deuteration to form tris(2-aminoethyl)amine-d_6_. ^1^H-NMR (300 MHz, CDCl_3_) δ = 2.66 (t, *J* = 6.12 Hz, 6H), 2.42 (t, *J* = 6.12 Hz, 6H) ppm. Subsequently, a mixture of tris(2-aminoethyl)amine-d_6_ (0.3 ml, 2 mmol) and diisopropylcarbodiimide (DIC, 0.93 ml, 6 mmol) was heated under argon to 100 °C for 3 h. Drying under vacuum yielded DIG_3_tren-d_6_ as a viscous, pale yellow oil. 1-d_6_ was synthesized by replacing DIG_3_tren and acetonitrile with DIG_3_tren-d_6_ and acetonitrile-d_3_ following the procedure for 1.

## Supplementary information

Supplementary Information

Transparent Peer Review File

## Source data

Source Data

## Data Availability

The authors declare that the data supporting the findings of this study are available within the paper and its Supplementary Information files. Should any raw data files be needed in another format, they are available from the corresponding author upon request. The optimized Cartesian coordinates generated in this study are provided in the Source data file. The X-ray crystallographic coordinates for structures reported in this study have been deposited at the Cambridge Crystallographic Data Centre (CCDC), under deposition numbers 2477483 (1) and 2477484 (Zn-Br). These data can be obtained free of charge from The Cambridge Crystallographic Data Centre via www.ccdc.cam.ac.uk/data_request/cif. Source data are provided with this paper.
