# Supplementary material for: A mononuclear nonheme iron complex with higher affinity for O2 than CO via hydrogen bonding
Source: Nat Commun. 2026 May 5;17:6101. doi: 10.1038/s41467-026-72599-y (PMC13358151; doi:10.1038/s41467-026-72599-y)
Supplement: Supplementary file 1 — Supplementary Information [file 41467_2026_72599_MOESM1_ESM.pdf]

Supporting Information for

A Mononuclear Non-heme Iron(II) Complex Exhibiting H-Bonding Mediated Higher Affinity for O_2 than for CO

Matthias Jux [1], Stefan Mebs [2], Michael Haumann [2], Sagie Katz [3], Ricardo Garcia-Serres [4], Peter Hildebrandt [3], Yong Wang [5], Wonwoo Nam [6]*, Kallol Ray [1]*

[1]Department of Chemistry, Humboldt-Universität zu Berlin, Berlin, Germany. [2]Department of Physics, Freie Universität Berlin, Berlin, Germany. [3]Department of Chemistry, Technische Universität Berlin, Berlin, Germany. [4]Laboratoire Chimie et Biologie des Métaux, Grenoble, France. [5]Institute of Drug Discovery Technology, Ningbo University, Ningbo 315211, China. [6]Department of Chemistry and Nano Science, Ewha Womans University, Seoul, South Korea.

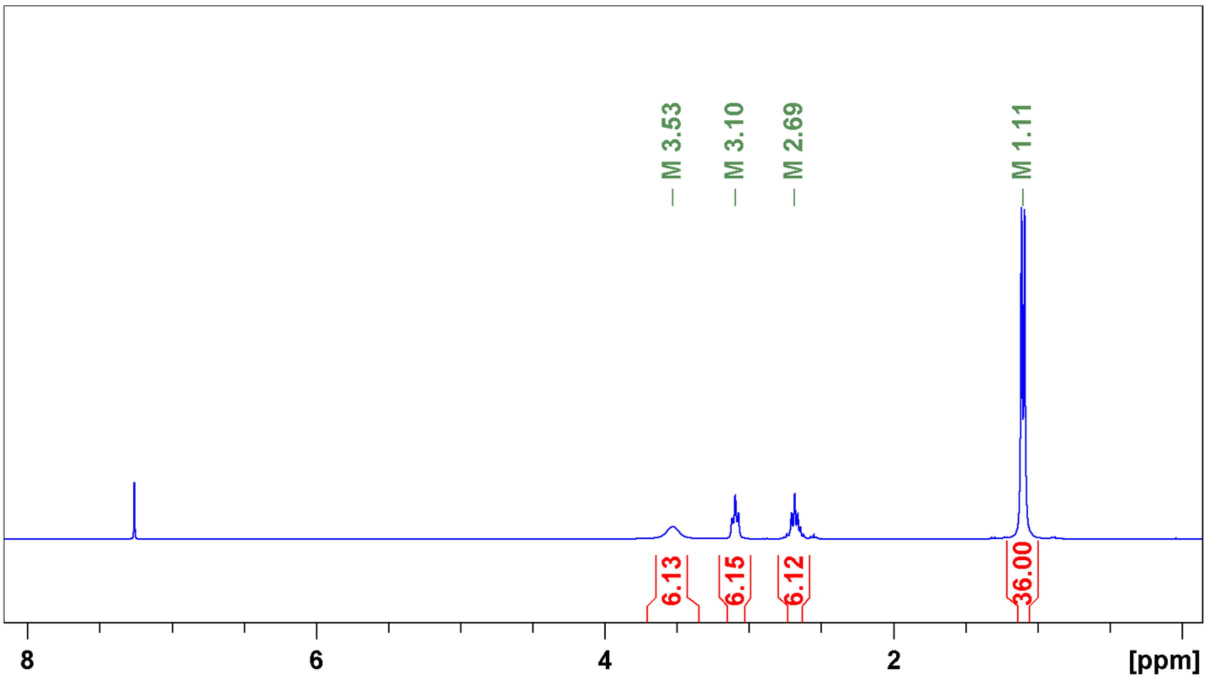

Supplementary Figure 1. ^{1}H-NMR spectrum of DIG$_3$tren in $CDCl_3$ (300 MHz).

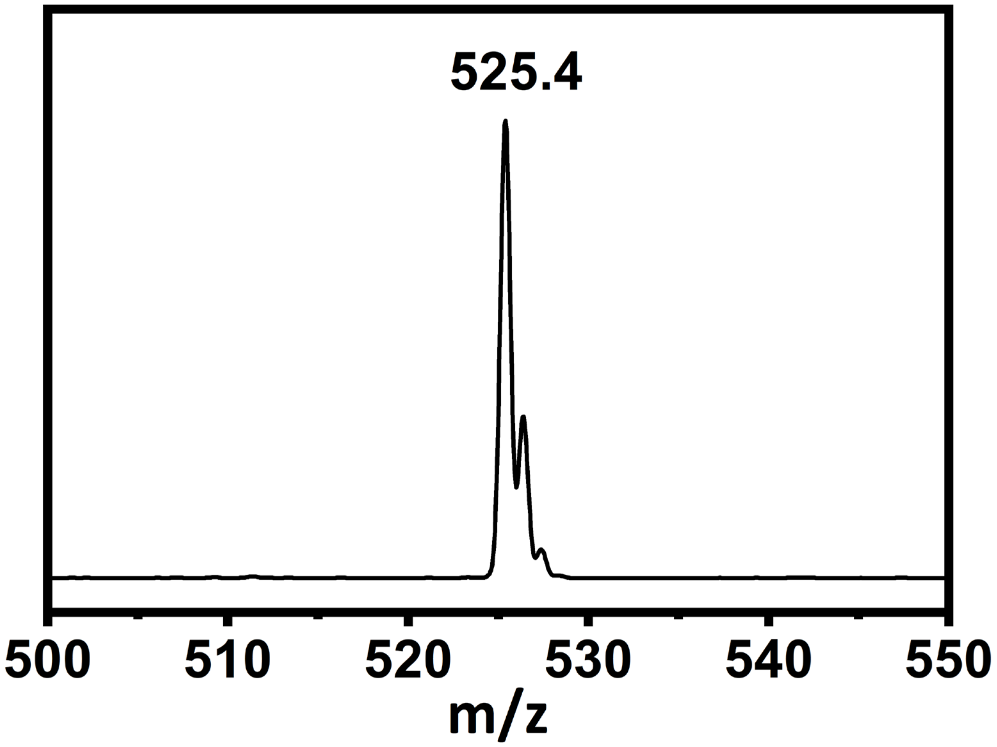

Supplementary Figure 2. ESI-MS spectrum of DIG$_3$tren showing the peak assigned to [(DIG$_3$tren+H)$^+$].

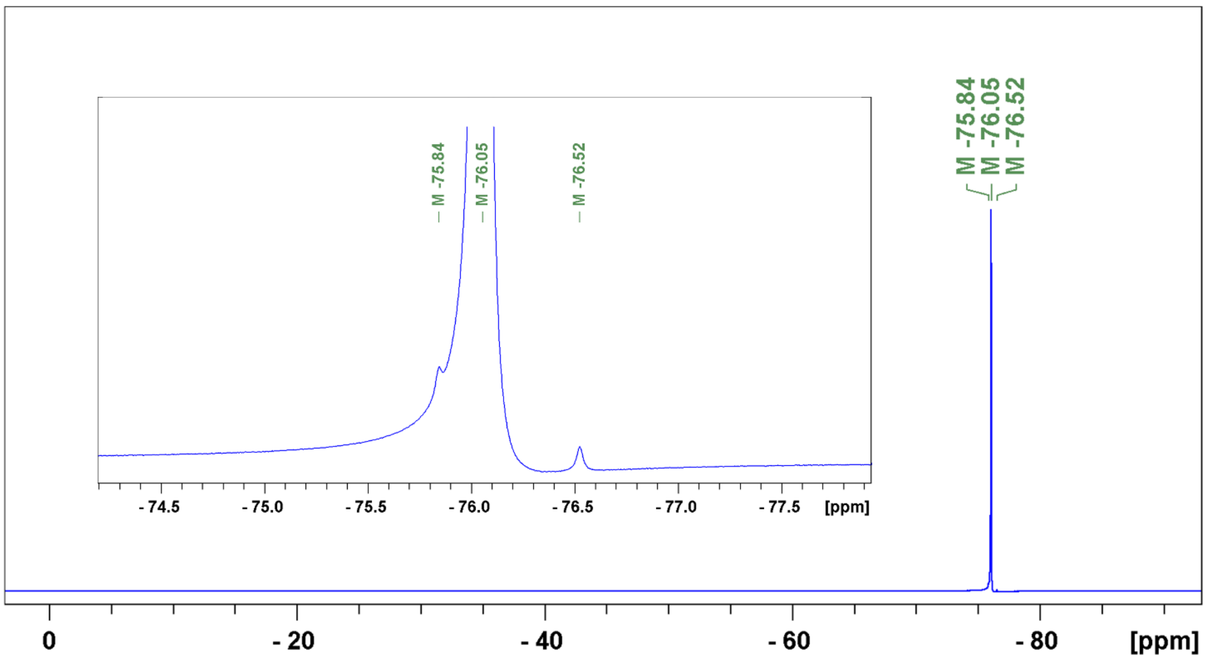

Supplementary Figure 3. ^{19}F-NMR spectrum of **1** in acetone-d_6 (282 MHz) with zoom as inset.

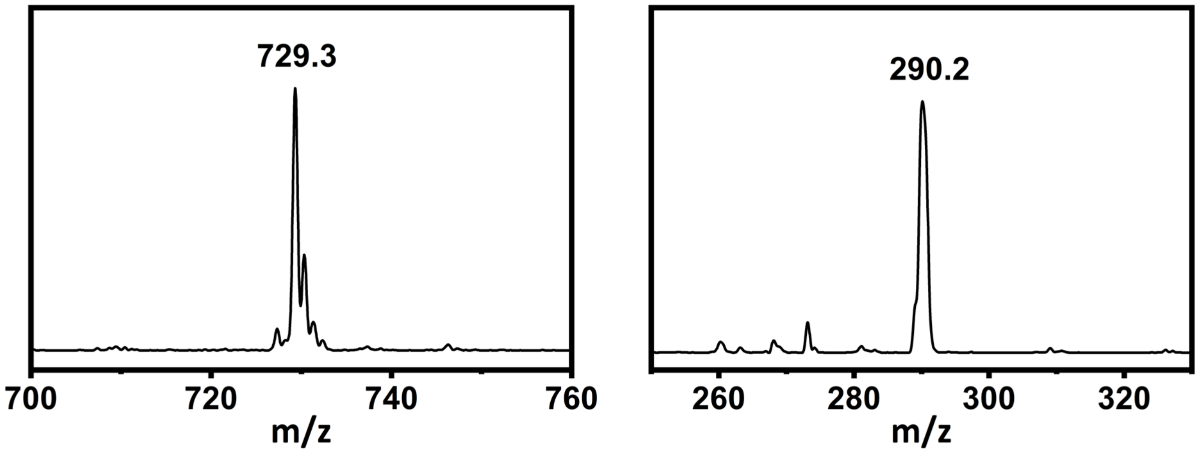

Supplementary Figure 4. ESI-MS spectrum of **1** showing the peaks assigned to [[Fe(DIG$_3$tren)(OTf)]$^+$] left and [[Fe(DIG$_3$tren)]$^{2+}$] right.

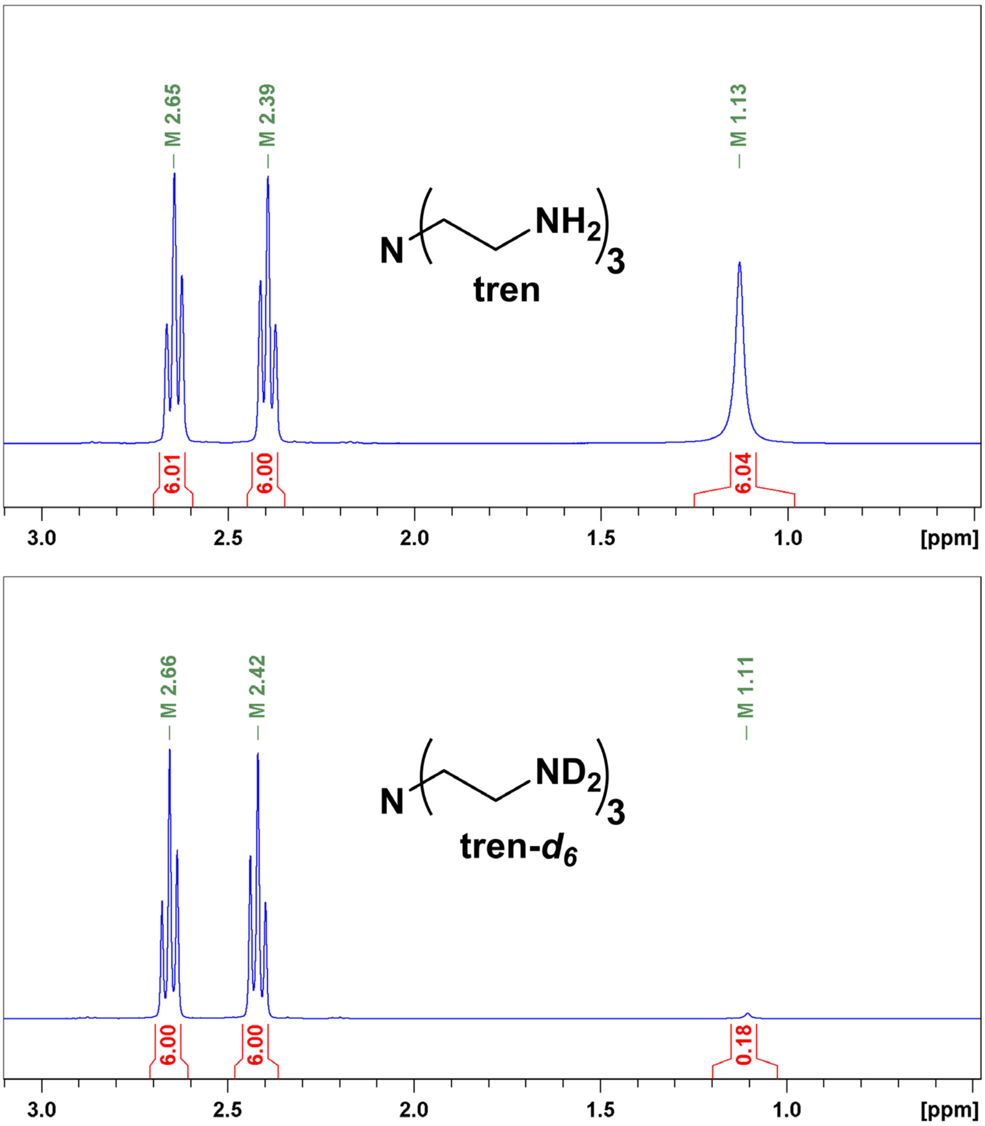

Supplementary Figure 5. Top: ^{1}H-NMR spectrum of tren in $CDCl_3$ (300 MHz), bottom: ^{1}H-NMR spectrum of tren-d_6 in $CDCl_3$ (300 MHz).

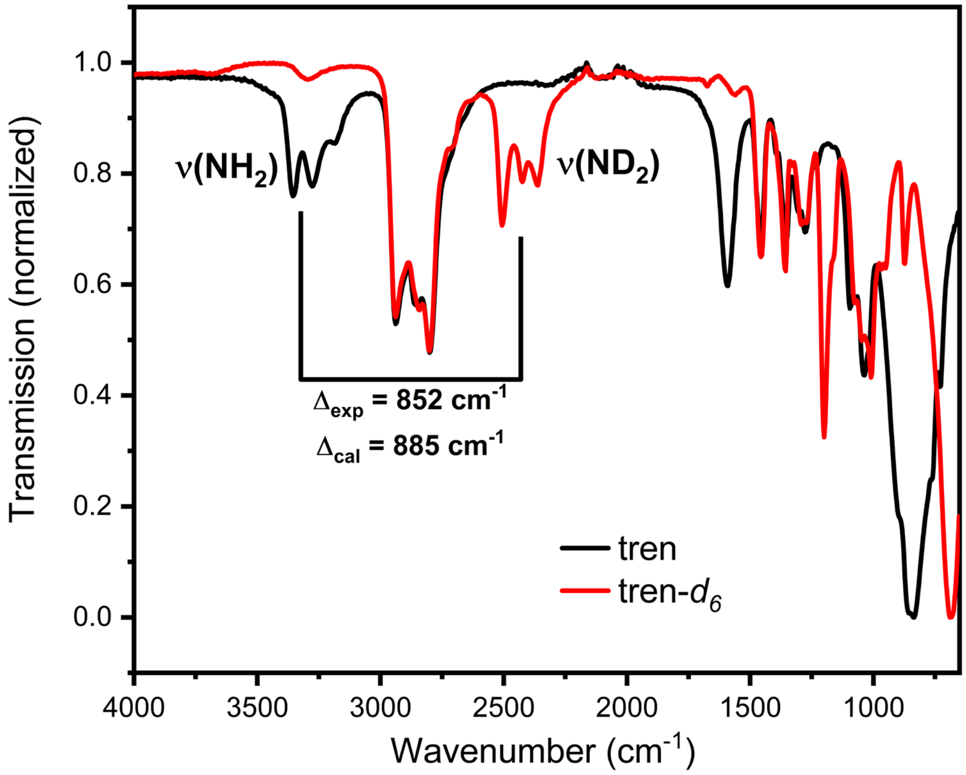

Supplementary Figure 6. FTIR spectra of tren and tren-d_6.

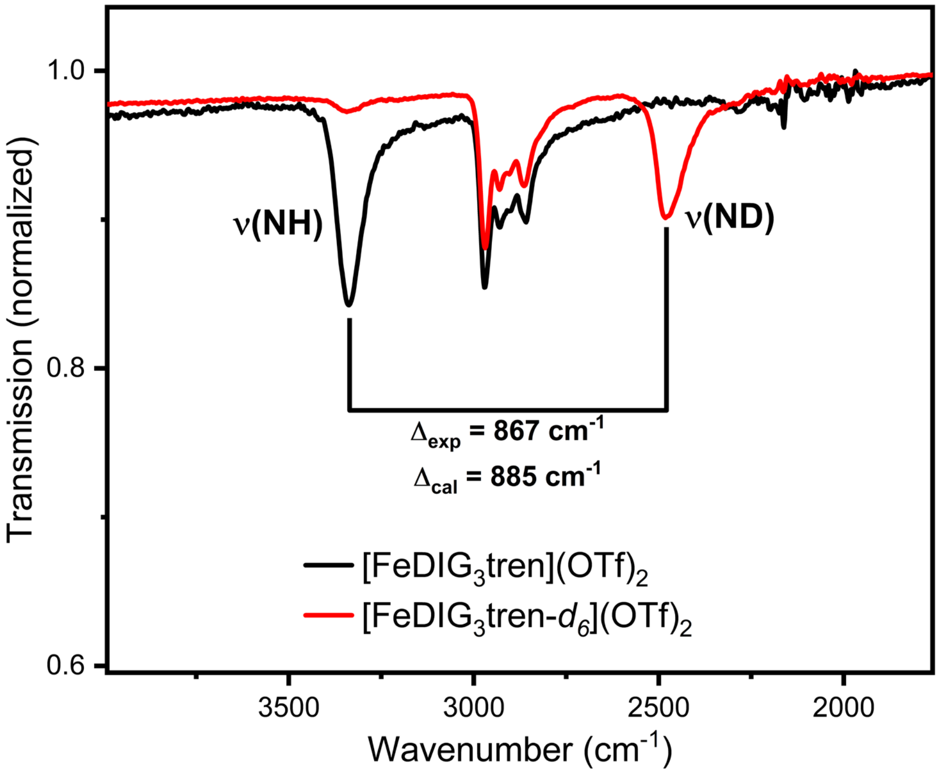

Supplementary Figure 7. FTIR spectra of $[FeDIG_3tren](OTf)_2$ and $[FeDIG_3tren\text{-}d_6](OTf)_2$.

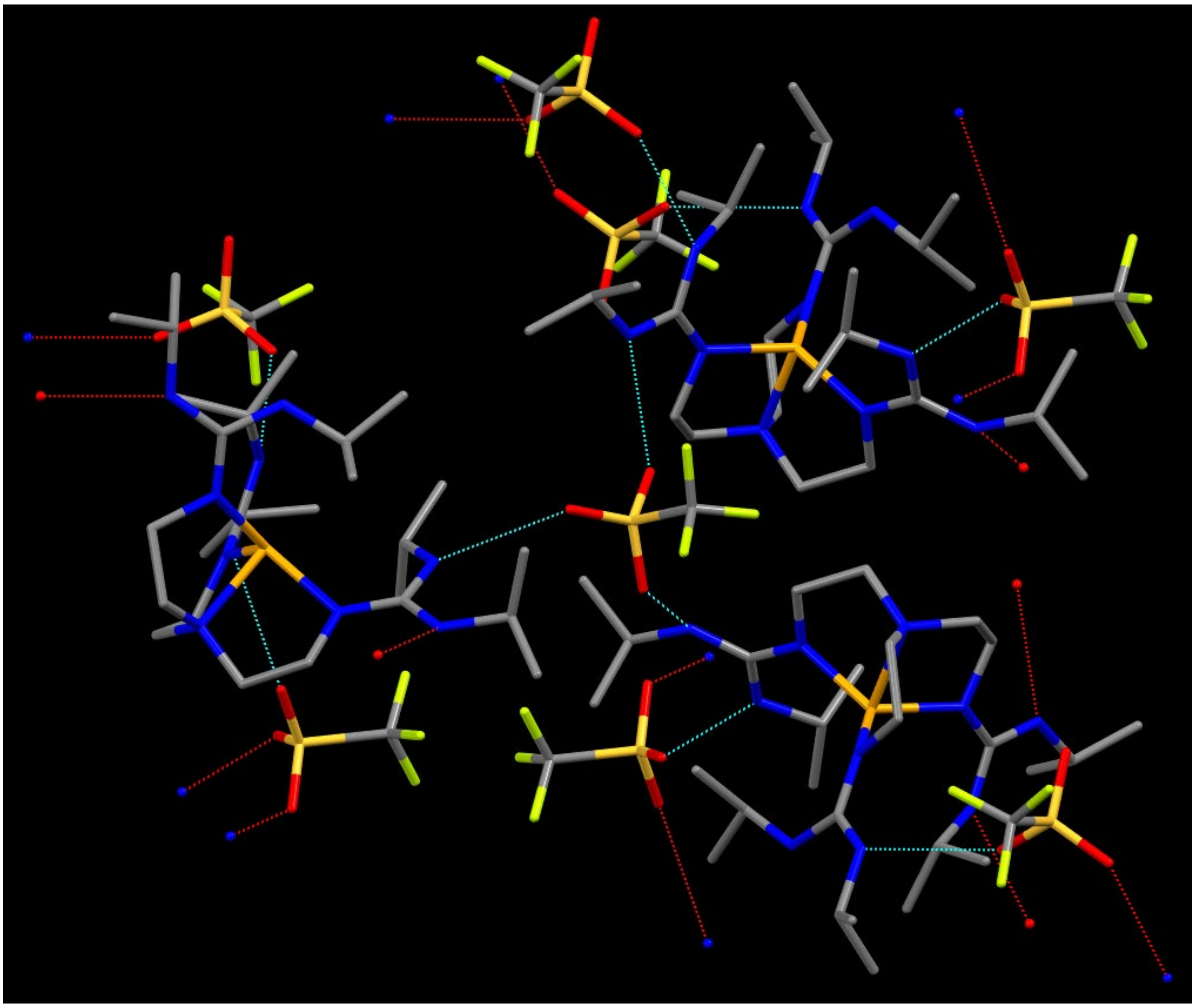

Supplementary Figure 8. Solid-state packing diagram illustrating the hydrogen-bonding network and short intermolecular contacts in the crystal structure of **1**. The triflate anions engage in multiple N–H···O hydrogen bonds (cyan dashed lines) with the ligand scaffold, forming an extended secondary-sphere interaction network. Additional close contacts involving triflate oxygen atoms are shown as red dashed lines. Hydrogen atoms are omitted for clarity. Color code: Fe, orange; N, blue; O, red; S, yellow; C, gray; F, green.

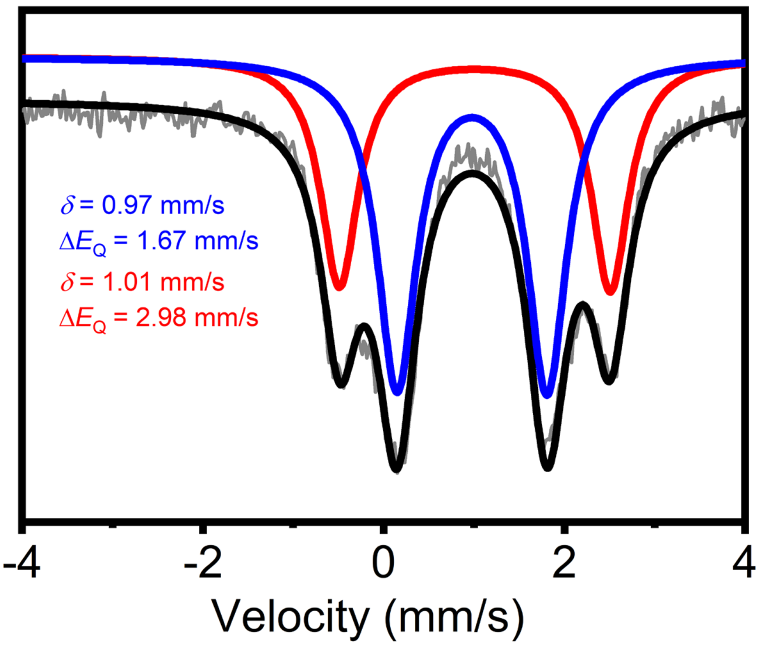

Supplementary Figure 9. Zero-field Mössbauer spectrum of solid **1/1-OTf** at 14 K (grey line: experimental spectrum; black line: simulated spectrum; blue line: **1-OTf** with δ = 0.97 mm/s, ΔE_Q = 1.67 mm/s, Γ = 0.50 mm/s, 68%; red line: **1** with δ = 1.01 mm/s, ΔE_Q = 2.98 mm/s, Γ = 0.50 mm/s, 32%).

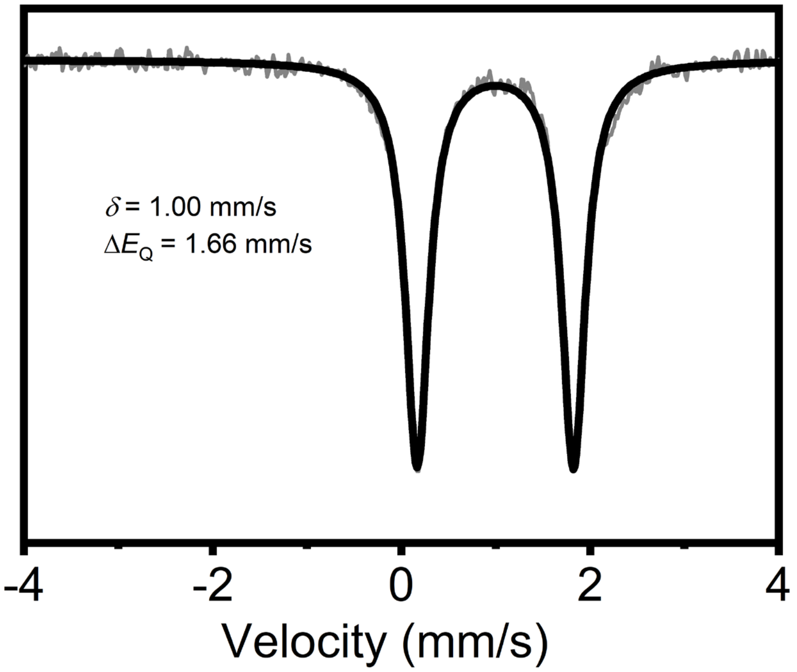

Supplementary Figure 10. Zero-field Mössbauer spectrum of solid [Fe(TMG$_3$tren)(OTf)](OTf) at 14 K (grey line: experimental spectrum; black line: simulated spectrum with δ = 1.00 mm/s, ΔE_Q = 1.66 mm/s).

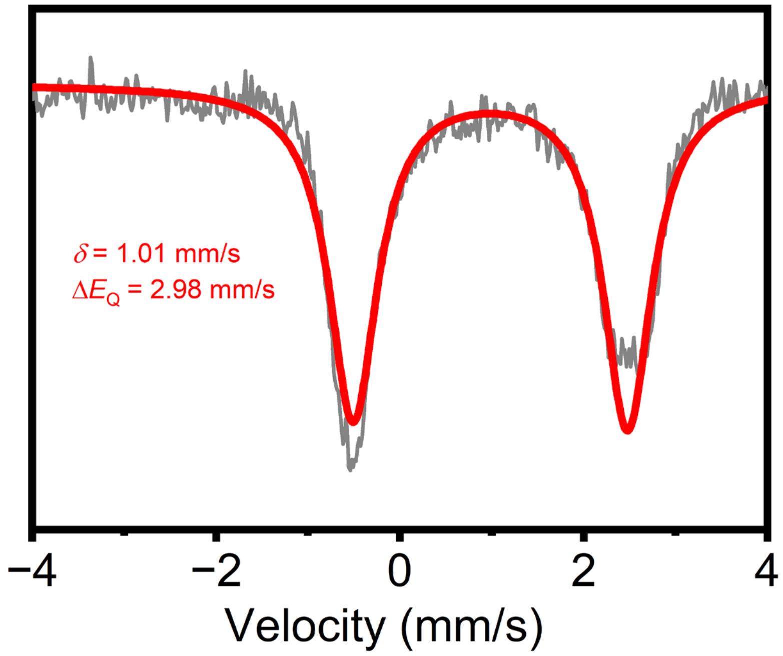

Supplementary Figure 11. Zero-field Mössbauer spectrum of crystalline **1** at 14 K (grey line: experimental spectrum; red line: simulated spectrum with δ = 1.01 mm/s, ΔE_Q = 2.98 mm/s, Γ = 0.64 mm/s).

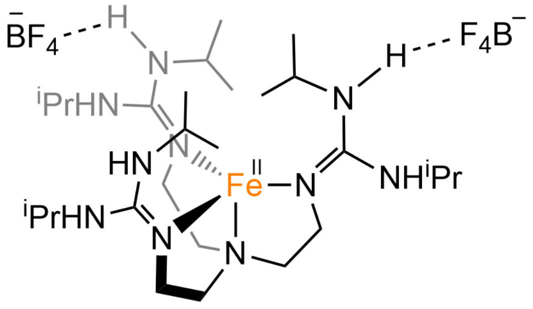

1-BF$_4$

Supplementary Figure 12. Schematic representation of **1-BF$_4$**.

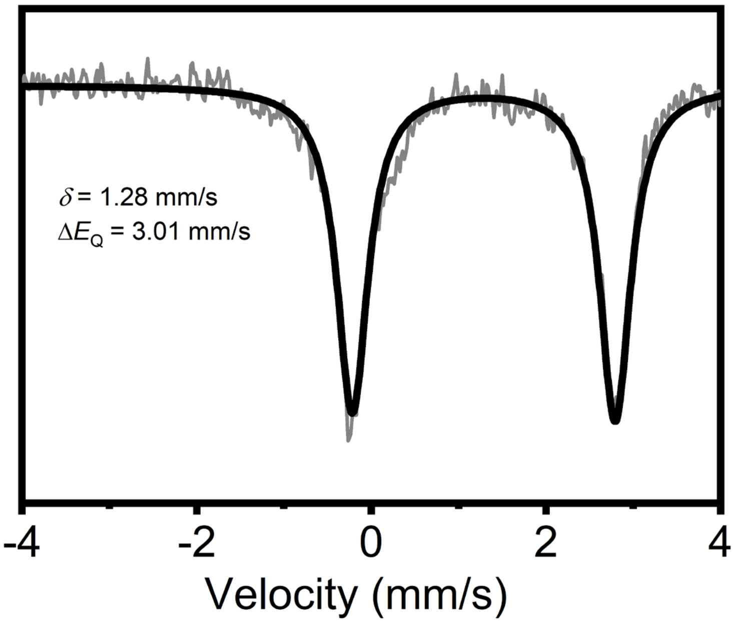

Supplementary Figure 13. Zero-field Mössbauer spectrum of **1-BF$_4$** at 14 K (grey line: experimental spectrum; black line: simulated spectrum with δ = 1.28 mm/s, ΔE_Q = 3.01 mm/s).

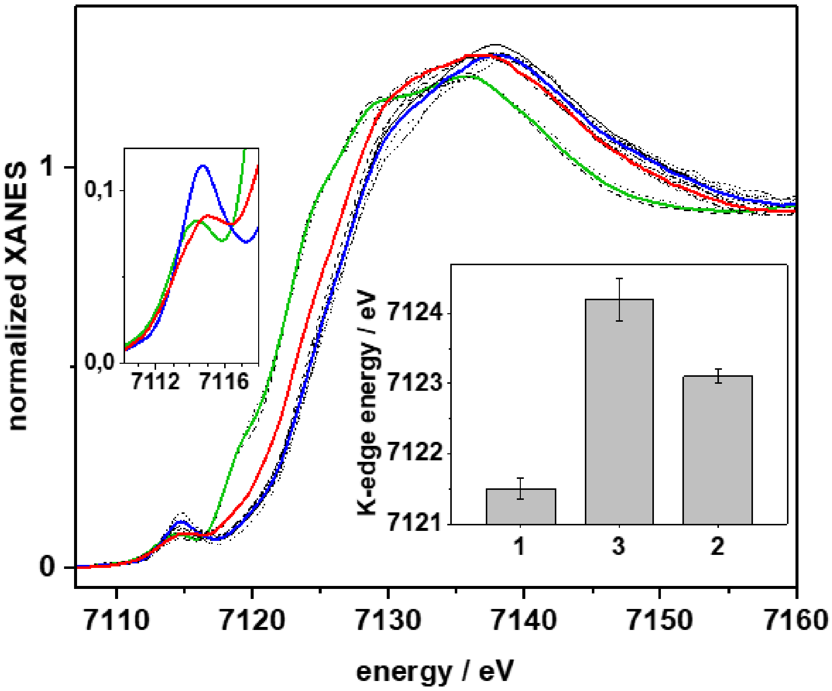

Supplementary Figure 14. XANES spectra of indicated samples (black thin lines, individual spectra of different (two to six) samples of the three types: **1** green line, **3** blue line, **2** red line; colored lines show the respective mean spectra). The insets show the respective mean pre-edge spectra in magnification (left) and the K-edge energies (right; at 50 % level, error bars represent the full range of the individual spectra).

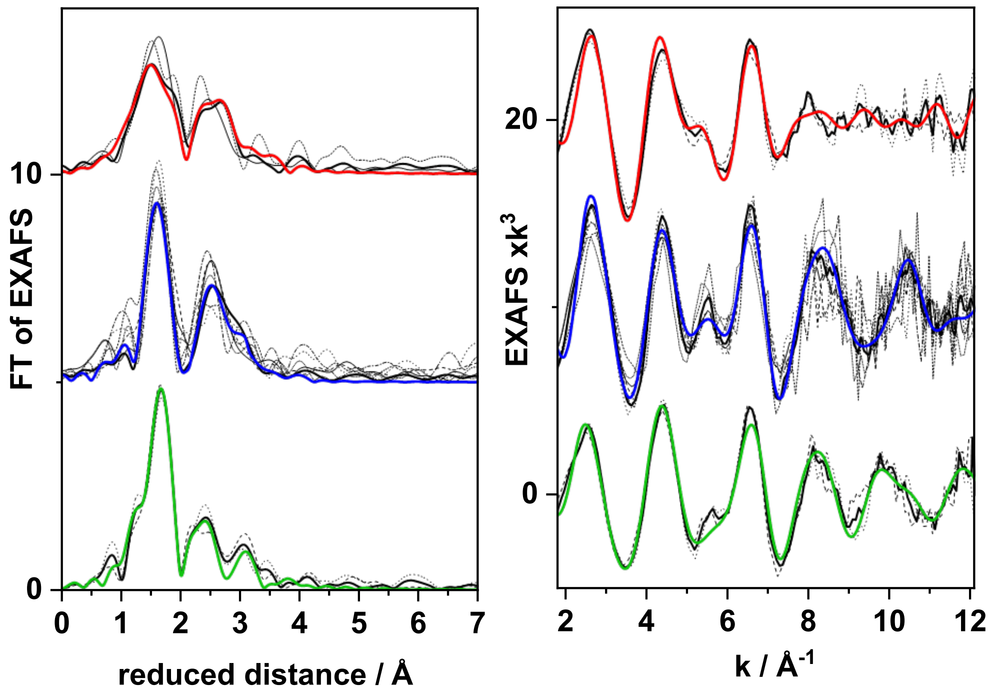

Supplementary Figure 15. Left panel: Fourier-transforms of EXAFS spectra in the right panel (thin/thick black lines, experimental data of individual/mean spectra corresponding to the XANES spectra in Supplementary Figure 9; colored lines, simulations of mean spectra with parameters in **Supplementary Table 9**, spectra are vertically stacked for clarity).

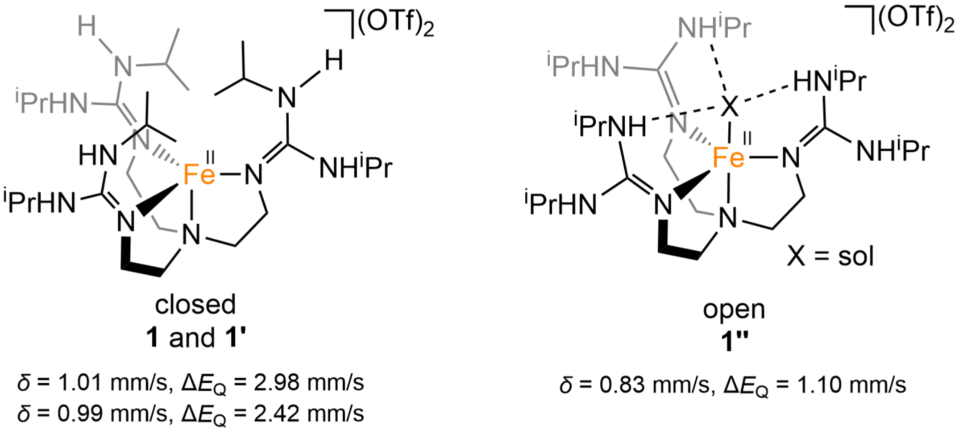

Supplementary Figure 16. Schematic representations of **1**, **1’** and **1’’**.

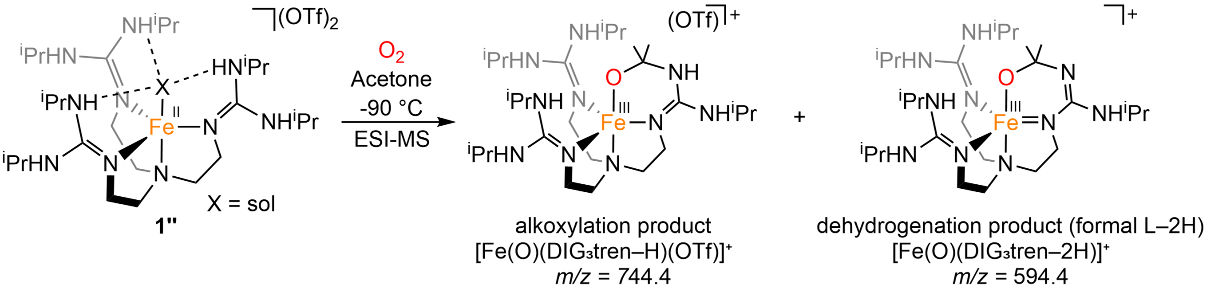

Supplementary Figure 17. Decomposition products of the reaction of **1’’** with O_2 detected by ESI-MS.

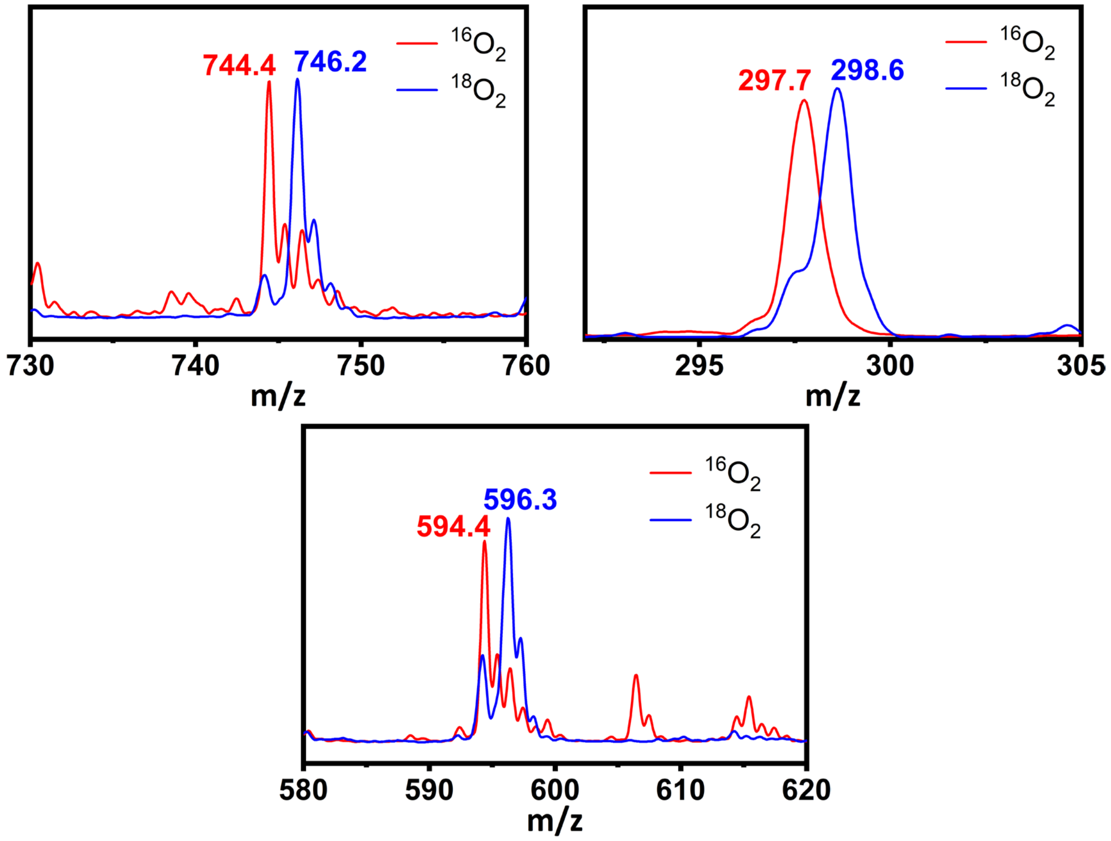

Supplementary Figure 18. ESI-MS spectra of reaction products of **1''** with $^{16}O_2$ (red) and $^{18}O_2$ (blue) showing the peaks assigned to $[Fe(O)(DIG_3tren-H)(OTf)]^+$ top left, $[Fe(O)(DIG_3tren-H)]^{2+}$ top right and $[Fe(O)(DIG_3tren-2H)]^+$ bottom.

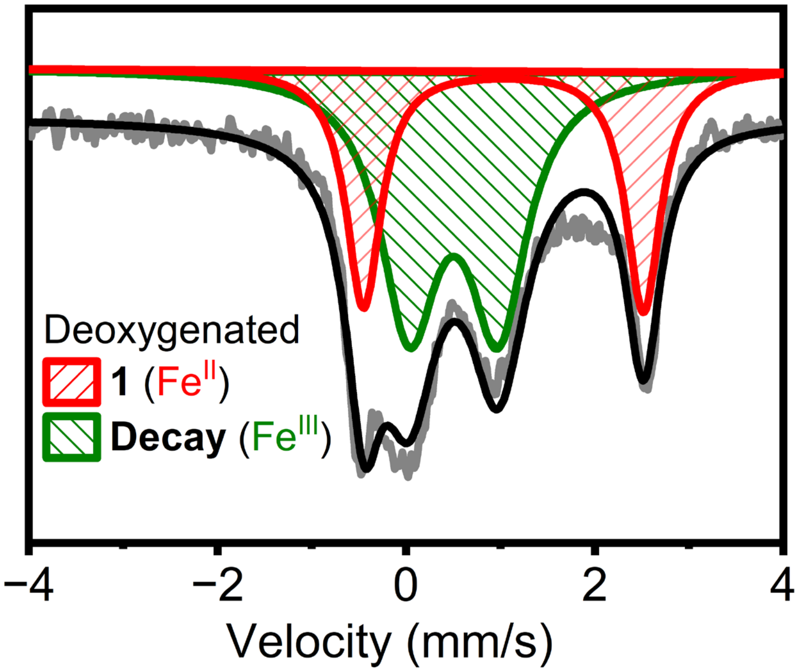

Supplementary Figure 19. Zero-field Mössbauer spectrum of deoxygenated acetone solution of **2** at 14 K (grey line: experimental spectrum; black line: simulated spectrum; green line: Fe(III) decay with δ = 0.51 mm/s, ΔE_Q = 0.94 mm/s, 60%; red line: **1**, 40%).

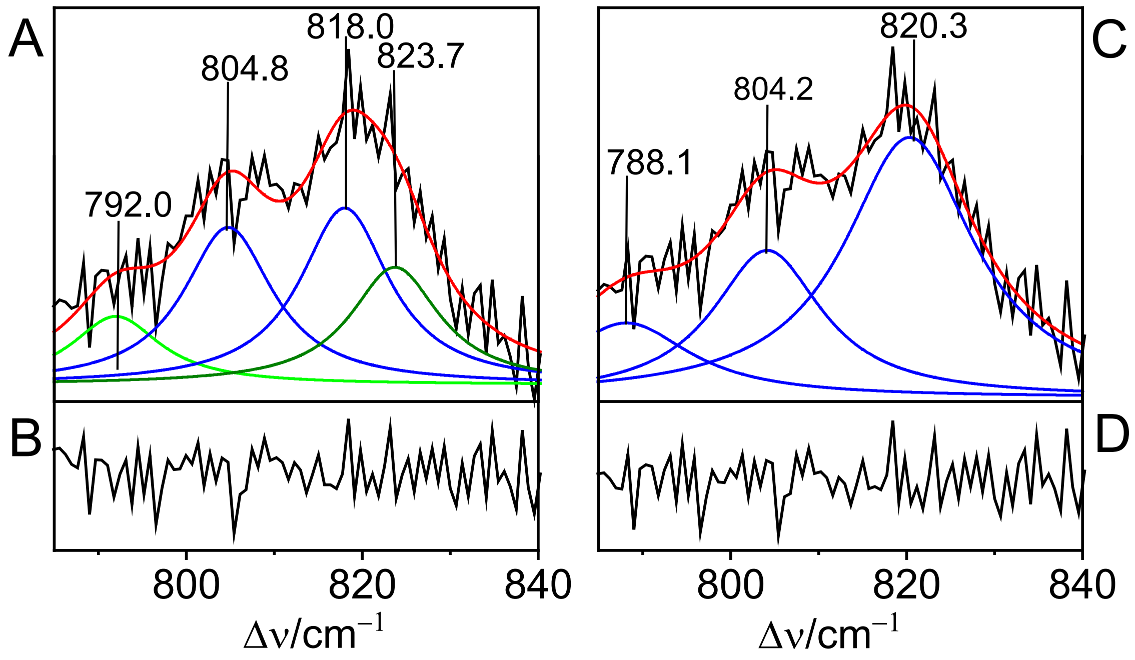

Supplementary Figure 20. RR spectra of **2** prepared at −90 °C in acetone-d_6 using a mixture of $^{16}O_2$, $^{18}O_2$, ^{16}O-^{18}O. The spectra show the region between 785 and 840 cm^{-1}, after subtraction of the weak band of the solvent at 817 cm^{-1} from the experimental spectrum (black trace). **A**, four Lorentzian functions were fitted to the experimental spectrum, representing the complexes with $^{16}O_2$ (dark green), $^{16/18}O_2$ and $^{18/16}O_2$ (blue), and $^{18}O_2$ (light green). The red trace shows the cumulative fit. **B**, residuals of the fit in **A**; **C**, three Lorentzian functions (blue) were fitted to the experimental spectrum (black), which, however, cannot be related to individual complexes. The red trace represents the cumulative fit. **D**, residuals of the fit in **C**.

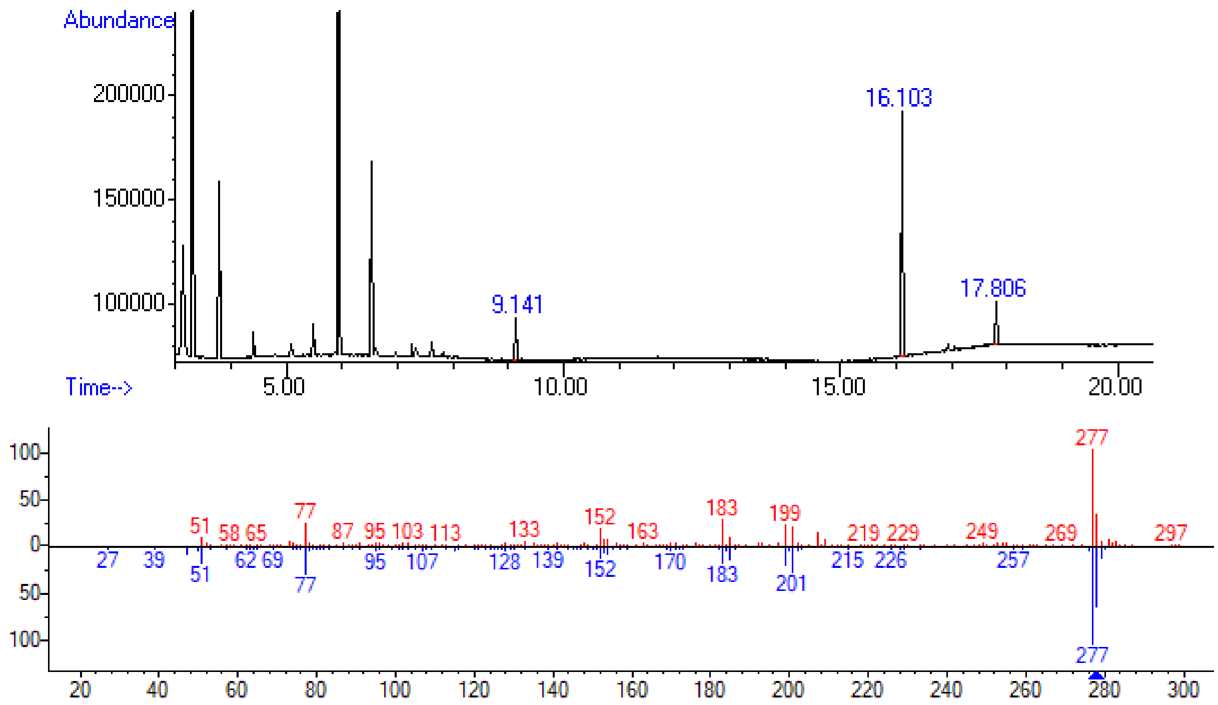

Supplementary Figure 21. Top: GC flame ionization detection spectrum after the reaction of **2** with 5 eq triphenylphosphine in acetone showing the formation of triphenylphosphine oxide. Biphenyl (9.141 min) as internal standard, triphenylphosphine (16.103 min), triphenylphosphine oxide (17.806 min). Note that the different retention times compared to the other substrates are due to a maintenance-related shortening of the column. Bottom: Comparison of the measured mass spectrum at 17.806 min (red) with the GC-library result for triphenylphosphine oxide (blue).

a) the end-on isomer

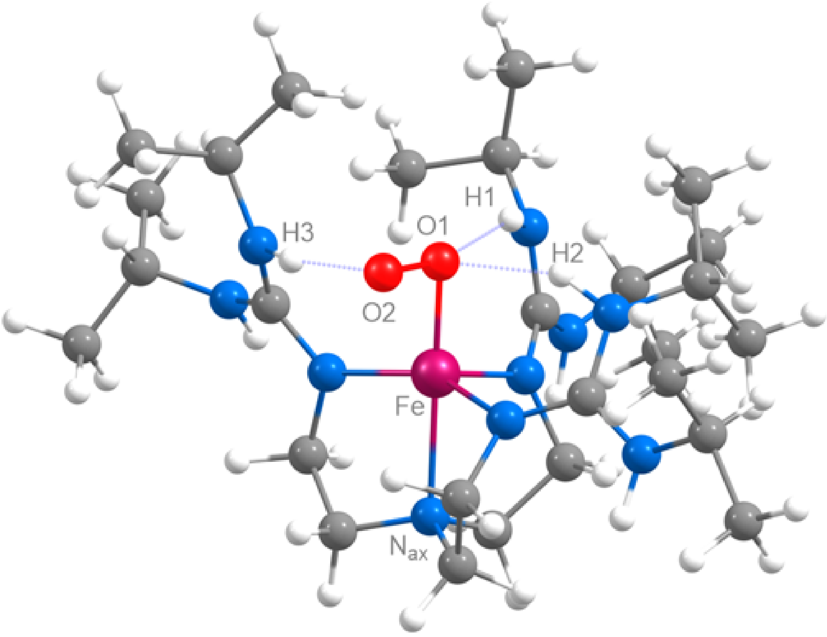

Fe-O1 = 1.764/1.801/1.816
Fe-O2 = 2.379/2.390/2.460
O1-O2 = 1.376/1.391/1.370
$\overline{\text{Fe-N}_{eq}}$ = 1.925/1.926/2.003
Fe-N_{ax} = 2.249/2.237/2.139

H-bonding system

O1-H1 = 1.916/1.947/1.952
O1-H2 = 2.017/1.996/1.823
O2-H3 = 1.731/1.740/1.877

$^{1/3/5}\mathbf{1}$

b) the side-on isomer

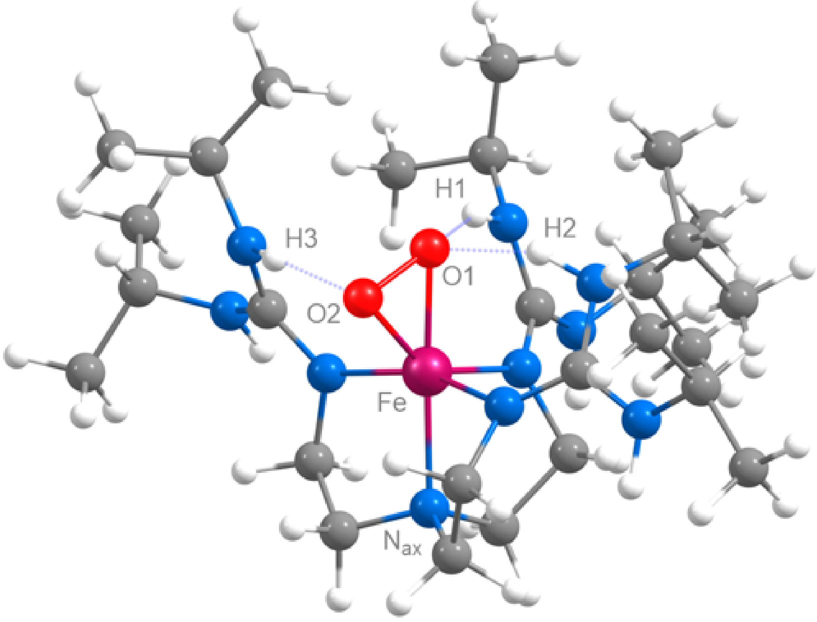

Fe-O1 = 1.878/1.919
Fe-O2 = 1.906/1.882
O1-O2 = 1.370/1.414
$\overline{\text{Fe-N}_{eq}}$ = 1.922/1.929
Fe-N_{ax} = 2.124/1.949

H-bonding system

O1-H1 = 1.857/1.898
O1-H2 = 1.980/1.818
O2-H3 = 1.933/1.746

$^{1/3/5}\mathbf{1'}$

Supplementary Figure 22. Geometric information of the (a) end-on and (b) the side-on isomers of the complex $[Fe(O_2)DIG_3tren]^{2+}$ with three (N)H-O hydrogen bond interaction. Calculations were done at the UBP86-D3(BJ)/B1 level in solvent. Bond lengths are in Å units and the order of numbers is a sequence of values for the singlet/triplet/quintet spin state.

a) the end-on isomer

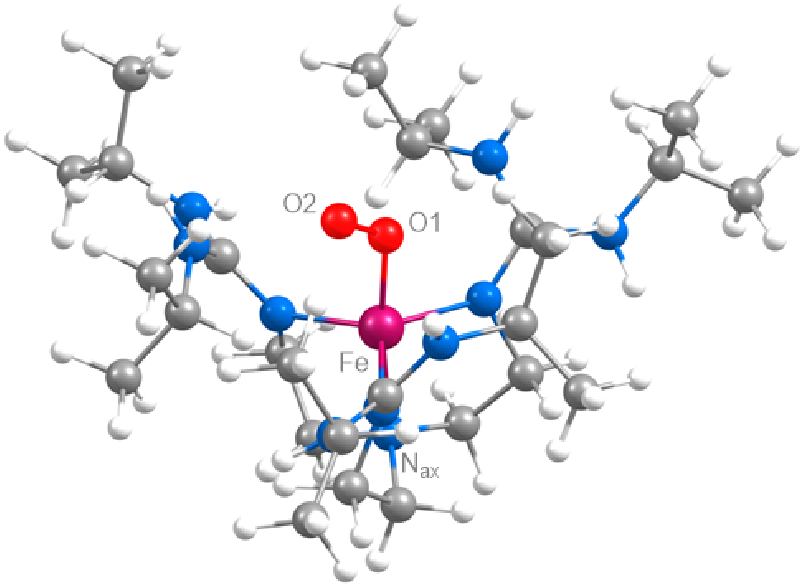

Fe-O1 = 1.764/1.795/1.791
Fe-O2 = 2.752/2.745/2.919
O1-O2 = 1.329/1.305/1.302
$\overline{\text{Fe-N}_{eq}}$ = 1.917/1.980/2.003
Fe-N_{ax} = 2.187/2.137/1.985

$^{1/3/5}$**2**

b) the side-on isomer

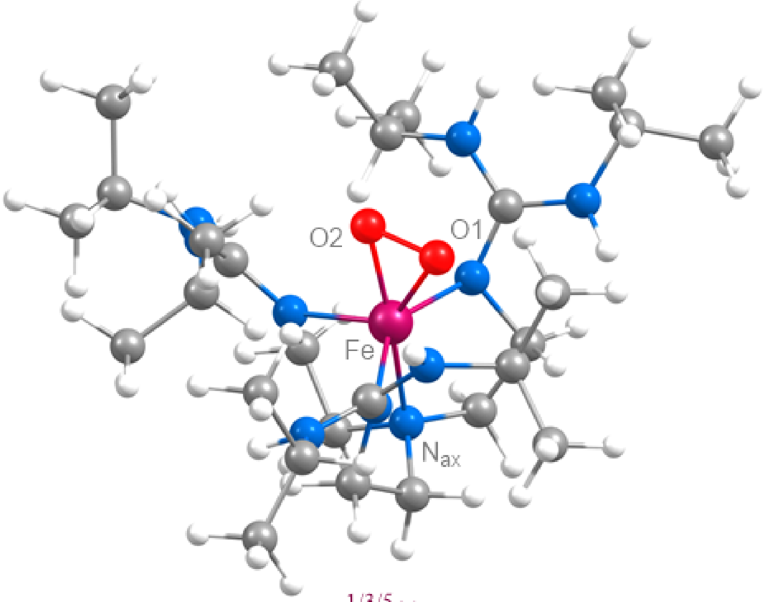

Fe-O1 = 1.816/1.852
Fe-O2 = 1.956/1.900
O1-O2 = 1.393/1.380
$\overline{\text{Fe-N}_{eq}}$ = 1.962/1.996
Fe-N_{ax} = 2.141/2.123

$^{1/3/5}$**2'**

Supplementary Figure 23. Geometric information of the (a) end-on and (b) the side-on isomers of the complex $[Fe(O_2)DIG_3tren]^{2+}$ without H-bonding interaction. Calculations were done at the UBP86-D3(BJ)/B1 level in solvent. Bond lengths are in Å units and the order of numbers is a sequence of values for the singlet/triplet/quintet spin state.

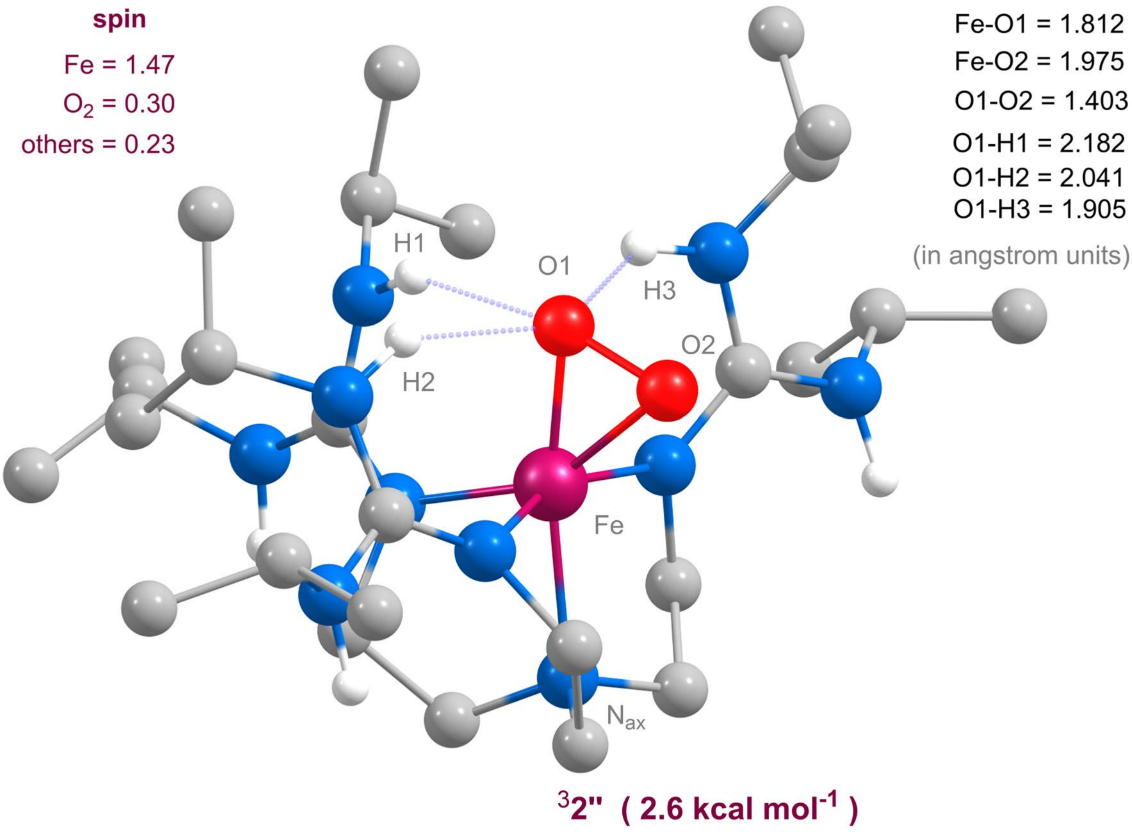

Supplementary Figure 24. Calculated geometric information of the complex $[Fe(O_2)(DIG_3tren)]^{2+}$ only with the proximal H-bonding interactions.

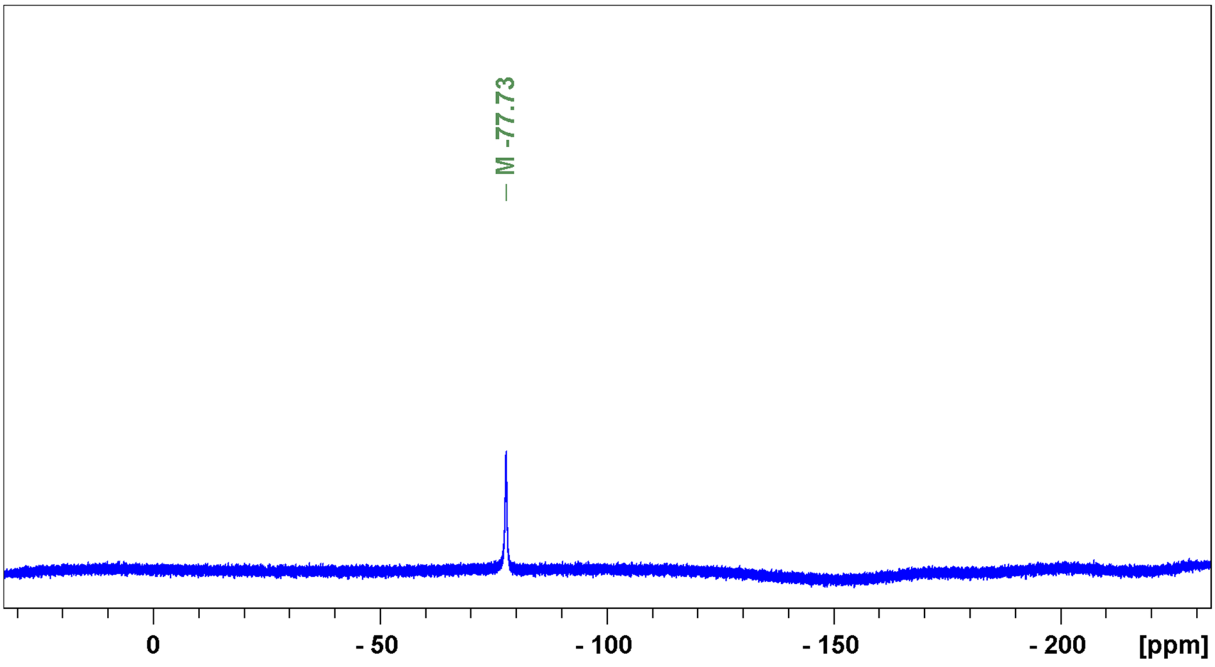

Supplementary Figure 25. ^{19}F-NMR spectrum of **3** in acetone-d_6 at 203 K (282 MHz).

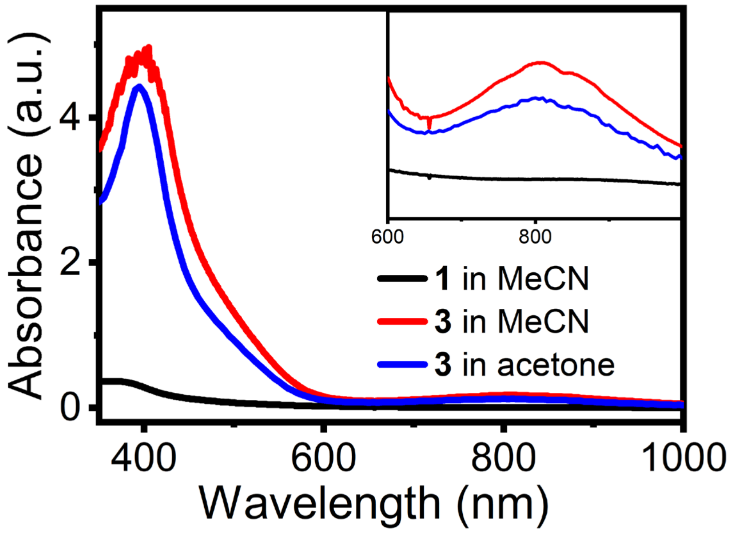

Supplementary Figure 26. UV/Vis spectrum of **1** (black) and **3** (0.5 mM) in acetonitrile (red) compared with **3** (0.5 mM) in acetone (blue) with zoom as inset.

a) with HB interaction

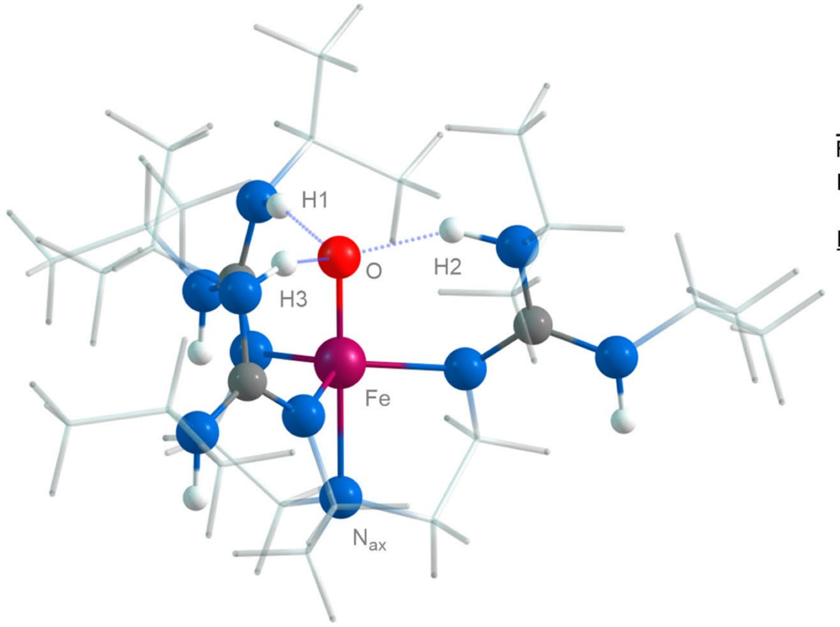

$^{1/3/5}$**3**

b) no HB interaction

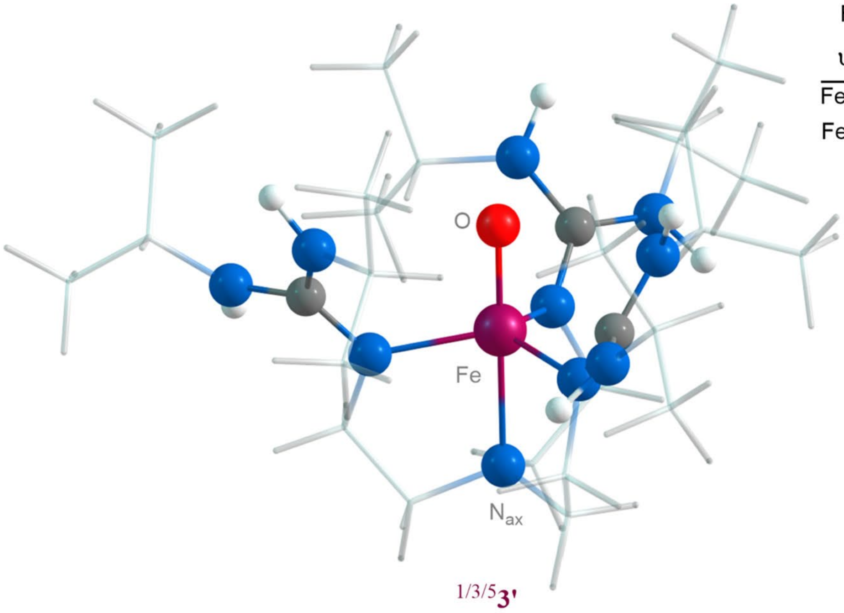

$^{1/3/5}$**3'**

Supplementary Figure 27. Geometric information of the complex $[Fe^{IV}(O)DIG_3tren]^{2+}$ **3** (a) with and (b) without H-bonding interaction. Calculations were done at the UBP86-D3(BJ)/B1 level in solvent. Bond lengths are in Å units and the order of numbers is a sequence of values for the singlet/triplet/quintet spin state.

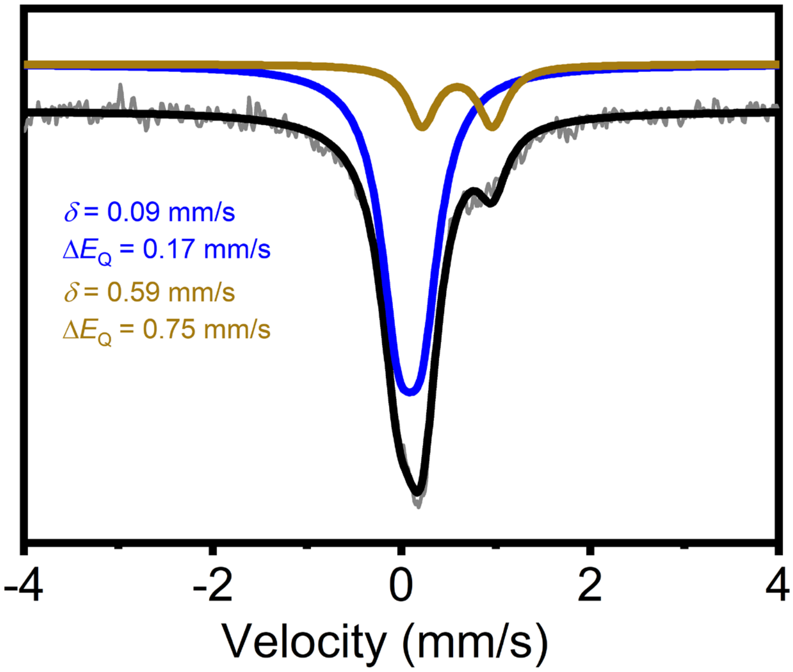

Supplementary Figure 28. Zero-field Mössbauer spectrum of a frozen acetone solution of ^{57}Fe enriched **3** (1 mM) at 14 K (grey line: experimental spectrum; black line: simulated spectrum; blue line: **3** with δ = 0.09 mm/s, ΔE_Q = 0.17 mm/s, 85%; brown line: decay product with δ = 0.59 mm/s, ΔE_Q = 0.75 mm/s, 15%). It is noteworthy that this spectrum misses the S=5/2 Fe(III) impurity that is detected at 5.8 K in applied magnetic fields (see Supplementary Figure 27).

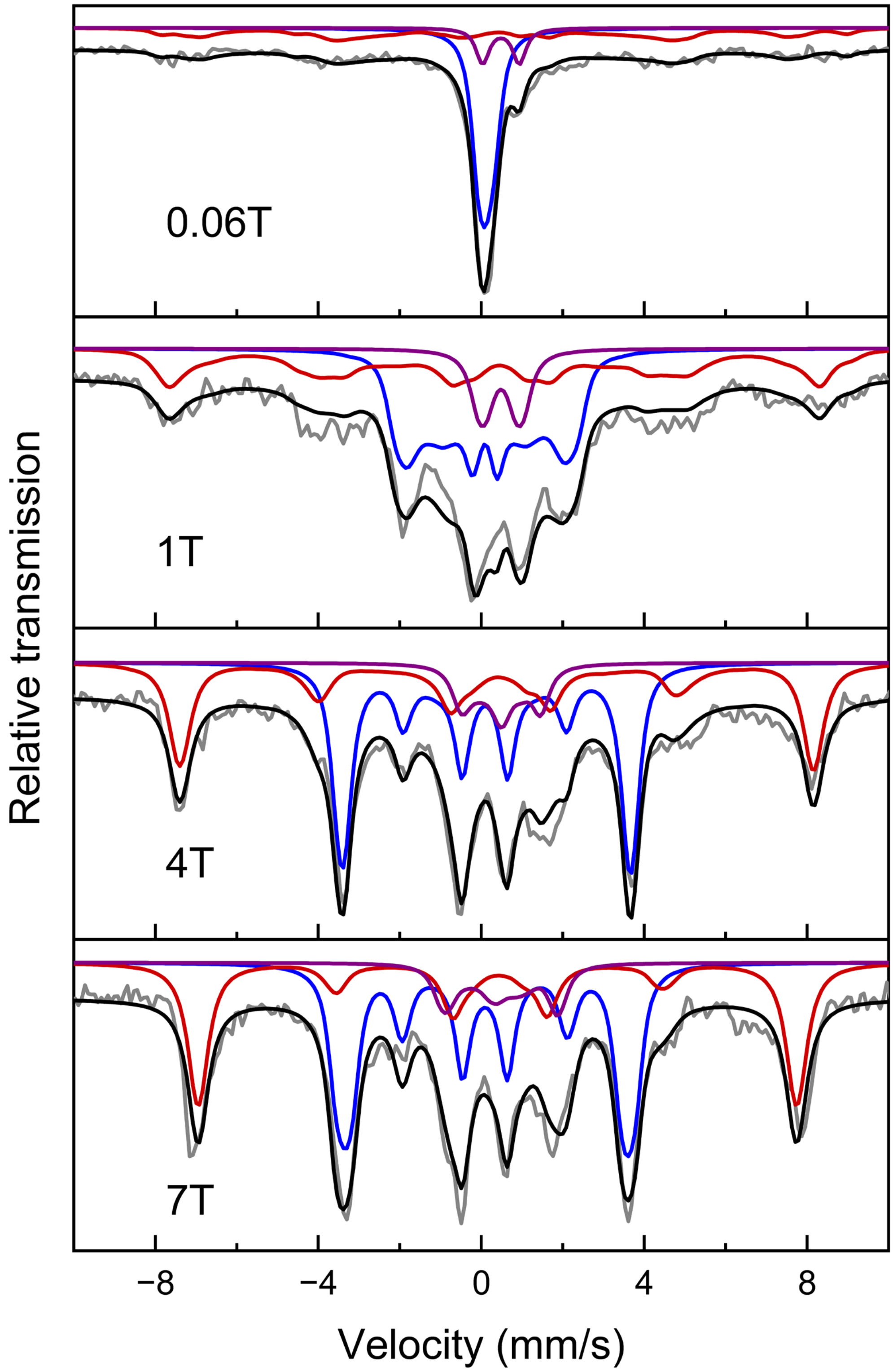

Supplementary Figure 29. Mössbauer spectra of **3** (blue spectra) recorded at 5.8 K in magnetic fields of 0.06, 1, 4 or 7 Tesla applied parallel to the direction of the gamma rays. Red and purple spectra correspond to Fe(III) impurities. All simulation parameters in Supplementary Table 3.

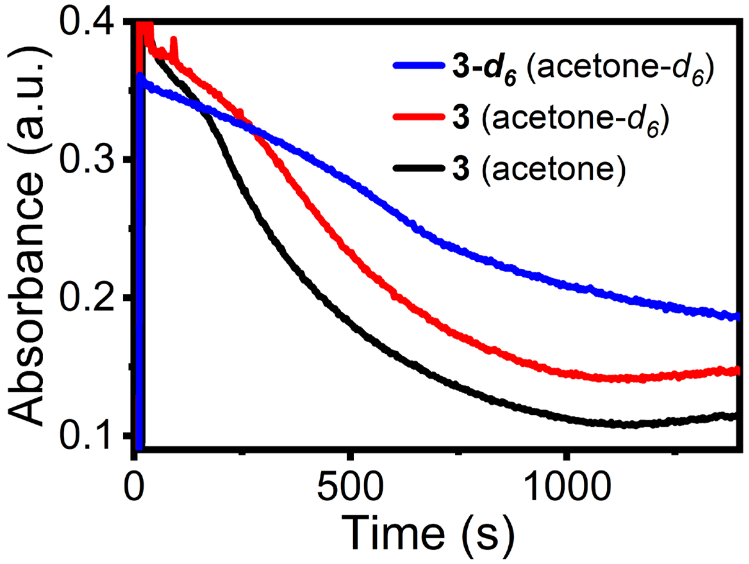

Supplementary Figure 30. Self-decay kinetics at 510 nm of **3** at −20 °C in acetone (black line), in acetone-d_6 (red line) and **3-d_6** in acetone-d_6 (blue line).

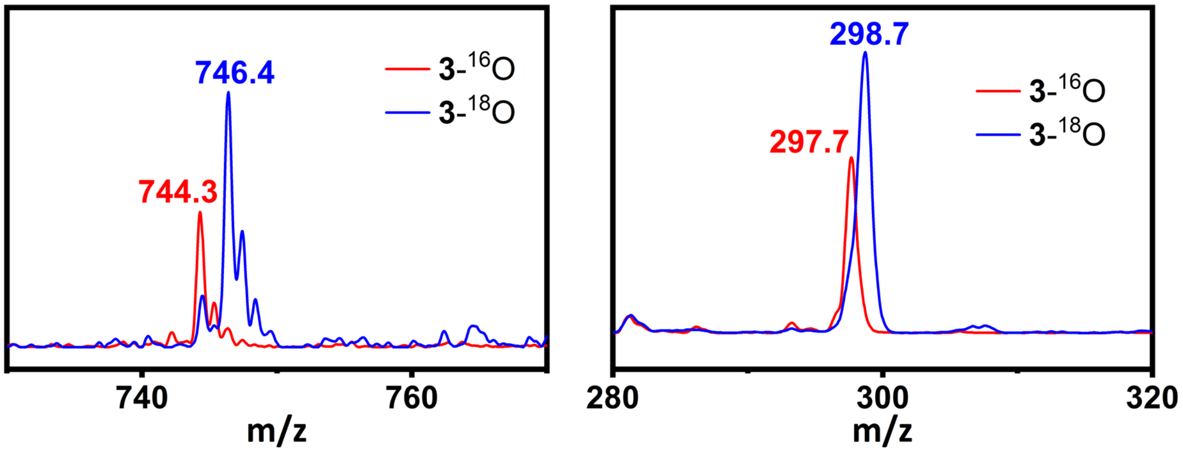

Supplementary Figure 31. ESI-MS spectrum of decay of **3** generated with ^{s}PhI^{16}O (red) and ^{s}PhI^{18}O (blue) showing the peaks assigned to [Fe(O)(DIG$_3$tren−H)(OTf)]$^+$ left and [Fe(O)(DIG$_3$tren−H)]$^{2+}$ right.

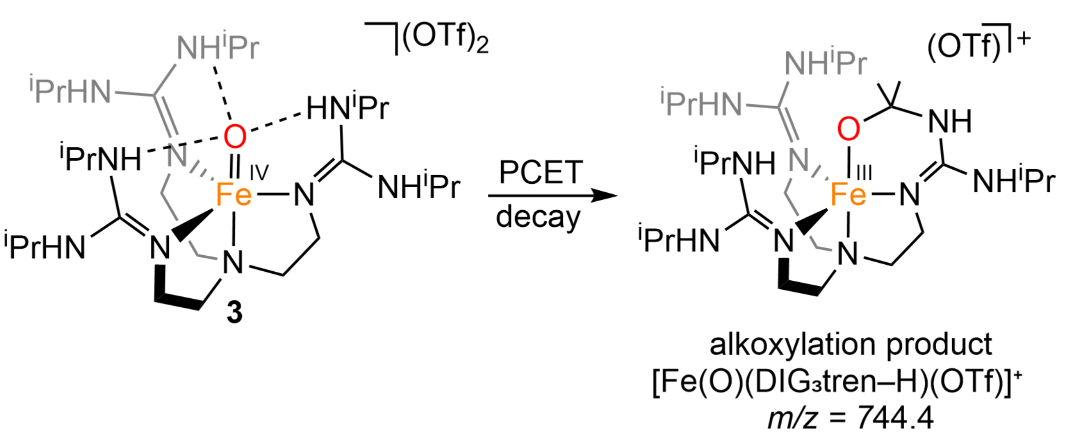

Supplementary Figure 32. Decay of **3** to the alkoxylated product detected by ESI-MS.

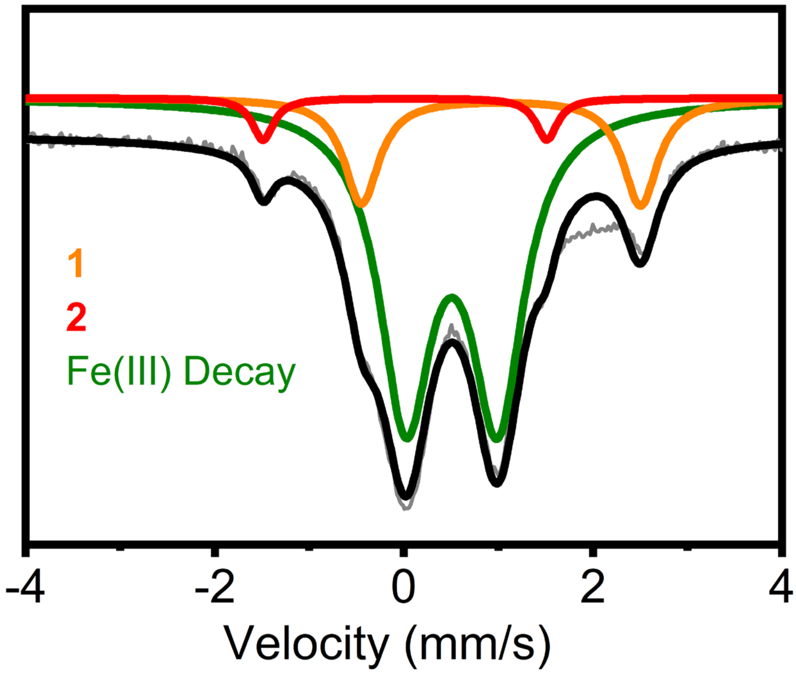

Supplementary Figure 33. Zero-field Mössbauer spectrum of the reaction products of ^{57}Fe enriched **1-*d*$_6$** (2 mM) with O_2 in acetone at 14 K (grey line: experimental spectrum; black line: simulated spectrum; red line: **2**, 5%; orange line: **1**, 19%; green line: Fe(III) decay products, 76%).

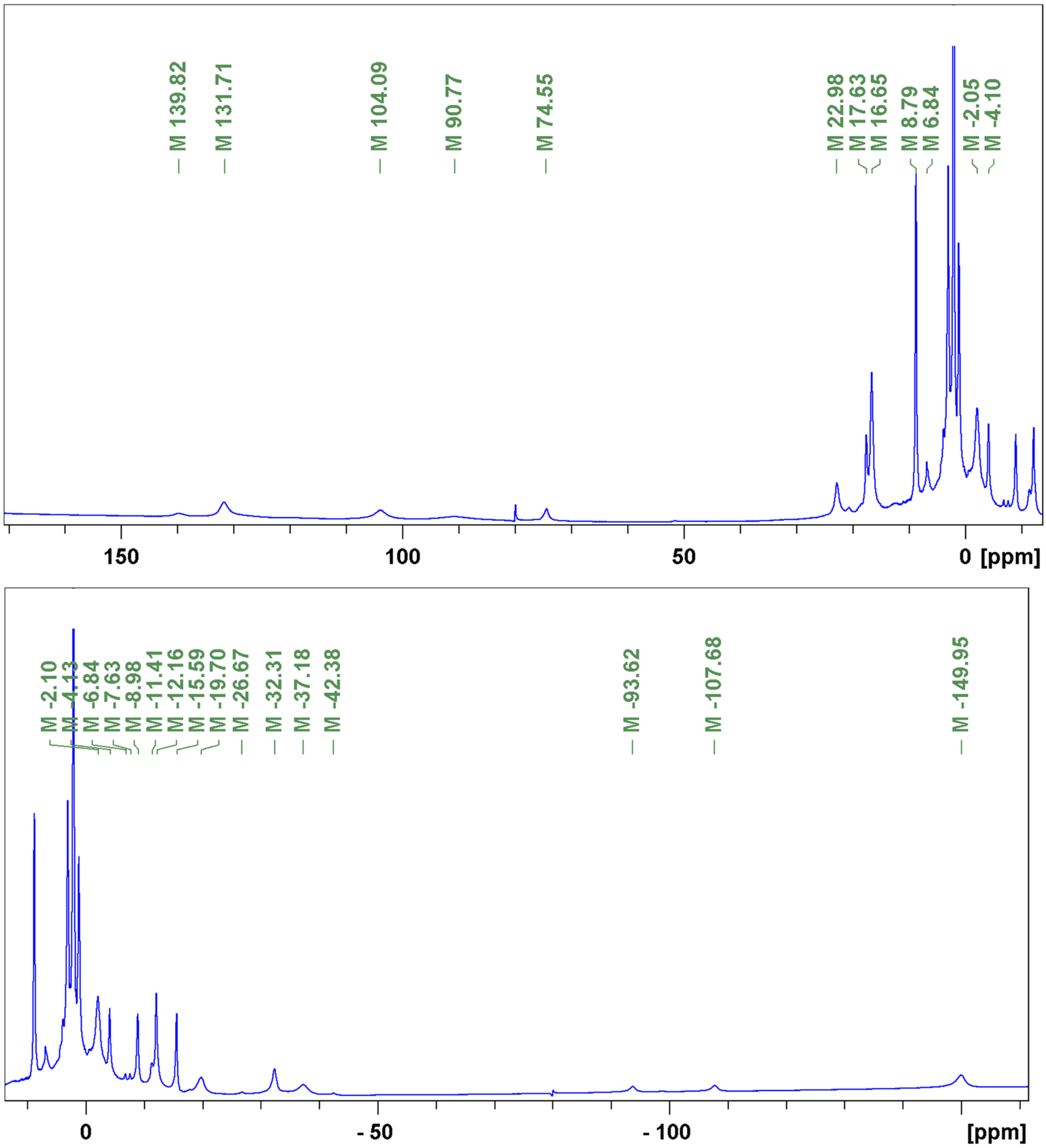

Supplementary Figure 34. ^{1}H-NMR spectra of **1-CO** in acetone-*d*$_6$ at 193 K (500 MHz).

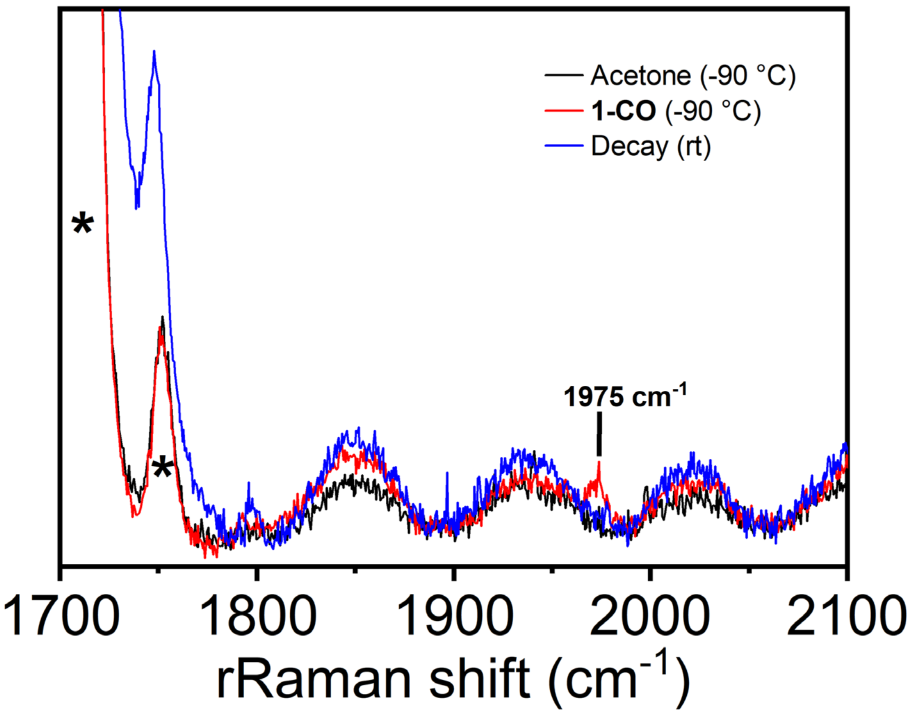

Supplementary Figure 35. rRaman spectra (λ_{exc} = 568 nm) of acetone at −90 °C (black), **1-CO** at −90 °C in acetone (red) and its decay at room temperature (blue). Solvent peaks are marked with an asterisk.

a) with HB interaction

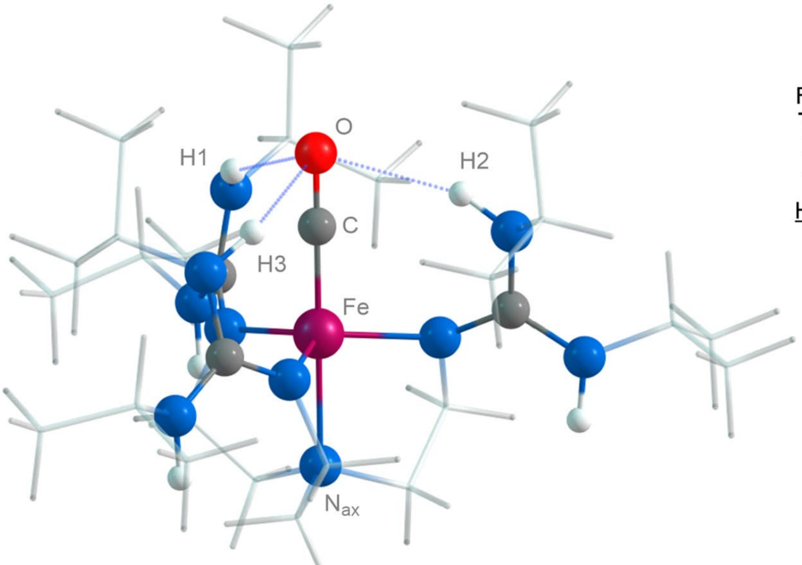

Fe-C = 1.728/1.726/1.960
C-O = 1.176/1.176/1.157
Fe-C-O = 172.4/179.2/174.1
$\overline{\text{Fe-N}}_{eq}$ = 1.973/1.997/2.037
Fe-N$_{ax}$ = 2.112/2.129/2.465

H-bonding system
O-H1 = 2.453/2.368/2.889
O-H2 = 2.274/2.364/2.631
O-H3 = 2.738/2.397/2.568

$^{1/3/5}$**1-CO**

b) no HB interaction

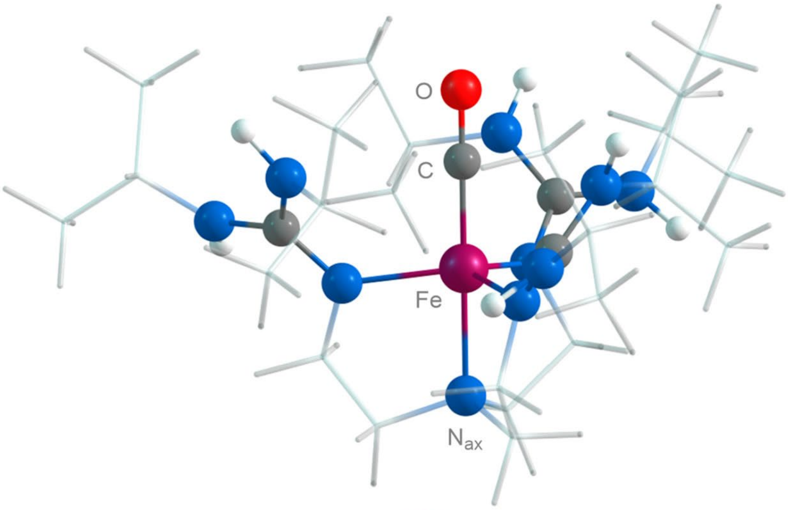

Fe-C = 1.746/1.748/2.005
C-O = 1.168/1.168/1.152
Fe-C-O = 179.9/179.9/168.7
$\overline{\text{Fe-N}}_{eq}$ = 1.972/1.979/2.022
Fe-N$_{ax}$ = 2.083/2.081/2.312

$^{1/3/5}$**1'-CO**

Supplementary Figure 36. Calculated geometric information of the complex $[Fe(CO)DIG_3tren]^{2+}$ (a) with and (b) without H-bonding interaction. Calculations were done at the UBP86-D3(BJ)/B1 level in solvent. Bond lengths are in Å units, angles are in ° units and the order of numbers is a sequence of values for the singlet/triplet/quintet spin state.

Determination of dissociation constants Kd and M value.

The following applies to the equilibrium

$$M + L \rightleftharpoons ML$$

(with M = complex **1**; L = O_2 or CO; ML = **2** or **1-CO**):

$$Ka = \frac{[ML]}{[M][L]} = \frac{1}{Kd} \qquad (1)$$

Ka: association constant (equilibrium constant K_{eq})
Kd: dissociation constant
[M], [L], [ML]: concentration of the respective compound

For a single binding site (1:1 binding stoichiometry), Kd equals the concentration of free ligand (O_2 or CO) at bound fraction Θ = 0.5:

$$K_d = [L] \text{ at } \Theta = 0.5$$

$$\Theta = \frac{A - A(\min)}{A(\max) - A(\min)} \qquad (2)$$

Θ: bound fraction
A: measured absorbance at each point of **2** or **1-CO**
A(min): minimum absorbance of deoxygenated **2** or **1/1-OTf** for CO
A(max): maximum absorbance of **2** or **1-CO**

For **2**, O_2/Ar mixtures (total pressure 1000 mbar) with varying partial pressures were used and the absorbance at 700 nm was measured.

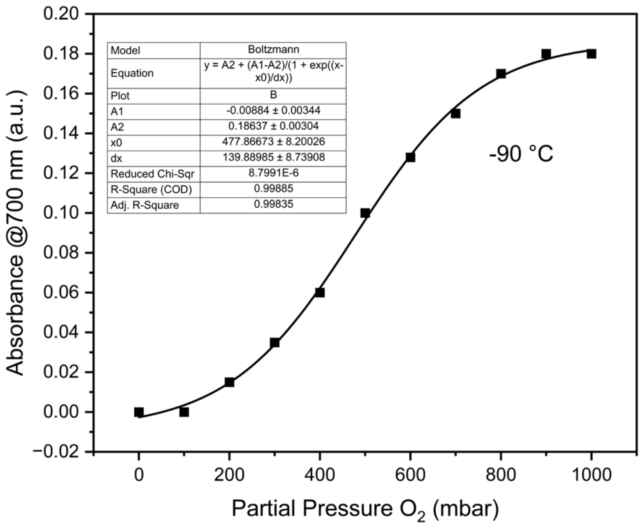

Supplementary Figure 37. Measured absorbance of **2** at 700 nm in acetone by using gas mixtures (O_2/Ar) with different partial pressures (total pressure 1000 mbar).

To determine the concentration of oxygen at the partial pressure $P(O_2) \approx 500$ mbar ($y = \Theta = 0.5$), Henry's law was used to determine Henry's constant H at 183.15 K, whereby the concentration of O_2 in a saturated acetone solution at 194.85 K ($[O_2]_{sat} = 13.62$ mM)[1] was used as an approximation, as the value for 183.15 K is not reported in the literature and the difference is expected to be minimal.

$$H = \frac{P(\mathrm{O2})}{[\mathrm{O2}]\mathrm{sat}} = \frac{1\ \mathrm{atm}}{0.01362\ \mathrm{M}} \approx 73.42\ \mathrm{atm/M} \quad (3)$$

$$[\mathrm{O2}] = \frac{0.5\ \mathrm{atm}}{73.42\ \mathrm{atm/M}} \approx 6.81\ \mathrm{mM} = Kd(\mathrm{O_2}) \quad (4)$$

Analogously, equilibrium constants were determined at −95 °C, −85 °C, and −80 °C, corresponding to $y = 0.5$ at ca. 0.4, 0.6, and 0.7 atm O_2, respectively (Supplementary Figure 44).

For **1-CO**, the absorbance was measured at 604 nm as a function of temperature. At ≈ −60 °C (213.15 K), $\Theta = 0.5$, whereby the literature value for the concentration of CO in a saturated acetone solution at 213.45 K was used[2], resulting in [CO] ≈ 11.25 mM = Kd(CO).

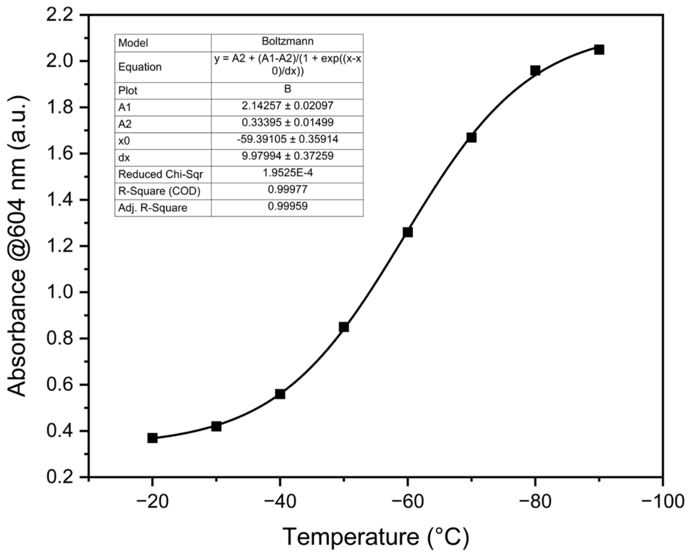

Supplementary Figure 38. Measured absorbance of **1-CO** at 604 nm in acetone by varying temperature.

The M value was determined as follows:

$$M = \frac{Kd\ (\mathrm{O2})}{Kd\ (\mathrm{CO})} = \frac{6.81\ \mathrm{mM}}{11.25\ \mathrm{mM}} \approx 0.6 \quad (5)$$

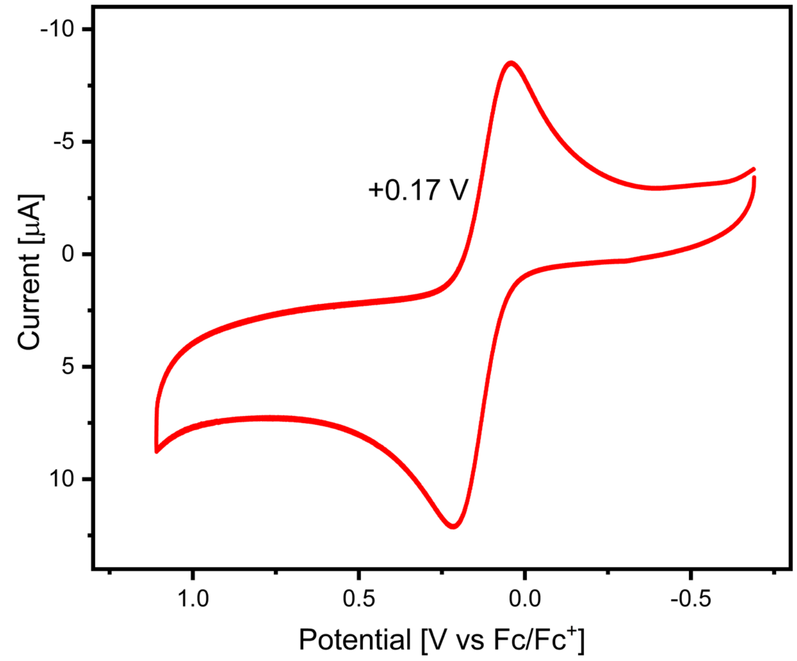

Supplementary Figure 39. Cyclic voltammogram of $[Fe(DIG_3tren)]^{2+}$ recorded in MeCN at room temperature using 0.1 M n-Bu_4NPF_6 as supporting electrolyte (scan rate 100 mV/s; working electrode: glassy carbon disk electrode; counter electrode: platinum wire; reference electrode: Ag/$AgNO_3$ (10 mM $AgNO_3$ and 0.1 M $[N(n\text{-}Bu)_4]PF_6$ in MeCN).

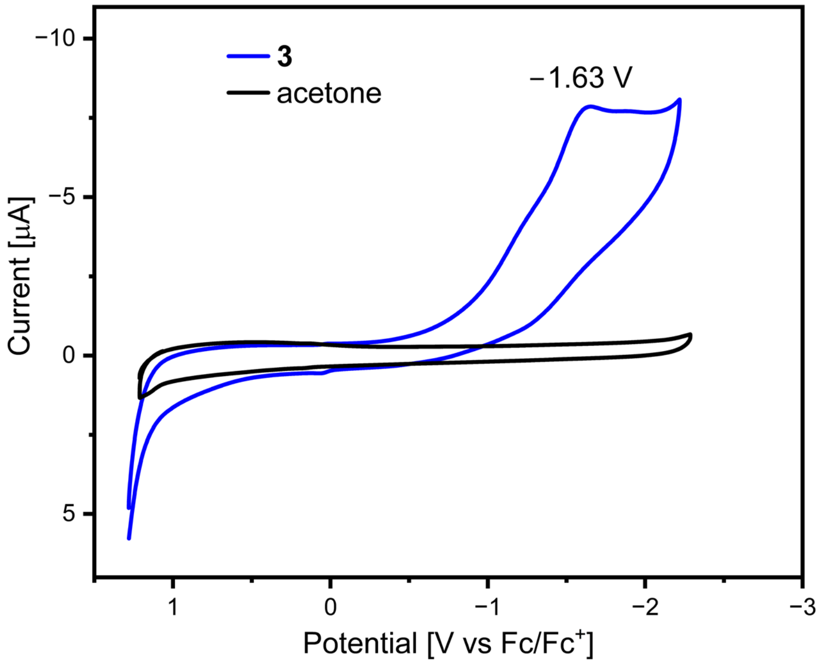

Supplementary Figure 40. Cyclic voltammogram of acetone (black) and **3** recorded in acetone (blue) at −80 °C using 0.1 M n-Bu_4NPF_6 as supporting electrolyte (scan rate 100 mV/s; working electrode: glassy carbon disk electrode; counter electrode: platinum wire; reference electrode: Ag wire quasi-reference electrode).

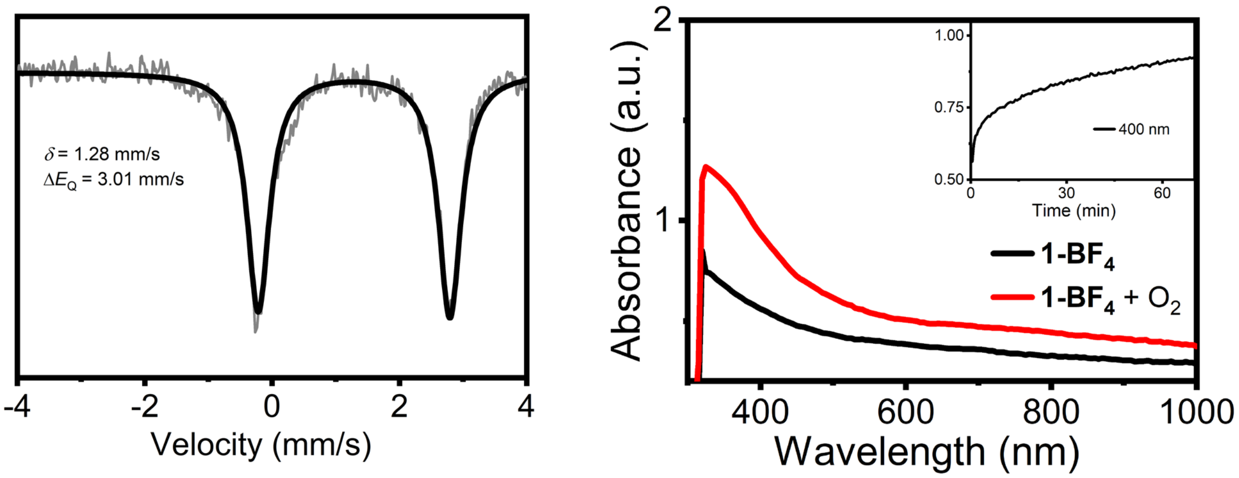

Supplementary Figure 41. Zero-field Mössbauer spectrum of **1-BF₄** at 14 K (left; grey line: experimental spectrum; black line: simulated spectrum with δ = 1.28 mm/s, ΔE_Q = 3.01 mm/s) and UV/Vis spectrum of **$1\text{-}BF_4$** and the reaction of **$1\text{-}BF_4$** with O_2 at −90 °C in acetone with time trace as inset (right).

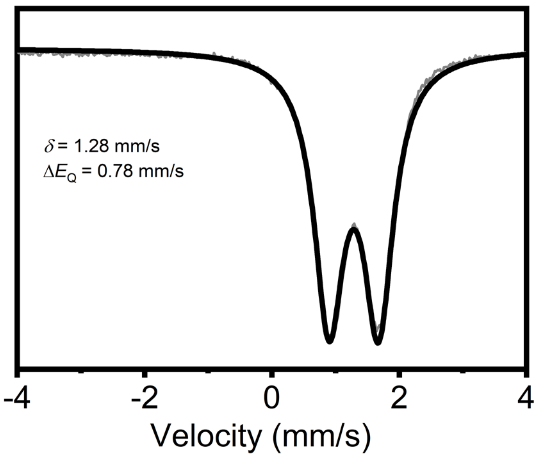

Supplementary Figure 42. Zero-field Mössbauer spectrum of $Fe(MeCN)_6(BPh_4)_2$ at 14 K (grey line: experimental spectrum; black line: simulated spectrum with δ = 1.28 mm/s, ΔE_Q = 0.78 mm/s).

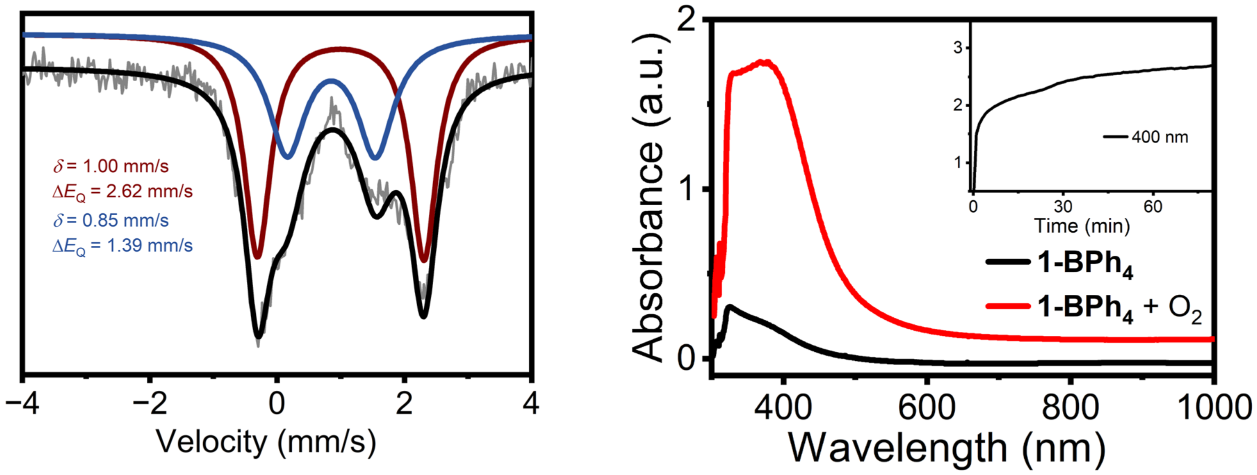

Supplementary Figure 43. Zero-field Mössbauer spectrum of **1-BPh$_4$** at 14 K (left; grey line: experimental spectrum; black line: simulated spectrum; dark red line with δ = 1.00 mm/s, ΔE_Q = 2.62 mm/s; blue line with δ = 0.85 mm/s, ΔE_Q = 1.39 mm/s) and UV/Vis spectrum of **1-BPh$_4$** and the reaction of **1-BPh$_4$** with O_2 at −90 °C in acetone with time trace as inset (right).

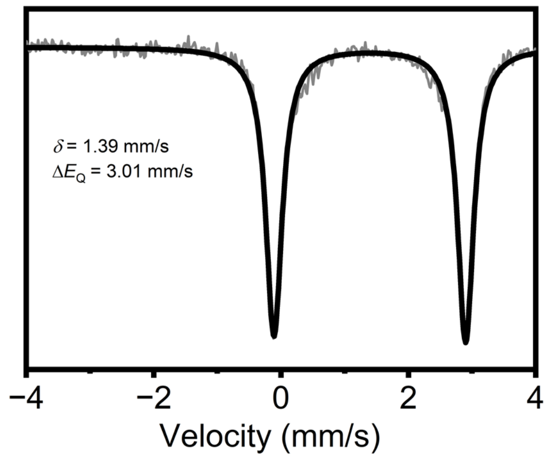

Supplementary Figure 44. Zero-field Mössbauer spectrum of $Fe(THF)_5(NTf_2)_2$ at 14 K (grey line: experimental spectrum; black line: simulated spectrum with δ = 1.39 mm/s, ΔE_Q = 3.01 mm/s).

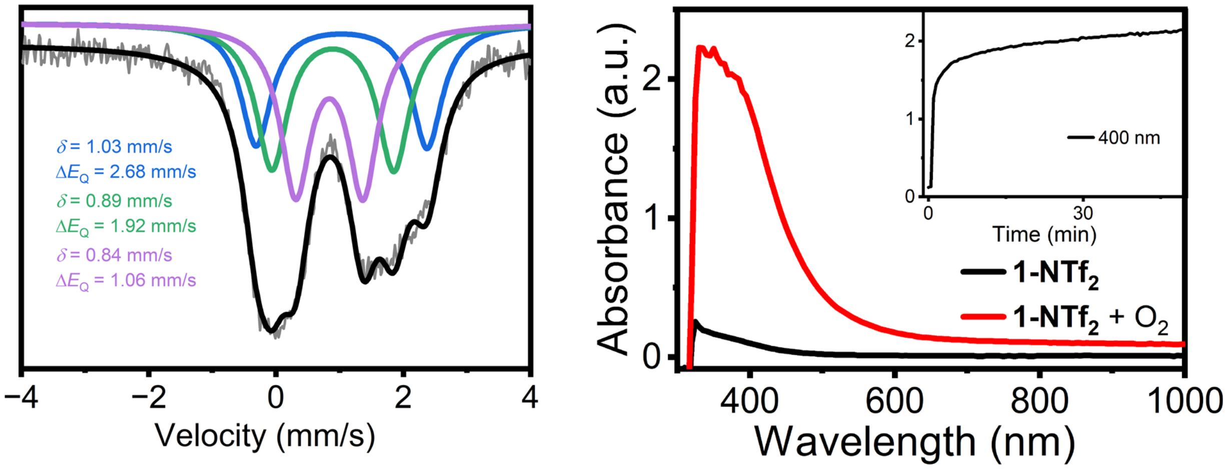

Supplementary Figure 45. Zero-field Mössbauer spectrum of **1-NTf₂** at 14 K (left; grey line: experimental spectrum; black line: simulated spectrum; blue line with δ = 1.03 mm/s, ΔE_Q = 2.68 mm/s; green line with δ = 0.89 mm/s, ΔE_Q = 1.92 mm/s; purple line with δ = 0.84 mm/s, ΔE_Q = 1.06 mm/s) and UV/Vis spectrum of **1-NTf$_2$** and the reaction of **1-NTf$_2$** with O_2 at −90 °C in acetone with time trace as inset (right).

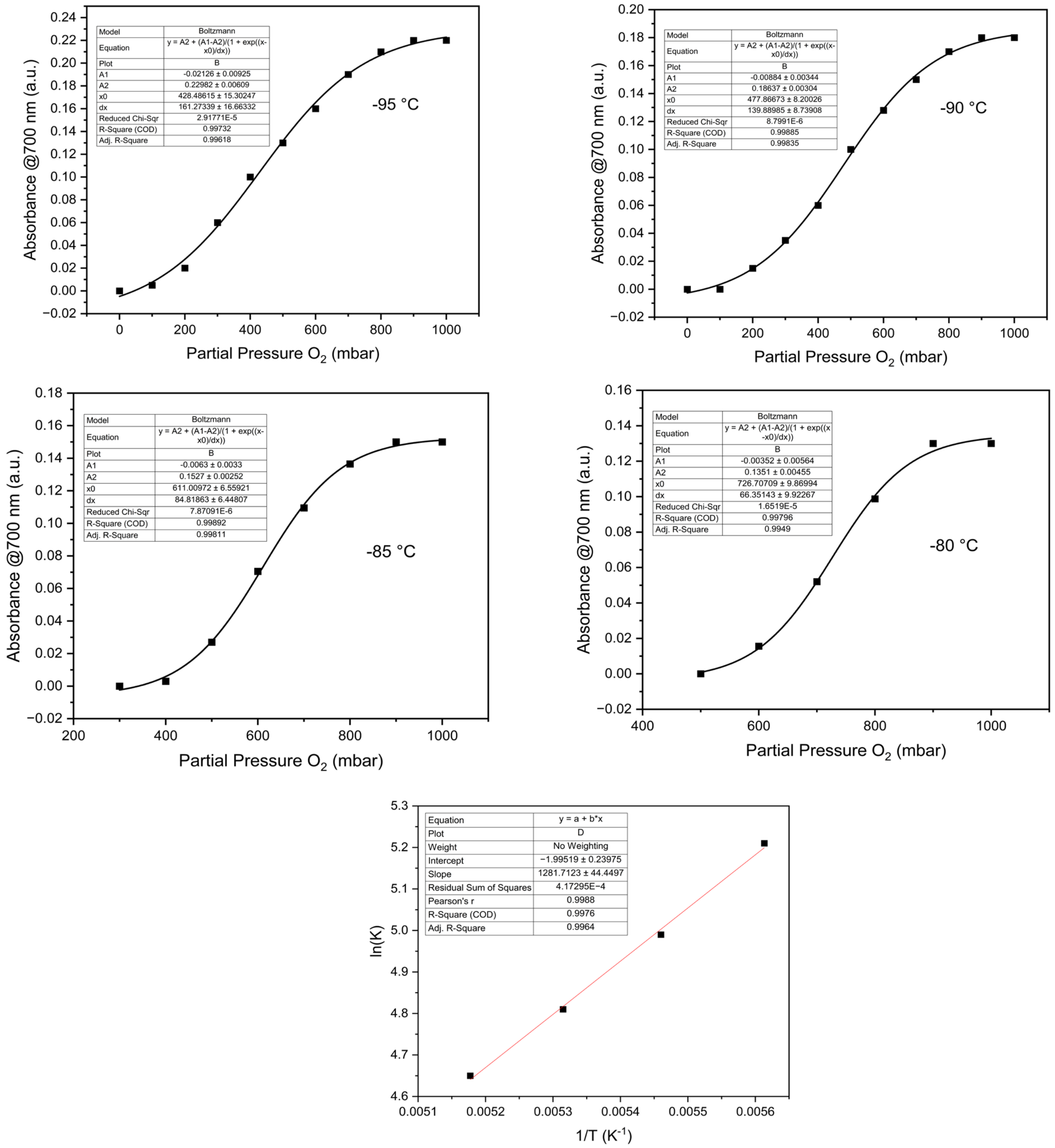

Supplementary Figure 46. Top and middle: Measured absorbance of **2** at 700 nm in acetone at −95 °C (top left), −90 °C (top right) −85 °C (middle left), −80 °C (middle right) using gas mixtures (O_2/Ar) with different partial pressures (total pressure 1000 mbar). Bottom: van't Hoff plot with the four equilibrium constants obtained ($K_{-80\,°C}$ = 104.88 M^{-1}; $K_{-85\,°C}$ = 122.36 M^{-1}; $K_{-90\,°C}$ = 146.84 M^{-1}; $K_{-95\,°C}$ = 183.55 M^{-1}).

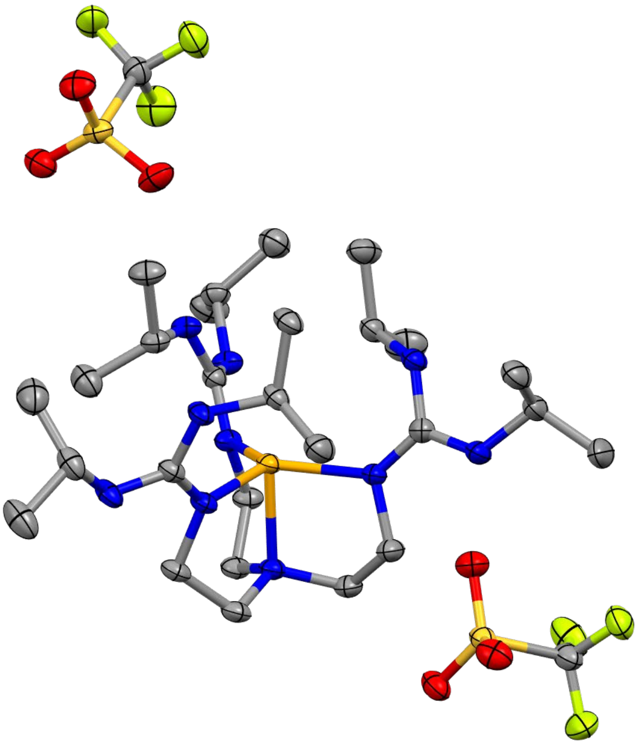

Supplementary Figure 47. Molecular structure of [Fe(DIG$_3$tren)](OTf)$_2$ obtained by XRD. Atoms are displayed as thermal ellipsoids at 50% probability level; H atoms and solvent molecule are omitted for clarity. Structural parameters are summarized in Supplementary Table 1. Atom types: Fe: orange; N: blue; C: grey; O: red; S: yellow; F: green. CCDC reference number: 2477483.

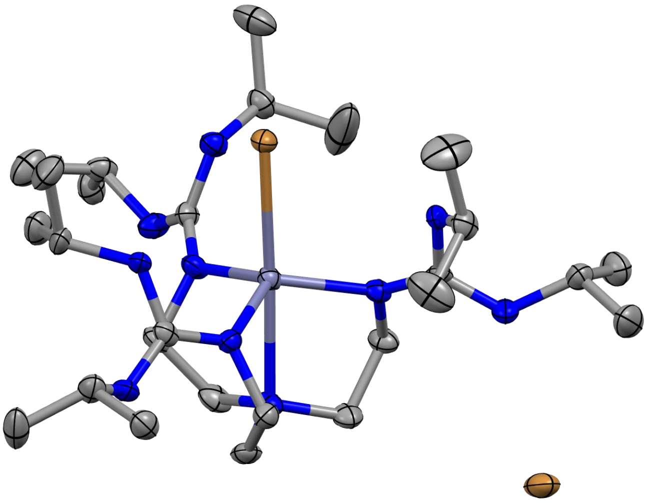

Supplementary Figure 48. Molecular structure of [Zn(DIG$_3$tren)(Br)](Br) obtained by XRD. Atoms are displayed as thermal ellipsoids at 50% probability level; H atoms and solvent molecule are omitted for clarity. Structural parameters are summarized in Supplementary Table 1. Atom types: Zn: violet; Br: brown; N: blue; C: grey. CCDC reference number: 2477484.

Supplementary Table 1. Data collection and refinement statistics (molecular replacement).

	$[Fe(DIG_3tren)](OTf)_2$	$[Zn(DIG_3tren)Br]Br$
**Data collection**		
Chemical formula	$C_{27}H_{60}FeN_{10}{\cdot}2(CF_3O_3S){\cdot}C_4H_{10}O$	$C_{27}H_{60}BrN_{10}Zn{\cdot}C_2H_3N{\cdot}Br$
Crystal system, space group	Monoclinic, $P2_1/c$	Monoclinic, $P2_1/c$
Temperature (K)	100	100
Cell dimensions		
a, b, c (Å)	18.05, 14.79, 17.93	14.10, 21.63, 13.93
α, β, γ (°)	90.00, 95.98, 90.00	90.00, 110.38, 90.00
V (Å^3)	4761.8	3984.7
Z	4	4
Radiation type	Mo $K\alpha$	Mo $K\alpha$
T_{min}, T_{max}	0.629, 0.745	0.652, 0.741
No. of measured, independent and observed [$I > 2\sigma(I)$] reflections	77389, 8712, 7705	84281, 7338, 5017
Resolution (Å)	0.83	0.83
R_{int}	0.032	0.176
$(\sin \theta/\lambda)_{max}$ (Å^{-1})	0.603	0.604
$I / \sigma I$	23.09	4.06
Completeness (%)	99.7	99.6
Redundancy	8.88	11.48
**Refinement**		
Resolution (Å)	0.83	0.83
No. reflections	8712	7338
No. of parameters	558	580
No. of restraints	4	0
$\Delta\rho_{max}$, $\Delta\rho_{min}$ (e Å^{-3})	0.67, −0.43	0.65, −0.69
$R[F^2 > 2\sigma(F^2)]$, $wR(F^2)$, S	0.036, 0.101, 1.02	0.044, 0.101, 1.02

Supplementary Table 2. Simulation Parameters for the magnetic Mössbauer spectra of **2**. As the spectra still contained partial signals from **1**, this was subtracted. The best results (best fits of the spectra after subtraction) were obtained by subtracting 15% of the spectra of **1**. The 5.8 K spectra were simulated with the following parameters (**2** as red; Fe(III) decay products as green):

%	43	42
S	1	5/2
g*	2.0 (iso)	2.0 (iso)
D	+20.8 cm^{-1}	-0.4 cm^{-1}
E/D	0.250	0.324
δ	0.016 mm/s	0.520 mm/s
ΔE_Q	+2.928 mm/s	-1.295 mm/s
η	0.508	0.596
A_{xx}, A_{yy}, A_{zz}	(-18.1, -18.6, -18.0) T	-20.5 T (iso)

* g-factors are not fitted (fixed at 2.0)

Supplementary Table 3. Simulation Parameters for the magnetic Mössbauer spectra of **3**. The 5.8 K spectra were simulated with the following parameters (**3** as blue; decay products as red and purple):

%	52	36	12
S	2	5/2	0
g	2.0 (iso)	2.0 (iso)	
D	+5.17 cm^{-1}	+0.69 cm^{-1}	
E/D	0.060	0.166	
δ	0.106 mm/s	0.428 mm/s	0.483 mm/s
ΔE_Q	-0.063 mm/s	+0.279 mm/s	-0.899 mm/s
η	0.721	0.560	0.628
A_{xx}, A_{yy}, A_{zz}	(-16.6, -14.2, -10.4) T	-21.0 T (iso)	

* g-factors are not fitted (fixed at 2.0)

Supplementary Table 4. Various SCF and Gibbs free energies of the end-on (**2**) and the side-on (**2'**) isomers of the complex $[Fe(O_2)DIG_3tren]^{2+}$ with three (N)H-O hydrogen bond interaction. Calculations were done at the UBP86-D3(BJ)/B2//B1 level in solvent.

a) The end-on isomer								
	OPT +ZPE	*ΔE*	SPE+ZPE	*ΔE*	*G*	*ΔG*	*G*/B2	*ΔG'*
^{1}**2**	-3026.148745	**9.4**	-3026.646255	**9.1**	-3026.186019	**10.2**	-3026.683529	**9.9**
^{3}**2**	-3026.163761	**0.0**	-3026.660796	**0.0**	-3026.202298	**0.0**	-3026.699332	**0.0**
^{5}**2**	-3026.156162	**4.8**	-3026.653615	**4.5**	-3026.195301	**4.4**	-3026.692754	**4.1**
b) The side-on isomer								
	OPT +ZPE	*ΔE*	SPE+ZPE	*ΔE*	*G*	*ΔG*	*G*/B2	*ΔG'*
^{1}**2'**	-3026.147261	**10.4**	-3026.646467	**9.0**	-3026.185869	**10.3**	-3026.685075	**8.9**
^{3}**2'**	-3026.159138	**2.9**	-3026.657377	**2.1**	-3026.197253	**3.2**	-3026.695492	**2.4**

Supplementary Table 5. Mulliken charges and spin densities of the complex $[Fe(O_2)DIG_3tren]^{2+}$ with three (N)H-O hydrogen bond interaction. Calculations were done at the UBP86-D3(BJ)/B1 level in solvent.

a) The end-on isomer								
	charge				spin			
	Fe	O1	O2	ligand	Fe	O1	O2	ligand
^{1}**2**	0.33	-0.23	-0.33	2.23	0.00	0.00	0.00	0.00
^{3}**2**	0.37	-0.26	-0.36	2.25	1.42	0.22	0.13	0.23
^{5}**2**	0.52	-0.20	-0.37	2.05	2.99	0.21	0.28	0.52
b) The side-on isomer								
	charge				spin			
	Fe	O1	O2	ligand	Fe	O1	O2	ligand
^{1}**2'**	0.25	-0.18	-0.34	2.27	0.00	0.00	0.00	0.00
^{3}**2'**	0.26	-0.31	-0.23	2.28	1.60	0.01	0.14	0.25

Supplementary Table 6. The variation of Fe-O and O-O vibration frequencies (in cm^{-1}) by ^{18}O-labelling of of the (a) end-on and (b) the side-on isomers of the complex $[Fe(O_2)DIG_3tren]^{2+}$ with H-bonding interaction.

a) the end-on species				
	$^{16}O1^{16}O2$	$^{16}O1^{18}O2$	$^{18}O1^{16}O2$	$^{18}O1^{18}O2$
υ_{Fe-O1}	604.6	604.4	583.5	583.3
υ_{Fe-O2}	105.8	104.1	105.4	103.8
υ_{O1-O2}	864.0	861.4	843.7	838.5
b) the side-on species				
	$^{16}O1^{16}O2$	$^{16}O1^{18}O2$	$^{18}O1^{16}O2$	$^{18}O1^{18}O2$
υ_{Fe-O1}	476.5	472.0	468.5	466.1
υ_{Fe-O2}	508.1	496.4	505.8	492.6
υ_{O1-O2}	893.4	877.6	874.9	858.3

Supplementary Table 7. Various SCF and Gibbs free energies of the end-on (**2**) and the side-on (**2'**) isomers of the complex $[Fe(O_2)DIG_3tren]^{2+}$ without H-bonding interaction. Calculations were done at the UBP86-D3(BJ)/B2//B1 level in solvent.

a) The end-on isomer								
	OPT +ZPE	*ΔE*	SPE+ZPE	*ΔE*	*G*	*ΔG*	*G*/B2	*ΔG'*
^{1}**2**	-3026.153619	**11.5**	-3026.651076	**12.0**	-3026.191200	**12.5**	-3026.688657	**12.9**
^{3}**2**	-3026.171984	**0.0**	-3026.670196	**0.0**	-3026.211074	**0.0**	-3026.709286	**0.0**
^{5}**2**	-3026.171324	**0.4**	-3026.669121	**0.7**	-3026.210561	**0.3**	-3026.708357	**0.6**
b) The side-on isomer								
	OPT +ZPE	*ΔE*	SPE+ZPE	*ΔE*	*G*	*ΔG*	*G*/B2	*ΔG'*
^{1}**2'**	-3026.142644	**18.4**	-3026.642086	**17.6**	-3026.180176	**19.4**	-3026.679618	**18.6**
^{3}**2'**	-3026.148380	**14.8**	-3026.648443	**13.7**	-3026.187032	**15.1**	-3026.687095	**13.9**

Supplementary Table 8. Mulliken charges and spin densities of the complex $[Fe(O_2)DIG_3tren]^{2+}$ without H-bonding interaction. Calculations were done at the UBP86-D3(BJ)/B1 level in solvent.

a) The end-on isomer								
	charge				spin			
	Fe	O1	O2	ligand	Fe	O1	O2	ligand
^{1}**2**	0.35	-0.09	-0.33	2.07	0.00	0.00	0.00	0.00
^{3}**2**	0.42	-0.08	-0.21	1.87	2.45	-0.27	-0.51	0.32
^{5}**2**	0.44	-0.09	-0.24	1.90	2.76	0.39	0.45	0.40
b) The side-on isomer								
	charge				spin			
	Fe	O1	O2	ligand	Fe	O1	O2	ligand
^{1}**2'**	0.29	-0.16	-0.25	2.12	0.00	0.00	0.00	0.00
^{3}**2'**	0.29	-0.13	-0.19	2.04	1.59	0.09	0.11	0.21

Supplementary Table 9. EXAFS simulation parameters of three iron complexes.

(coordination number, N [per Fe]; interatomic distance, R [Å]; Debye-Waller factor, $2\sigma^2$ x10^3 [$Å^2$]; fit error sum, R_F(1-3 Å) [%])

shell	Fe-N	Fe=/-O	Fe-O	Fe- -C	Fe- -C	R_F
**complex 1**						
N	4.0*	0.3±0.2	0.2±0.2	3.8±0.5#	5.2±0.5#	11.5
R	2.04±0.01	1.82±0.01	2.27±0.15	2.89±0.01	3.46±0.02	
$2\sigma^2$ x10^3	7±2	2*	2*	16±3§	16±3§	
**complex 3**						
N	4.0*	0.7±0.3	0.4±0.3	4.3±0.6#	4.7±0.6#	13.3
R	2.00±0.02	1.64±0.02	2.29±0.17	2.95±0.03	3.49±0.03	
$2\sigma^2$ x10^3	12±3	2*	2*	12±4§	12±4§	
**complex 2**						
N	4.0*	0.6±0.1	1.1±0.3 (0.2)	5.2±0.3#	3.8±0.3#	15.8
R	2.02±0.01	1.83±0.02	2.76±0.03 (2.17)	2.99±0.03	3.51±0.03	
$2\sigma^2$ x10^3	18±4	2*	2*	9±4§	9±4§	

*, fixed parameters; #, §, coupled parameters; the fits contain a further minor iron shell with N ca. 0.07-0.15 per Fe, R ca. 2.5 Å, and $2\sigma^2$ = 0.002 $Å^2$ due to a metallic iron contribution from the cryostat. The fit parameters and R_F values refer to the mean spectra and the errors represent the full parameter ranges of the fits of the individual spectra from 2-6 different samples. The values in parenthesis for the complex **2** refer to an alternative fit approach with a shorter second Fe-O bond.

Supplementary Table 10. Comparison between selected bond lengths of complexes **2** and **3** obtained by EXAFS and DFT (the value in parenthesis for the complex **2** refers to an alternative fit approach with a shorter second Fe-O bond).

complex	bond	bond length (EXAFS)	Bond length (DFT)
**2**	Fe−O(−O)	1.83 Å	1.80 Å
	Fe(−O)−O	2.76 Å (2.17 Å)	2.39 Å
	Fe−N_{ligand}	2.02 Å	2.08 Å
**3**	Fe−O	1.64 Å	1.69 Å
	Fe−N_{ligand}	2.00 Å	2.06 Å

Supplementary Table 11. Various SCF and Gibbs free energies of complex $[Fe(CO)DIG_3tren]^{2+}$ (a) with and (b) without H-bonding interaction. Calculations were done at the UBP86-D3(BJ)/B2//B1 level in solvent.

a) with H-bonding interaction								
	OPT +ZPE	*ΔE*	SPE+ZPE	*ΔE*	*G*	*ΔG*	*G*/B2	*ΔG'*
^{1}**1-CO**	-2989.123956	**4.0**	-2989.619894	**4.4**	-2989.161939	**4.6**	-2989.657877	**5.0**
^{3}**1-CO**	-2989.130307	**0.0**	-2989.626907	**0.0**	-2989.169281	**0.0**	-2989.665881	**0.0**
^{5}**1-CO**	-2989.095153	**22.1**	-2989.590993	**22.5**	-2989.135290	**21.3**	-2989.631129	**21.8**
b) no H-bonding interaction								
	OPT +ZPE	*ΔE*	SPE+ZPE	*ΔE*	*G*	*ΔG*	*G*/B2	*ΔG'*
^{1}**1'-CO**	-2989.148646	**6.4**	-2989.645821	**6.1**	-2989.187644	**6.4**	-2989.684818	**6.1**
^{3}**1'-CO**	-2989.158875	**0.0**	-2989.655565	**0.0**	-2989.197878	**0.0**	-2989.694568	**0.0**
^{5}**1'-CO**	-2989.121037	**23.7**	-2989.617313	**24.0**	-2989.161549	**22.8**	-2989.657825	**23.1**

Supplementary Table 12. Various SCF and Gibbs free energies of complex $[Fe^{IV}(O)DIG_3tren]^{2+}$ (a) with and (b) without H-bonding interaction. Calculations were done at the UBP86-D3(BJ)/B2//B1 level in solvent.

a) with H-bonding interaction								
	OPT +ZPE	*ΔE*	SPE+ZPE	*ΔE*	*G*	*ΔG*	*G*/B2	*ΔG'*
^{1}**3**	-2950.981853	**9.1**	-2951.305735	**21.1**	-2951.020304	**10.5**	-2952.215263	**22.6**
^{3}**3**	-2950.989476	**4.3**	-2951.319060	**12.8**	-2951.028442	**5.3**	-2952.229411	**13.7**
^{5}**3**	-2950.996372	**0.0**	-2951.339390	**0.0**	-2951.036965	**0.0**	-2952.251259	**0.0**
b) no H-bonding interaction								
	OPT +ZPE	*ΔE*	SPE+ZPE	*ΔE*	*G*	*ΔG*	*G*/B2	*ΔG'*
^{1}**3'**	-2950.788497	**19.9**	-2952.165833	**33.5**	-2950.869737	**22.0**	-2952.247073	**35.6**
^{3}**3'**	-2950.794210	**16.3**	-2951.309632	**24.9**	-2950.877536	**17.1**	-2952.263020	**25.6**
^{5}**3'**	-2950.820195	**0.0**	-2952.219181	**0.0**	-2950.904824	**0.0**	-2952.303810	**0.0**

Supplementary Table 13. Mulliken charges and spin densities of complex $[Fe^{IV}(O)DIG_3tren]^{2+}$ (a) with and (b) without H-bonding interaction. Calculations were done at the UBP86-D3(BJ)/B1 level in solvent.

a) without H-bonding interaction						
	charge			spin		
	Fe	O	others	Fe	O	others
^{1}**3**	0.56	-0.70	2.14	0.03	-0.22	0.18
^{3}**3**	0.50	-0.65	2.15	1.51	0.35	0.14
^{5}**3**	0.68	-0.61	1.93	2.96	0.52	0.52
b) no H-bonding interaction						
	charge			spin		
	Fe	O	others	Fe	O	others
^{1}**3'**	0.33	-0.37	2.04	0.00	0.00	0.00
^{3}**3'**	0.37	-0.36	1.99	1.51	0.39	0.10
^{5}**3'**	0.47	-0.35	1.89	2.84	0.71	0.46

References

1. Battino, R. *Oxygen and Ozone*. (Pergamon, Oxford New York, 1981).
2. *Carbon Monoxide*. (Pergamon, Oxford New York, 1990).
