# Supplementary material for: A mononuclear nonheme iron complex with higher affinity for O2 than CO via hydrogen bonding
Source: Nat Commun. 2026 May 5;17:6101. doi: 10.1038/s41467-026-72599-y (PMC13358151; doi:10.1038/s41467-026-72599-y)
Supplement: Supplementary file 2 — Transparent Peer Review File [file 41467_2026_72599_MOESM2_ESM.pdf]

nature portfolio

Peer Review File

A Mononuclear Non-heme Iron(II) Complex Exhibiting H-Bonding Mediated Higher Affinity for O_2 than for CO

Corresponding Author: Professor Kallol Ray

This file contains all reviewer reports in order by version, followed by all author rebuttals in order by version.

Version 0:

Reviewer comments:

Reviewer #1

(Remarks to the Author)
This paper by Ray and coworkers reports the synthesis and characterization of a Fe(II) complex having guanidine-based tripodal ligand with hydrogen-bonding sites and its reactivity with dioxygen to reversibly form an end-on Fe(IV)-peroxo complex stabilized by intramolecular hydrogen bonding. The Fe(II) complex shows reversible binding of CO to form a Fe(II)-CO complex. The selectivity in the binding of O2 over CO has been estimated by the ratio of binding constants to be 0.6, which is comparable with that of hemerythrin as a nonheme iron O2 carrier. The characterization of the complexes formed has been reasonably made using various methods, including EXAFS, XANES, NRVS, Mössbauer, resonance-Raman and absorption spectroscopies, and X-ray crystallography. The highlight of this work should be the favorable formation of a Fe(IV)-peroxo complex over a Fe(II)-CO complex and its reactivity in oxygen atom transfer to afford an Fe(IV)-oxo complex.
First, this referee cannot agree with the arguments described in this paper in association with hemoglobin, since the complexes discussed herein are not porphyrin complexes but complexes with a neutral non-porphyrin ligand. Rather, the chemistry should be discussed in association with hemerythrin, which shows comparable selectivity in the O2/CO binding.
Other than that, following issues should be considered prior to the publication of this work.
1. H-bonding interaction has been proposed to be formed not only with the proximal oxygen but the distal one of the peroxo ligand, as depicted in Fig 5. Have the calculations been made for the isomer in which H-bonding is formed only with the proximal oxygen? The situation should be included in the discussion to clarify the structure of the Fe(IV)-peroxo complex 2. It has been reported that similar amide NH protons form intramolecular hydrogen bonding with the metal-bound oxygen in end-on peroxo complexes reported so far as described in ref 62; however, hydrogen bonding has not been demonstrated between such NH groups and the distal oxygen. The hydrogen bonding with distal oxygen of an end-on peroxo ligand polarizes the O-O bond toward its heterolysis.
2. According to Fig 3a, 1-OTf also is converted to the “Fe(III) decay product”. Does this result indicate that the complex also reacts with O2 and decomposes to form the same Fe(III) complex? The reaction process of 1-OTf to the Fe(III) product should be also discussed.
3. It should be better to present a scheme showing the formation of complex 3 from 2 with PPh3.
4. According to Supp. Fig 24, the decomposition of complex 3 seems 2-step reaction. What is the final product of this reaction? Based on that, the reaction mechanism should be discussed.
5. The decay of complex 3 involves no hydrogen tunnelling, considering the ratio of the half lifetimes of 3 and 3-d6 in CH3CN. Why is the hydrogen tunneling not involved in the HAT reaction from hydrogen-bonded N-H to the oxo ligand in complex 3? The N-H bond dissociation energy may be ~92 kcal/mol or so.
6. In the CO binding, how does the hydrogen bonding or the N-H moieties contribute to or disturb the CO coordination? In the hemoglobin case, the N-H of a distal imidazole causes steric hindrance to disfavor the linear binding mode of CO. Is it the same as in the hemerythrin case? The authors need to provide at least a calculated structure of the proposed Fe(II)-CO complex to clarify the structure.
7. The CO vibration energy in 1-CO should be compared with that of CO-bound hemerythrin rather than that of CO-bound hemoglobin.
8. Thermodynamic parameters should be reported for the O2 binding to argue the stability of the peroxo complex based on the van’t Hoff equation.
9. On page 4, in the left column, the sentence “The calculated Fe-O distance at 1.802 Å, and O-O bonds at 2.391 Å, and 1.391 Å, respectively, are in good agreement with the experiment.” is not clear and should be revised.
10. In Supplementary figs 15 – 16, explanation of values in the figures should be added in each caption. Also, correct the disorder of numbers.

11. Elemental analysis data should be provided for all new complexes prepared and reported herein.

Reviewer #2

(Remarks to the Author)
In this manuscript, Ray et al. report a novel mononuclear iron complex supported by a guanidine-based ligand, which plays a critical role in providing steric protection and modulating the secondary coordination sphere. Thanks to this design, a rare iron(IV)–peroxido intermediate was observed upon binding of the Fe(II) complex with O_2, and the process was shown to be reversible. The steric shielding and hydrogen-bonding interactions afforded by the secondary coordination sphere facilitate two-electron transfer from the Fe(II) center to the bound dioxygen unit, while preventing irreversible O–O bond cleavage and formation of Fe–O–Fe dimeric species. Notably, this nonheme model exhibits a higher affinity for O_2 than for CO, contrasting with the behavior of hemoglobin. I find this work highly interesting; however, several major issues need to be clarified. I would therefore be willing to recommend the manuscript for publication in Nature Communications after major revisions.please find the foloowing issues：

1. Initial Fe(II) complex have two forms of existence, but only four-coordinate form can be crystallized. Thus, it is possible for separating these two species in solid state. Theoretically, authors could provide the separated characterization of the four-coordinate [FeII(DIG3tren)]2+.
2. I suggest that authors could use the non-coordinating Fe(II) salts, such as Fe(CH3CN)4(BF4)2, to prepare the pure four-coordinate [FeII(DIG3tren)]2+ complex. In this way, interference from 1-OTf can be eliminated.
3. If Fe(II) complex is a mixture in solution, it should show two different 19F peaks in its 19F-NMR spectrum, but there is only one 19F peaks in Supplementary Figure 3.
4. Is there a chemical equilibrium between species 1 and 1-OTf? How about the equilibrium constant?
5. In manuscript, authors stated: in the presence of oxygen, complex 1-OTf performs a fast irreversible electron transfer process to yield high-spin iron(III) decay products”. Is it an intermolecular electron transfer between 1-OTf and oxygen molecule? What is the oxidation potential of 1-OTf.
6. In Fig.3a, Mössbauer spectrum of a CO flushed acetone solution of 1/1-OTf shows only two species, 1 and 1-CO, why did 1-OTf disappear? According to author statement, 1-OTf cannot bind with CO molecule and remains unchanged in the solution.
So, Is it possible that the species (δ = 0.97 mm/s and ΔE_Q = 1.67 mm/s) in Mössbauer might not be 1-OTf, rather than some impurity?
7. Could authors provide more structural information about the decay species (Fe(III) species), such as using MS method? Is it stable in room temperature? Could it be Fe(III)-superoxide intermediate?
8. I suggest that EPR measurement of the possible Fe(IV)-peroxo intermediate can be supplemented, to further exclude the potential Fe(III)-superoxide form.
9. About Fe(IV)-oxido species 3, did authors optimize its structure by DFT calculation? They can show more geometrical details of species 3 in manuscript.
10.In Supplementary Figure 27, the IR spectrum of 1 should be added for comparison. Due to the weak rRaman peak of 1-CO species, I suggest that IR spectrum of 1-CO should be supplemented to further confirming the structure of Fe-CO center, including the 13CO labeled IR spectrum.

Reviewer #3

(Remarks to the Author)
The manuscript by Ray, Nam, and co-workers describes a non-heme iron complex that reversibly binds dioxygen. The authors have demonstrated that the dioxygen-bound form features an iron(IV)-peroxo species and that the complex has high affinity for dioxygen binding compared to carbon monoxide binding. This finding is interesting, as the number of nonheme iron complexes capable of reversible dioxygen binding is very small. Moreover, this complex shows exceptional discrimination for dioxygen binding versus CO. The manuscript is well written and the presentation and interpretation of the data are done at a very high level. I do, however, have one concern about the analysis, and I hope that the authors can clarify their approach.

My primary concern is in the assignment of 2 as a side-on Fe(IV)-peroxo complex. As noted by the authors, the mixed isotope experiment should allow one to distinguish between a side-on or an end-on peroxo complex. Specifically, a side-on species should have three peaks, while an end-on species should have four peaks. To my eyes, the resonance Raman data in Figure 4b have three peaks (near 792, 805, and 821 cm-1). I was somewhat surprised that these peaks are fit with five separate vibrations. In particular, the peak at 821 cm-1 is fit with three separate peaks, one at 823 cm-1 from the Fe(IV)-16O16O species, one at 817 from one of the Fe(IV)-16O18O species, and a peak from acetone-d6. It appears that the maximum for one experimental peak is not exactly 823 cm-1, as would be expected for the Fe(IV)-16O16O species. However, I wonder if other fits, with four bands instead of five, could be used to fit the data. This point is important, because, as far as I could tell, this is the decisive experiment for an end-on peroxo, and then hydrogen-bonding is inferred from this binding mode.

It is unfortunate that the experiments using the deuterated ligand could not help in establishing hydrogen-bonding between the supporting ligand and the bound peroxo. What about other options, such as ligands that have reduced hydrogen-bonding capabilities (such as electron-withdrawing groups on the hydrogen-bond donors)? If hydrogen-bonding is important, than the lack of interactions, or fewer interactions, should influence the K for dioxygen binding.

Version 1:

Reviewer comments:

Reviewer #1

(Remarks to the Author)
The authors have revised the manuscript properly in accordance with comments provided by reviewers. This reviewer would like to ask the authors to consider following three points.
1. The wrong M value of 1 for Hr in the original version confused me to say that it is comparable with the present case; however, if Hr does not form a CO complex, as for CO binding, it is not appropriate to compare the complex reported herein with Hr. To clarify the property of Hr in CO binding, not only in Fig 1(c), but the authors would be better to add “for Hr, M = 0” after “for Hb, M = 243” in the parenthesis on page 2.
2. Concerning the calculated structure of the CO complex shown in Suppl. Fig 36(a), although the authors claim that weak hydrogen bonding is operative among the oxygen of CO ligand and the amide N-H protons, this reviewer cannot imagine hydrogen bonding considering the interatomic distances in the figure (2.45, 2.27, 2.74 Å). Does the hydrogen bonding contribute to the comparable stability (M = 0.6) of the Fe(II)-CO complex to the peroxo complex 2?
3. To argue the unusual accessibility to the triplet Fe(IV) state as a peroxo (peroxido) complex, electrochemical data for determining the redox potentials of the Fe(II)-OTf complex (1-OTf) and complex 1 should be reported. The authors need to explain the reason why complex 1 can form a mononuclear trigonal bipyramidal end-on peroxo Fe(IV) complex, since the ligand set is not so electron-donating and the hydrogen bonding formed with the peroxo ligand is expected to reduce its electron-donating ability to stabilize a high-valent state. Although the authors have mentioned to the enhancement of electron acceptability of the superoxo ligand in the Fe(III)-superoxo intermediate by hydrogen bonding with the N-H protons, it should be discussed also based on the redox potentials of Fe(III)/Fe(II) and Fe(IV)/Fe(III) processes of the iron center.
4. Concerning the Suppl. Table 5, the numbers of complexes should not be 1 but 2.

Reviewer #2

(Remarks to the Author)
The revised has been careflly revised and the authors supplied a clear reponse on the concerns promoted by the referees. As indicated in my orignal comments, this woork is suitable for publication in Nature Communication and in this revised version the key intermediates are well charicterized by the specetroscopy methods as well as computational analysis following all reviewers comments and suggestions.I'd like to support this manuscript to be accepted for publication now.

Reviewer #3

(Remarks to the Author)
The authors have successfully addressed the issues I raised in my initial review. In particular, I find the resonance Raman analysis to be improved and in a reasonable form in this new version. I know support publication of this work.

Version 2:

Reviewer comments:

Reviewer #1

(Remarks to the Author)
The authors have responded to my comments properly and revised the manuscript appropriately. This is a nice work in iron-dioxygen chemistry, and the revised manuscript is very interesting, impactful and should be acceptable for publication in Nat. Commun..

Open Access This Peer Review File is licensed under a Creative Commons Attribution 4.0 International License, which permits use, sharing, adaptation, distribution and reproduction in any medium or format, as long as you give appropriate credit to the original author(s) and the source, provide a link to the Creative Commons license, and indicate if changes were made.
In cases where reviewers are anonymous, credit should be given to 'Anonymous Referee' and the source.
The images or other third party material in this Peer Review File are included in the article's Creative Commons license, unless indicated otherwise in a credit line to the material. If material is not included in the article's Creative Commons license and your intended use is not permitted by statutory regulation or exceeds the permitted use, you will need to obtain permission directly from the copyright holder.
To view a copy of this license, visit https://creativecommons.org/licenses/by/4.0/

Reviewer #1 (Remarks to the Author)

This paper by Ray and coworkers reports the synthesis and characterization of a Fe(II) complex having guanidine-based tripodal ligand with hydrogen-bonding sites and its reactivity with dioxygen to reversibly form an end-on Fe(IV)-peroxo complex stabilized by intramolecular hydrogen bonding. The Fe(II) complex shows reversible binding of CO to form a Fe(II)-CO complex. The selectivity in the binding of O2 over CO has been estimated by the ratio of binding constants to be 0.6, which is comparable with that of hemerythrin as a nonheme iron O2 carrier. The characterization of the complexes formed has been reasonably made using various methods, including EXAFS, XANES, NRVS, Mössbauer, resonance-Raman and absorption spectroscopies, and X-ray crystallography. The highlight of this work should be the favorable formation of a Fe(IV)-peroxo complex over a Fe(II)-CO complex and its reactivity in oxygen atom transfer to afford an Fe(IV)-oxo complex.

First, this referee cannot agree with the arguments described in this paper in association with hemoglobin, since the complexes discussed herein are not porphyrin complexes but complexes with a neutral non-porphyrin ligand. Rather, the chemistry should be discussed in association with hemerythrin, which shows comparable selectivity in the O2/CO binding. Other than that, following issues should be considered prior to the publication of this work.

Response: We would like to thank the reviewer for this insightful comment. However, we would like to clarify that hemerythrin (Hr) does not bind CO at all, and no CO-Hr adducts have been reported in the literature.[1,2] Accordingly, we have corrected the M value for hemerythrin in Figure 1 to "M = 0", as its CO affinity is effectively zero. Moreover, while hemerythrin is a non-heme protein, its active site is an asymmetric diiron center that binds O_2 as a hydroperoxo species, which is stabilized by a distinct hydrogen-bonding interaction and secondary coordination sphere. This mechanism differs fundamentally from the mononuclear end-on superoxo/peroxo or carbonyl binding modes observed in our system and in hemoglobin. For these reasons, we consider hemoglobin the more appropriate functional reference, as it displays the same phenomenology (reversible O_2 and CO binding and measurable CO/O_2 competition), which is the central comparison we aim to highlight. We, therefore, do not want to remove the discussion of hemoglobin from the manuscript completely, as it is relevant for the present study. Nevertheless, based on the criticism of the reviewer we have now removed the comparison with hemoglobin from the title of the manuscript. The new title is

"A Mononuclear Non-heme Iron(II) Complex with Higher Affinity for O_2 than for CO: Effect of H-Bonding Interactions".

We have also introduced the description of hemerythrin (Hr) in the abstract, and wherever possible we compare our system with hemerythrin (see for example Figures 1c and 1d)

1. H-bonding interaction has been proposed to be formed not only with the proximal oxygen but the distal one of the peroxo ligand, as depicted in Fig 5. Have the calculations been made for the isomer in which H-bonding is formed only with the proximal oxygen? The situation should be included in the discussion to clarify the structure of the Fe(IV)-peroxo complex 2.

Response: This is an interesting point raised by the reviewer. We, accordingly tried to obtain an isomer **2''** of the complex $[Fe(O_2)DIG_3tren]^{2+}$ with three proximal H-bonding interactions (please see the attached figure below); only a side-on structure is possible then. This isomer lies 2.6 kcal mol^{-1} higher in energy than the end-on isomer **2** with three H-Bonding interactions (one strong with a $(N)H\text{-}O_{distal}$ distance of 1.74 Ǎ and two weak ones with $(N)H\text{-}O_{proximal}$ distances of 2.02 Å and 1.92 Å), as shown in Fig. 7a in text. Notably, the spin populations are similar in the two cases (for **2**, Fe: 1.42, O2: 0.35, other: 0.23; for **2''**, Fe:1.47, O2: 0.30, other: 0.23.). Thus, **2''**, with three proximal H-Bonding interactions is also an iron(IV)-peroxo species, but the predicted side-on structure is not consistent with the experimental findings (for example rRaman data).

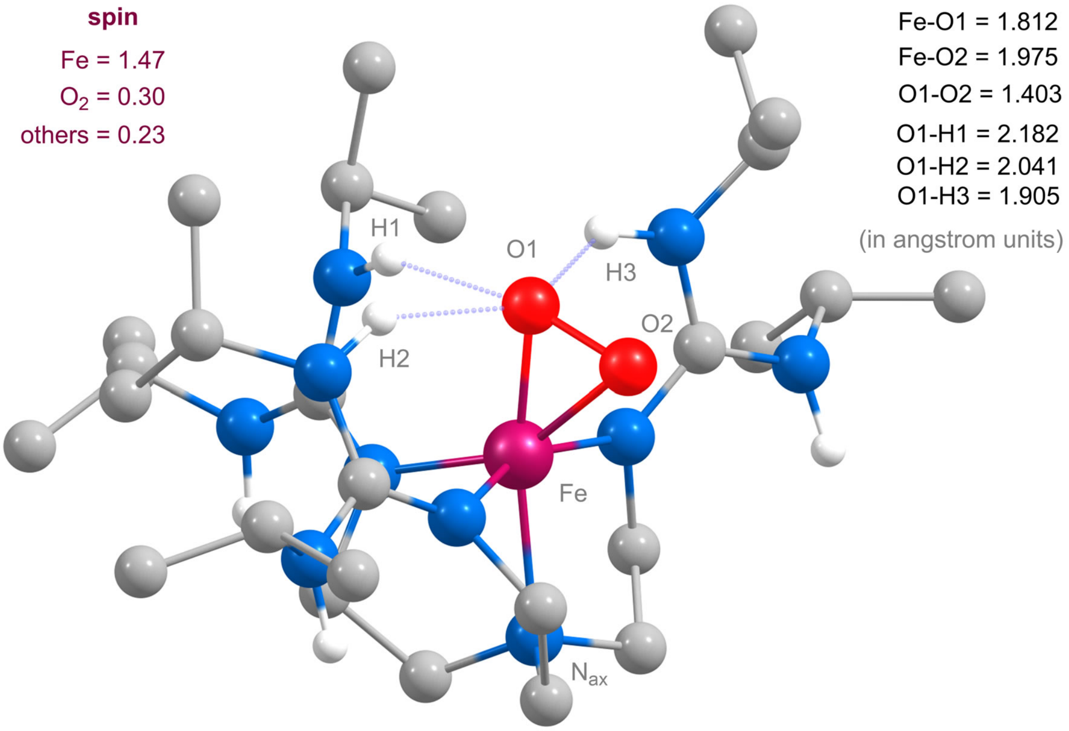

^{3}**2''** **(2.6 kcal mol^{-1})**

Supplementary Figure 23. Calculated geometric information of the complex $[Fe(O_2)(DIG_3tren)]^{2+}$ only with the proximal H-bonding interactions.

It has been reported that similar amide NH protons form intramolecular hydrogen bonding with the metal-bound oxygen in end-on peroxo complexes reported so far as described in ref 62; however, hydrogen bonding has not been demonstrated between such NH groups and the distal oxygen. The hydrogen bonding with distal oxygen of an end-on peroxo ligand polarizes the O-O bond toward its heterolysis.

Response: The reviewer is right. The O-O bond in **2** is indeed slightly polarized, as evident from its ability to perform oxygen atom transfer to PPh_3 to form PPh_3O and Fe^{IV}=O species (see Figs. 6 and 8). However, the extent of the polarization of the O-O bond will depend on the relative strengths of the H-bonding interactions involving the proximal and distal oxygen atoms. In our case, one stronger H-bonding with a (N)H-O_{distal} distance of 1.74 Ǻ and two weaker with (N)H-$O_{proximal}$ distances of 2.02 Å and 1.92 Å, respectively, presumably make the O-O bond in complex **2** slightly polarized, but at the same time stable against an irreversible O-O bond cleavage process.

2. According to Fig 3a, 1-OTf also is converted to the “Fe(III) decay product”. Does this result indicate that the complex also reacts with O2 and decomposes to form the same Fe(III) complex? The reaction process of 1-OTf to the Fe(III) product should be also discussed.

Response: We did not present our data properly in the previous version, which raised this concern of the reviewer. We apologize for this and have tried to present the data in a more clear way. Please note, that in acetone, OTf is not coordinated to iron as evident from the ^{19}F-NMR spectrum (Supplementary Figure 3), and hence **1-OTf** is not involved in the reaction at all. **1/1-OTf** exists as a mixture of four-coordinate and five-coordinate Fe(II) conformers (**1**, **1’** and **1’’,** Supplementary Figure 15) arising from the intrinsic flexibility of the DIG_3tren ligand. We have now added the Mössbauer data of the acetone solution of **1/1-OTf** in Figure 3a (top), showing the presence of different Fe(II) species in solution. Notably, the five-coordinate Fe(II) species (with lower ΔE_Q value and presumably involving solvent as an axial ligand) undergoes rapid, irreversible reaction with O_2 to form high-spin Fe(III) decay products. We propose that five-coordinate Fe(II) species bind O_2 to form a transient superoxo intermediate, which undergoes ligand-centered hydrogen-atom abstraction and oxidative decomposition (Supplementary Figure 16). This interpretation is strongly supported by ESI-MS measurements (Supplementary Figure 17) on an oxygenated solution of **1, 1’ and 1’’**, which show three

oxygen-isotope-sensitive signals. Two of them (m/z = 744.4 and 297.7) match the formulations $[Fe(O)(DIG_3tren–H)(OTf)]^+$ and $[Fe(O)(DIG_3tren–H)]^{2+}$ (calcd. m/z = 744.37 and 297.71), which are also observed during the decay of complex **3** (see response to Reviewer 1, point 4) and may correspond to the alkoxoiron(III) product. The third signal at m/z = 594.4 is specific to the O_2 reaction and corresponds to the dehydrogenation product $[Fe(O)(DIG_3tren–2H)]^+$ (calcd. m/z = 594.41). In all cases, the formation of these Fe(III) species reflects ligand-centered hydrogen-atom abstraction coupled to O–O bond cleavage, consistent with rapid oxidative decomposition of the Fe(II) species derived from **1''** in solution. In contrast, the four-coordinate Fe(II) species (**1** and **1'** with larger ΔE_Q values) with all DIG arms in the closed conformation react more slowly with O_2 and can reversibly form the Fe(IV)–peroxo intermediate **2** rather than Fe(III) decay products. To highlight the role of the transient Fe(III)-superoxide intermediate, we have now modified Figure 1d. This also helps us to compare our system with the mechanism of O_2 binding in hemerythrin (Figure 1c), where a similar Fe(III)-superoxide intermediate is proposed.

Notably, in Fig. 3a (middle), the broad Fe(III) signal was previously simulated as a single, broad component to avoid over-parameterization; however, its broadness strongly suggests the overlap of multiple, closely related Fe(III) species (this is now evident from a new simulation which clearly shows the involvement of different Fe(III) decay products and is included in updated Figure in the main text, together with the Mössbauer spectrum of the starting complex in acetone solution showing the formation of **1**, **1'** and **1''**).

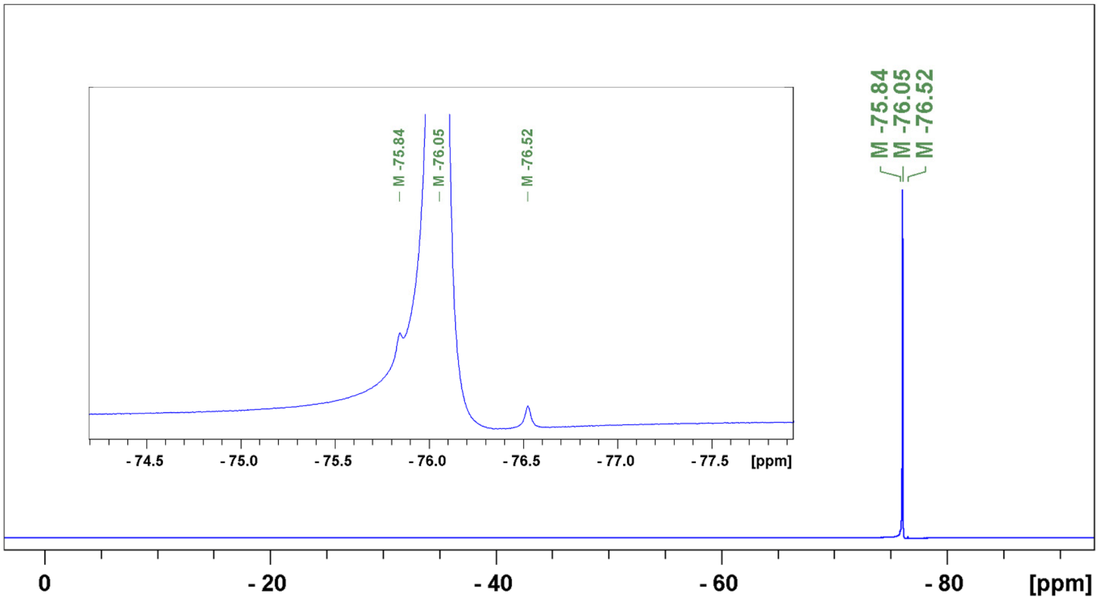

Supplementary Figure 3. ^{19}F-NMR spectrum of **1** in acetone-d_6 (282 MHz) with zoom as inset. We have replaced Supplementary Fig. 3 with this one.

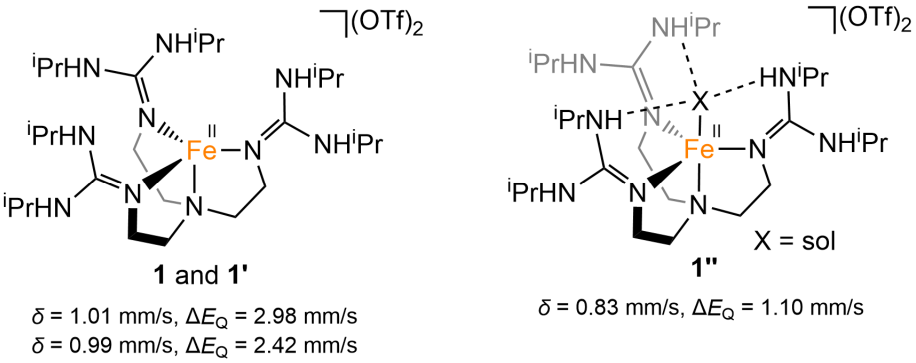

Supplementary Figure 15. Schematic representations of **1**, **1'** and **1''**.

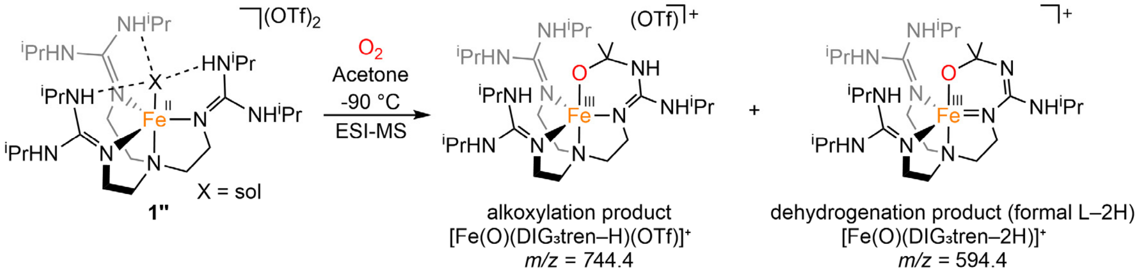

Supplementary Figure 16. Decomposition products of the reaction of **1''** with O_2 detected by ESI-MS.

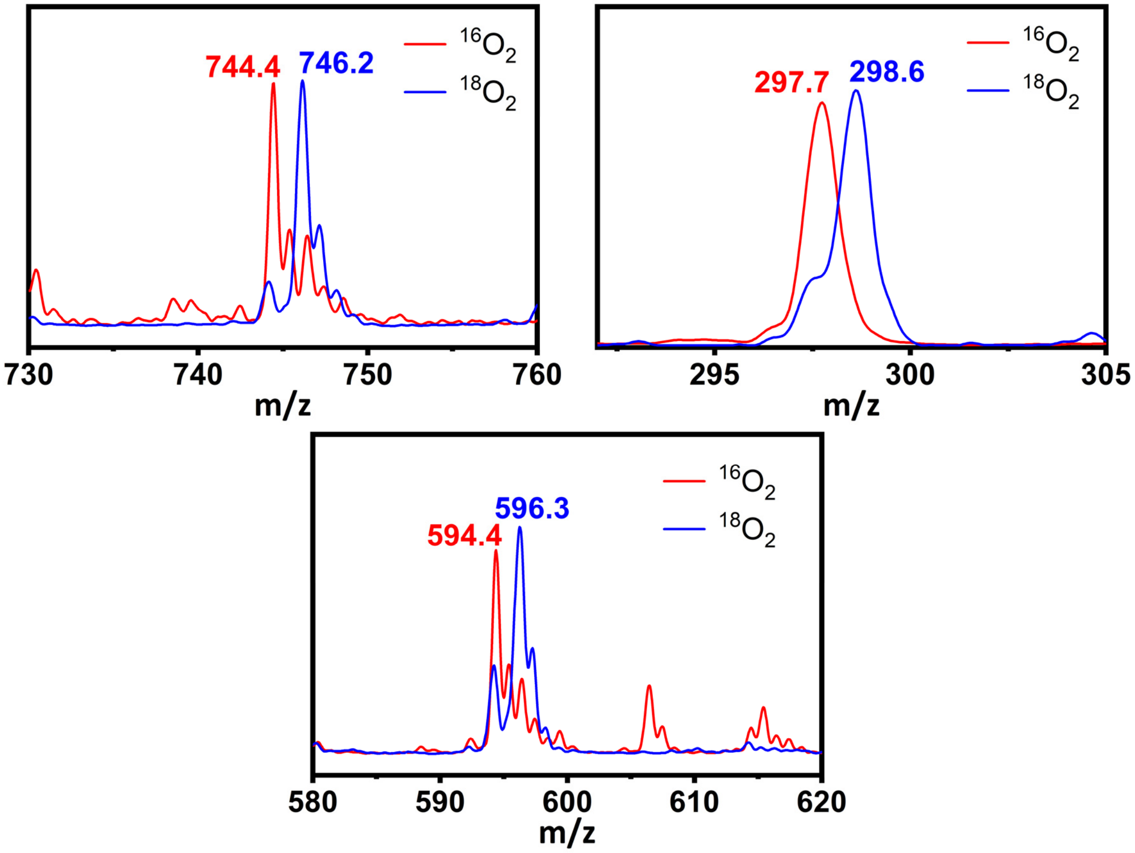

Supplementary Figure 17. ESI-MS spectra of reaction products of **1''** with $^{16}O_2$ (red) and $^{18}O_2$ (blue) showing the peaks assigned to $[Fe(O)(DIG_3tren{-}H)(OTf)]^+$ top left, $[Fe(O)(DIG_3tren{-}H)]^{2+}$ top right and $[Fe(O)(DIG_3tren{-}2H)]^+$ bottom.

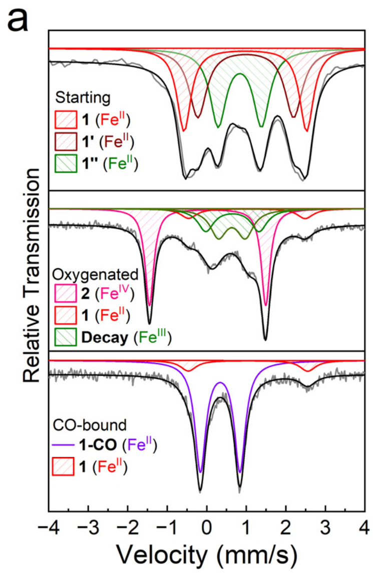

Figure 3a. Zero-field Mössbauer spectra of **1**, **2** and **1-CO** in acetone at −90 °C. Top: Zero-field Mössbauer spectrum of frozen acetone solution of **1** at 14 K (grey line: experimental spectrum; black line: simulated spectrum; red line: **1** with δ = 1.01 mm/s, ΔE_Q = 2.98 mm/s, Γ = 0.42 mm/s, 29%; brown line: **1'** with δ = 0.99 mm/s, ΔE_Q = 2.42 mm/s, Γ = 0.50 mm/s, 30%; green line: **1''** with δ = 0.83 mm/s, ΔE_Q = 1.10 mm/s, Γ = 0.49 mm/s, 41%); Middle: Zero-field Mössbauer spectrum of oxygenated acetone solution of ^{57}Fe enriched **1/1-OTf** at 14 K (grey line: experimental spectrum; black line: simulated spectrum; green lines: Fe(III) decay with δ = 0.65 mm/s, ΔE_Q = 1.35 mm/s, Γ = 0.50 mm/s, 20% and δ = 0.63 mm/s, ΔE_Q = 0.68 mm/s, Γ = 0.50 mm/s, 25%; pink line: **2** with δ = 0.01 mm/s, ΔE_Q = 2.94 mm/s, Γ = 0.26 mm/s, 46%; red line: **1**, Γ = 0.50 mm/s, 9%). Bottom: Zero-field Mössbauer spectrum of a CO flushed acetone solution of ^{57}Fe enriched **1/1-OTf** at 14 K (grey line: experimental spectrum; black line: simulated spectrum; purple line: **1-CO** with δ = 0.34 mm/s, ΔE_Q = 1.00 mm/s, Γ = 0.33 mm/s, 88%; red line: **1**, Γ = 0.50 mm/s, 12%).

3. It should be better to present a scheme showing the formation of complex 3 from 2 with PPh3.

Response: We have now added the requested scheme in the main text

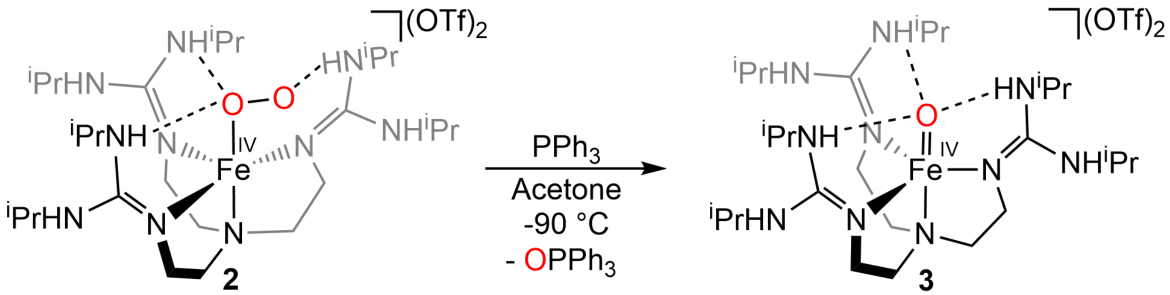

Figure 6. Reaction of **2** with triphenylphosphine in acetone at −90 °C forming complex **3** and triphenylphosphine oxide.

4. According to Supp. Fig 24, the decomposition of complex 3 seems 2-step reaction. What is the final product of this reaction? Based on that, the reaction mechanism should be discussed.

Response. The kinetic trace shown in Supplementary Figure 24 (now Supplementary Figure 29) indeed displays a two-step decay behavior. The electrospray ionization mass spectrum (ESI-MS) recorded after the self-decay of **3** shows signals at *m/z* = 744.3 and *m/z* = 297.7, which match the formulations $[Fe(O)(DIG_3tren{-}H)(OTf)]^+$ and $[Fe(O)(DIG_3tren{-}H)]^{2+}$ (calcd. *m/z* = 744.37 and 297.71), which are both lighter by one hydrogen atom relative to **3**. Both signals shift correspondingly when using isotope labeled $^sPhI^{18}O$, confirming that both the ions originate from **3**. Similar behavior has been reported for the iron(IV)-oxido complex $[Fe(O)(TMG_3tren)](OTf)_2$, where intramolecular hydrogen-atom abstraction (HAA) gives rise to the

alkoxylated (−1 H) decay product.[3] The detection of the species lighter by one hydrogen atom during the decay of **3** may therefore indicate a similar decay pathway leading to the formation of the alkoxylated product. Thus although, the -NH group is involved in the decay pathway (as evident from -NH/-ND KIE), one of the carbon atoms in the secondary coordination sphere of **1** gets eventually oxygenated. Thus, the decay process may involve a proton coupled electron transfer mechanism involving the -N-H, and $(CH_3)_2C$-H moieties, giving rise to the alkoxylated product, as shown below. This may explain the complicated kinetics observed for the decay of **3**. We have now included this discussion in the main text.

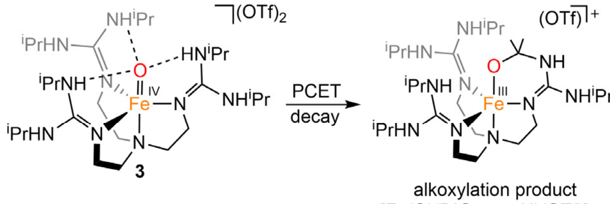

Supplementary Figure 31. Scheme showing the decay of **3** to the alkoxylated product.

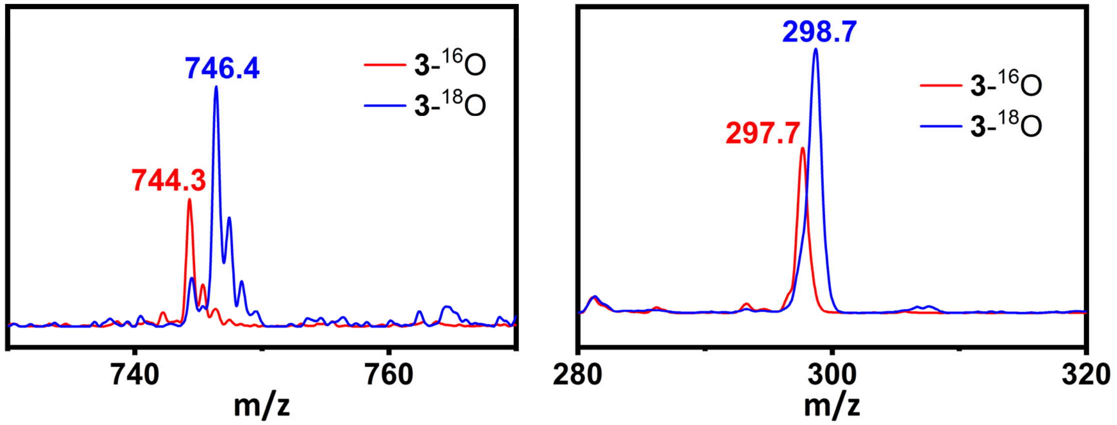

Supplementary Figure 30. ESI-MS spectrum of decay of **3** generated with ^{s}PhI^{16}O (red) and ^{s}PhI^{18}O (blue) showing the peaks assigned to $[Fe(O)(DIG_3tren-H)(OTf)]^+$ left and $[Fe(O)(DIG_3tren-H)]^{2+}$ right.

5. The decay of complex 3 involves no hydrogen tunnelling, considering the ratio of the half lifetimes of 3 and 3-d6 in CH3CN. Why is the hydrogen tunneling not involved in the HAT reaction from hydrogen-bonded N-H to the oxo ligand in complex 3? The N-H bond dissociation energy may be ~92 kcal/mol or so.

Response. We note that the kinetics in Supplementary Fig. 24 (now Supplementary Figure 29) were measured in acetone/acetone-d_6 (not CH_3CN). The decay of **3**/**3-*d*$_6$** shows only a modest isotope effect (KIE ≈ 2), fully consistent with a classical, over-the-barrier process rather than hydrogen tunneling, which typically manifests as very large KIEs (often >10-50). Although DFT gives short N–H···O distances of ca. 1.76 Å, indicating strong intramolecular hydrogen bonding, such preorganization does not always imply a narrow, one-dimensional H-transfer barrier. In fact, studies by Shaik, Borovik and co-workers have shown that N−H to oxo transfers in basic Fe-oxo systems fall in the PCET/PT–ET regime, involving substantial charge transfer and heavy-atom reorganization rather than symmetric H-atom tunneling.[4] Likewise, tunneling in nonheme Fe(IV)=O chemistry has been observed usually for classical C−H HAT pathways with highly curved, narrow transition states; these conditions do not apply to the present intramolecular N−H transfer.[5] The two-step decay profile and solvent-dependent rates observed here further indicate reorganization-limited, solvent-coupled kinetics, which are incompatible with a tunneling-dominated mechanism. Notably, the slower decay of **3** in acetone-d_6 (Supplementary Fig. 29, red trace) compared to that in acetone, further supports solvent-modulated barrier control rather than isotope-driven tunneling. We therefore conclude that the decay of **3** proceeds via a classical, non-tunneling PCET-type H-transfer pathway.

6. In the CO binding, how does the hydrogen bonding or the N-H moieties contribute to or disturb the CO coordination? In the hemoglobin case, the N-H of a distal imidazole causes steric hindrance to disfavor the linear binding mode of CO. Is it the same as in the hemerythrin case? The authors need to provide at least a

calculated structure of the proposed Fe(II)-CO complex to clarify the structure.

Response: From the calculated geometry of the complex $[Fe^{II}(CO)(DIG_3tren)]^{2+}$ with three H-bonding interactions (please see the attached figure below), we can see that the H-bonding interaction is weak (the (N)H-O distances are *ca.* 2.3-2.4 angstrom). This weak H-bonding interaction doesn't disturb the CO coordination too much and the Fe-C-O alignment keeps linear. We have added this discussion in the main text and also included the DFT calculated structure of **1-CO**.

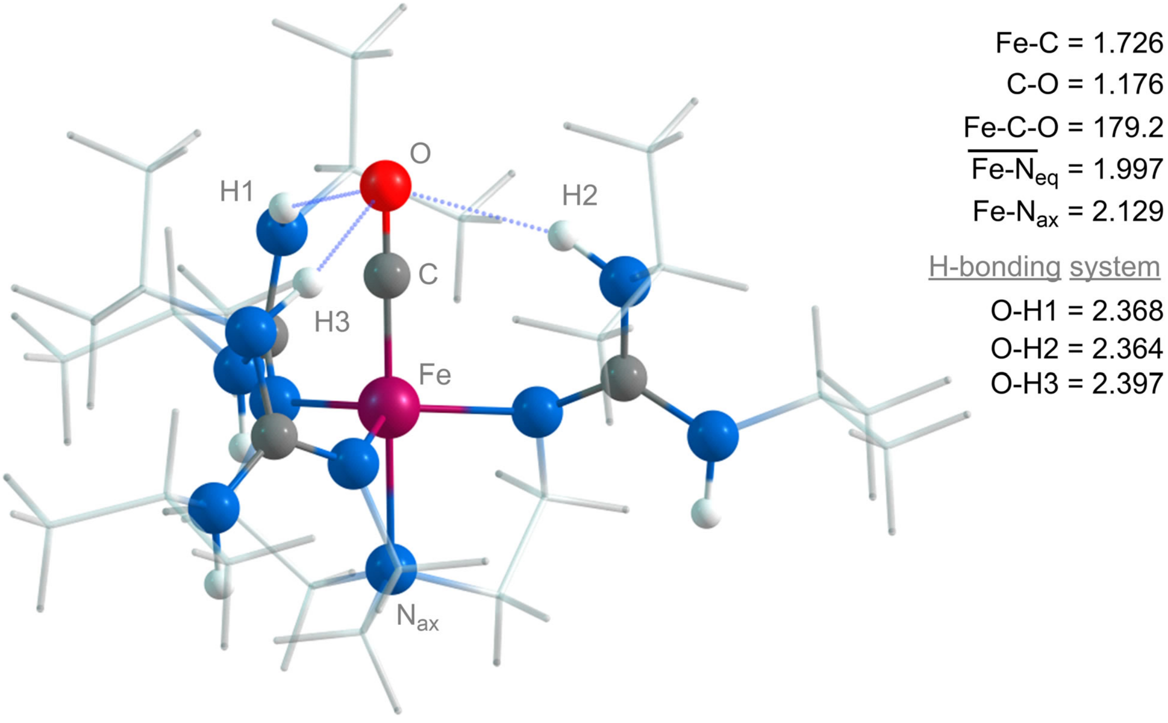

^{3}**1-CO**

Supplementary Figure 35. Calculated geometric information of the complex $[Fe^{II}(CO)(DIG_3tren)]^{2+}$ on the ground triplet (*S* = 1) spin state with three H-bonding interactions. Bond lengths are in Å units and angles are in ° unit.

7. The CO vibration energy in 1-CO should be compared with that of CO-bound hemerythrin rather than that of CO-bound hemoglobin.

Response. We appreciate the reviewer's comment. However, to the best of our knowledge, no CO-bound hemerythrin species have been reported in the literature. Hemerythrin does not form a stable CO adduct, and therefore no experimental CO stretching frequencies are available for comparison. In contrast, hemoglobin forms a well-characterized CO adduct with known *v*(CO) value, providing a meaningful experimental reference for our complex **1-CO**. For this reason, we compared the CO vibration energy of **1-CO** with that of CO-bound hemoglobin rather than hemerythrin.

8. Thermodynamic parameters should be reported for the O2 binding to argue the stability of the peroxo complex based on the van't Hoff equation.

Response. We now report the thermodynamic parameters for O_2 binding, derived from a van't Hoff analysis. Because intermediate **2** cannot be generated in a stable manner at temperatures above −80 °C, the equilibrium constants could only be measured within a narrow temperature window (−95 to −80 °C in acetone). Within this accessible temperature range, the van't Hoff plot (based on four K values at four temperatures; Supplementary Figure 43) yields $\Delta H°$ = −10.66 ± 0.37 kJ mol^{-1} and $\Delta S°$ = −16.5 ± 2.0 J mol^{-1} K^{-1}. The negative values of $\Delta H°$ and $\Delta S°$ are consistent with the binding of O_2 only at low temperature.

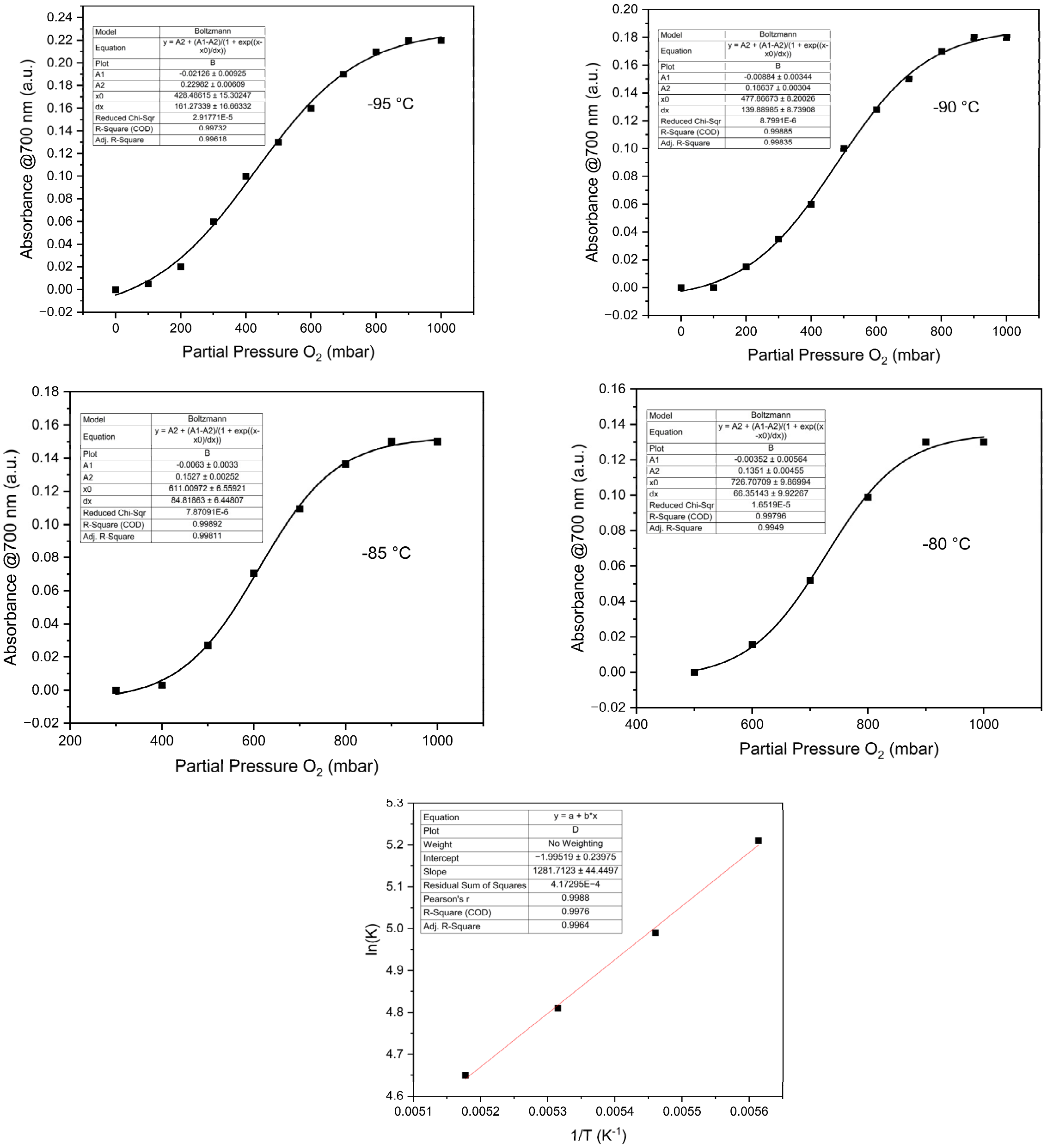

Supplementary Figure 43. Top and middle: Measured absorbance of **2** at 700 nm in acetone at −95 °C (top left), −90 °C (top right), −85 °C (bottom left), −80 °C (bottom right) using gas mixtures (O_2/Ar) with different partial pressures (total pressure 1000 mbar). Bottom: van't Hoff plot with the four equilibrium constants obtained ($K_{-80\,°C}$ = 104.88 M^{-1}; $K_{-85\,°C}$ = 122.36 M^{-1}; $K_{-90\,°C}$ = 146.84 M^{-1}; $K_{-95\,°C}$ = 183.55 M^{-1}; see SI for details).

9. On page 4, in the left column, the sentence "The calculated Fe-O distance at 1.802 Å, and O-O bonds at 2.391 Å, and 1.391 Å, respectively, are in good agreement with the experiment." is not clear and should be revised.

Response: The sentence was revised as "The calculated Fe-O (1.802 Å) and O-O (2.391 Å, and 1.391 Å) distances of the end-on peroxido complexes are in good agreement with the experiment."

10. In Supplementary figs 15 – 16, explanation of values in the figures should be added in each caption. Also,

correct the disorder of numbers.

Response: The explanation of values in Fig. S15-S16 and the order of numbers were added in each figure caption as the description "Bond lengths are in Å units and the order of numbers is a sequence of values for the singlet/triplet/quintet spin states."

11. Elemental analysis data should be provided for all new complexes prepared and reported herein.

Response. Elemental analyses for all complexes reported in the paper are now provided in the supporting information. Furthermore, additional synthesis procedures for the additional complexes are included.
Elemental analysis calc. for $C_{27}H_{60}B_2F_8FeN_{10}$: C 42.99, H 8.02, N 18.57; found: C 43.12, H 8.04, N 18.51.
Elemental analysis calc. for $C_{75}H_{100}B_2FeN_{10}$: C 73.89, H 8.27, N 11.49; found: C 74.18, H 8.29, N 11.45.
Elemental analysis calc. for $C_{31}H_{60}F_{12}FeN_{12}O_8S_4$: C 32.63, H 5.30, N 14.73, S 11.24; found: C 32.75, H 5.32, N 14.69, S 11.28.
Elemental analysis calc. for $C_{27}H_{60}Br_2N_{10}Zn$: C 43.24, H 8.06, N 18.68; found: C 43.40, H 8.09, N 18.62.

Reviewer #2 (Remarks to the Author)

In this manuscript, Ray et al. report a novel mononuclear iron complex supported by a guanidine-based ligand, which plays a critical role in providing steric protection and modulating the secondary coordination sphere. Thanks to this design, a rare iron(IV)–peroxido intermediate was observed upon binding of the Fe(II) complex with O_2, and the process was shown to be reversible. The steric shielding and hydrogen-bonding interactions afforded by the secondary coordination sphere facilitate two-electron transfer from the Fe(II) center to the bound dioxygen unit, while preventing irreversible O–O bond cleavage and formation of Fe–O–Fe dimeric species. Notably, this nonheme model exhibits a higher affinity for O_2 than for CO, contrasting with the behavior of hemoglobin. I find this work highly interesting; however, several major issues need to be clarified. I would therefore be willing to recommend the manuscript for publication in Nature Communications after major revisions.please find the foloowing issues：

1. Initial Fe(II) complex have two forms of existence, but only four-coordinate form can be crystallized. Thus, it is possible for separating these two species in solid state. Theoretically, authors could provide the separated characterization of the four-coordinate [FeII(DIG3tren)]2+.

Response. We have re-measured the Mössbauer spectrum of **1**/**1-OTf** in acetone (see response to Reviewer 1, point 2). While the doublet corresponding to **1** persists, one additional four coordinate Fe(II) species (**1'**) and one additional five coordinate Fe(II) species (**1''**) appear in solution. We believe that the triflate ligand dissociates from the axial site in solution, generating a mixture of four and five coordinate Fe(II) species. This is supported by the ^{19}F-NMR spectrum of **1**/**1-OTf** in acetone (Supplementary Figure 3), in which no signal appears in the chemical-shift range expected for bound triflate. Instead, two small shoulders surround the main resonance of free triflate. We attribute these features to the presence of multiple species in acetone solution, most likely arising from different hydrogen-bonding environments of the triflate anions. Such speciation is fully consistent with the intrinsic flexibility of the DIG_3tren ligand, whose arms can alternately shield or open the axial position giving rise to different isomeric forms, as also demonstrated previously (Response Figure R1). As suggested by the reviewer, we could indeed separate four coordinate **1** in the solid state; the Mössbauer spectrum shows a doublet with δ = 1.01 mm/s, ΔE_Q = 2.98 mm/s (Supplementary Figure 10). The unequal doublet intensities presumably arise from crystal-dependent effects (including line broadening and slight site heterogeneity).[6] However, dissolving crystalline **1** in acetone again led to the appearance of multiple Fe(II) signals corresponding to the formation of different isomeric species (Figure 3a).

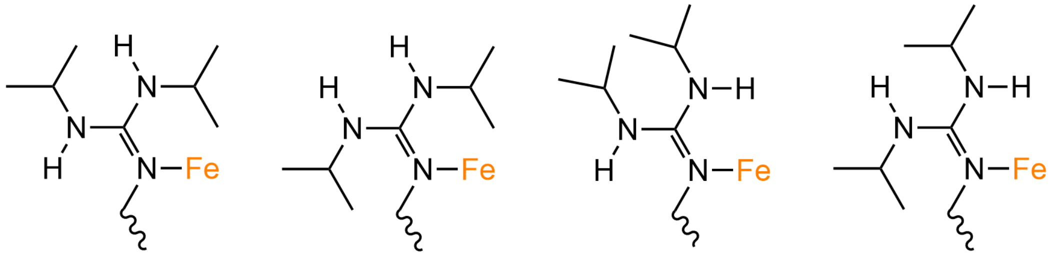

Response Figure R1. Conformations of ligand arms of DIG$_3$tren as reported by Scarrow and Coworkers. [7]

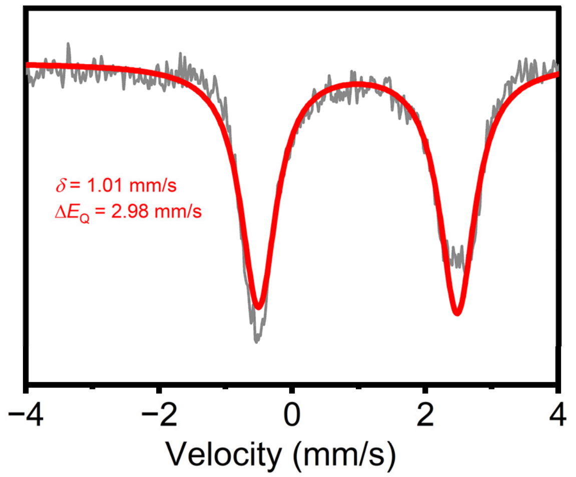

Supplementary Figure 10. Zero-field Mössbauer spectrum of crystalline **1** at 14 K (grey line: experimental spectrum; red line: simulated spectrum with δ = 1.01 mm/s, ΔE_Q = 2.98 mm/s, Γ = 0.64 mm/s).

2. I suggest that authors could use the non-coordinating Fe(II) salts, such as Fe(CH3CN)4(BF4)2, to prepare the pure four-coordinate [FeII(DIG3tren)]2+ complex. In this way, interference from 1-OTf can be eliminated.

Response. To address the reviewer's suggestion that the use of non-coordinating Fe(II) salts might eliminate interference from **1-OTf** (and species derived from it in solution; see Response to Reviewer 1, point 2), we prepared the corresponding complexes [Fe(DIG$_3$tren)](BF$_4$)$_2$ (**1-BF$_4$**), [Fe(DIG$_3$tren)](BPh$_4$)$_2$ (**1-BPh$_4$**), and [Fe(DIG$_3$tren)](NTf$_2$)$_2$ (**1-NTf$_2$**). The solid-state Mössbauer spectrum of **1-BF$_4$** shows a single Fe(II) species with parameters very similar to those of **1** (Supplementary Figure 38, left). Notably, however, **1-BF$_4$** does not form intermediate **2** upon reaction with O_2, as demonstrated by UV/Vis spectroscopy (Supplementary Figure 38, right). We attribute this to the ability of BF_4^- to engage in significantly stronger intermolecular hydrogen bonding with the DIG$_3$tren ligand than triflate, thereby disrupting the secondary-sphere preorganization that stabilizes the Fe(IV)-peroxo intermediate **2**. We bleive that the ability to reversibly bind O_2 is unique for four coordinate **1** in presence of triflate ion. We have now elaborated this discussion in the conclusion

"The ability to reversibly bind O_2 is unique for four coordinate **1** in presence of OTf anion, where all the DIG arms exist in the closed conformation (Fig. 2). The stabilization of the Fe(IV)-peroxido intermediate, however, warrants the conversion of the DIG arms into the open conformation (Fig. 4, Fig. 7). Accordingly, O_2 binding to the Fe(II) center and the isomerization of the DIG arms must occur at comparable rates, so that the transient Fe(III)-superoxido intermediate is converted to the more stable Fe(IV)-peroxido form via a H-bonding mediated electron-transfer process. Significant increase in the O_2 binding rate relative to the ligand reorganization rate (for example in the open conformation of one or more DIG units of the DIG$_3$tren ligand), will allow the oxidative decomposition of ligand by the transient Fe(III)-superoxido intermediate, and complex **2** will be inaccessible. To further probe the role of the counterion, we examined complexes

bearing weakly coordinating anions. While the solid-state Mössbauer spectrum of **1-BF$_4$** shows a single Fe(II) species closely resembling **1**, this complex does not form intermediate **2** upon exposure to O_2, as evidenced by UV/Vis spectroscopy (Supplementary Fig. 38). This behaviour is attributed to stronger hydrogen-bonding interactions between BF_4^- and the DIG$_3$tren ligand, which disrupt the secondary-sphere preorganization required for peroxido stabilization (Fig. 4). In contrast, complexes containing truly non-coordinating anions (**1-BPh$_4$** and **1-NTf$_2$**) display multiple Fe(II) environments in the solid state, consistent with the increased conformational flexibility of the ligand scaffold, and likewise fail to form **2** (Supplementary Figs. 39-42). Collectively, these results highlight the unique role of triflate anion: it is sufficiently weakly coordinating to leave the axial site accessible for O_2, yet capable of preorganizing the hydrogen-bonding network in a manner that selectively stabilizes the iron(IV)-peroxido intermediate (Fig. 4). Deuteration of the N-H group in the DIG moiety led to the formation of **2** in a reduced yield of 5%, giving a N-D/N-H kinetic isotope effect of 0.11. This may further corroborate the motion of hydrogen/deuterium atoms during the formation of **2;** the rate of d_6-DIG$_3$tren rearrangement involving heavier N-D moieties becomes slower relative to O_2 binding, yielding **2** in significantly reduced yields."

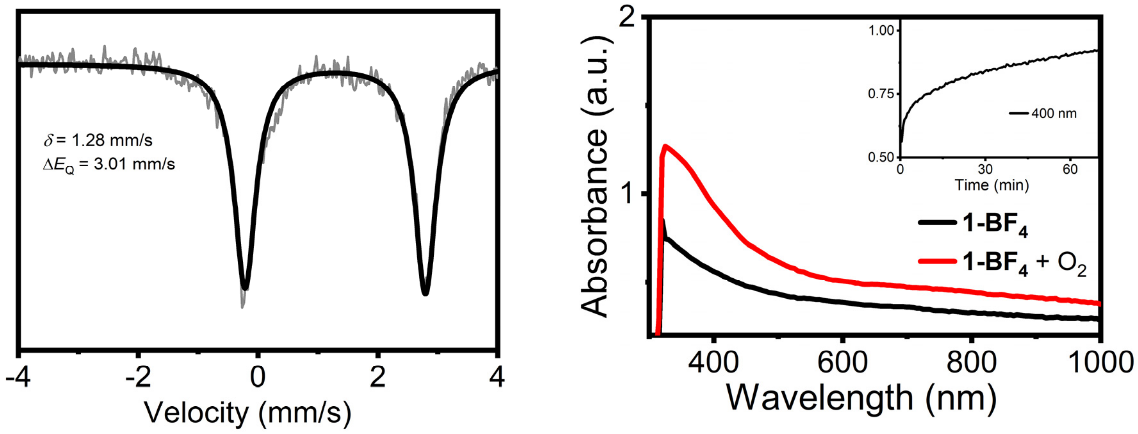

Supplementary Figure 38. Zero-field Mössbauer spectrum of **1-BF$_4$** at 14 K (left; grey line: experimental spectrum; black line: simulated spectrum with δ = 1.28 mm/s, ΔE_Q = 3.01 mm/s) and UV/Vis spectrum of **1-BF$_4$** and the reaction of **1-BF$_4$** with O_2 at −90 °C in acetone with time trace as inset (right).

Furthermore, the solid-state Mössbauer spectrum of **1-BPh$_4$,** which contains a truly non-coordinating and non-H-bonding BPh_4- anion, exhibits two component spectra (Supplementary Figure 40, left), consistent with multiple geometries or conformational states in the solid. This complex also fails to form **2** in its reaction with O_2 (Supplementary Figure 40, right).

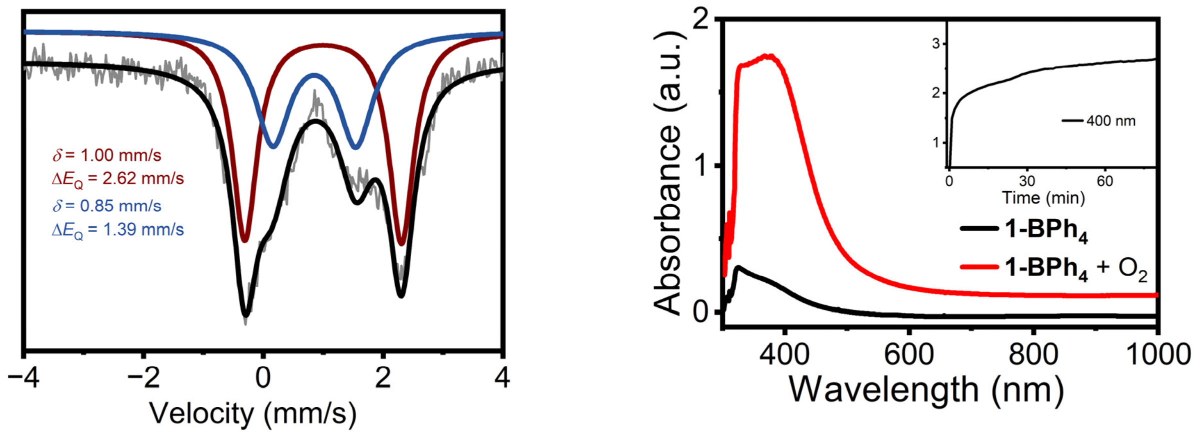

Supplementary Figure 40. Zero-field Mössbauer spectrum of **1-BPh₄** at 14 K (left; grey line: experimental spectrum; black line: simulated spectrum; dark red line with δ = 1.00 mm/s, ΔE_Q = 2.62 mm/s; blue line with δ = 0.85 mm/s, ΔE_Q = 1.39 mm/s) and UV/Vis spectrum of **1-BPh₄** and the reaction of **1-BPh₄** with O_2 at −90 °C in acetone with time trace as inset (right).

Finally, **1-NTf₂** featuring a bis(trifluoromethanesulfonyl)azanide (NTF_2^-) anion that is sterically more hindered than triflate and less prone to coordination was tried. Here again, the spectrum of **1-NTf₂** reveals multiple Fe(II) environments (Supplementary Figure 42). Owing to the multiple ways in which NTf_2^- can engage in hydrogen-bonding arrangements that differ from triflate, no formation of intermediate **2** is observed.

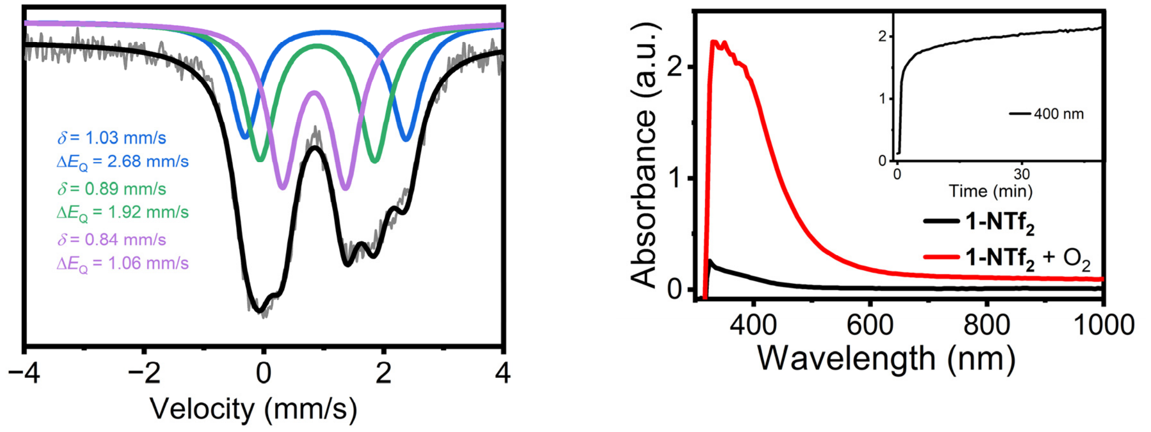

Supplementary Figure 42. Zero-field Mössbauer spectrum of **1-NTf₂** at 14 K (left; grey line: experimental spectrum; black line: simulated spectrum; blue line with δ = 1.03 mm/s, ΔE_Q = 2.68 mm/s; green line with δ = 0.89 mm/s, ΔE_Q = 1.92 mm/s; purple line with δ = 0.84 mm/s, ΔE_Q = 1.06 mm/s) and UV/Vis spectrum of **1-NTf₂** and the reaction of **1-NTf₂** with O_2 at −90 °C in acetone with time trace as inset (right).

Across all three cases, it becomes clear that the triflate anions in **1** play a unique role: on one-hand, they are weak ligands so that a significant amount of **1** with non-coordinating OTf^- exists in solution. At the same-time they pre-orient and organize the intermolecular hydrogen-bonding network in a way that is essential

for stabilizing the Fe(IV)-peroxo intermediate **2**. Thus, a fine tuning of intermolecular H-bonding strength and sterics of the anion may play a vital role in the stabilization of **2**; presently, it works only with the triflate anion: When a solution of **2** is flushed with argon, it cleanly reverts to **1**, regenerating only the isomer with the large quadrupole splitting (δ = 1.01 mm/s, ΔE_Q = 2.98 mm/s) corresponding to four coordinate iron complex. Mössbauer parameters of this magnitude are observed exclusively in the presence of triflate or BF_4^- anions (δ = 1.28 mm/s, ΔE_Q = 3.01 mm/s). In the case of BF_4^-, however, strong hydrogen bonding between the DIG_3tren ligand and the fluorine atoms of the anion immobilizes the N–H groups and prevents the reorganization of the hydrogen-bonding network toward the bound O_2 unit that is required to stabilize intermediate **2**. In contrast, all other forms of **1** observed in acetone, as well as those obtained with weakly interacting anions (BPh_4^-, NTf_2^-), exhibit isomers with substantially smaller quadrupole splittings. Upon exposure to O_2, these species uniformly convert to iron(III) decay products. This behavior reflects the absence of the preorganized hydrogen-bonding arrangement necessary to promote the two-electron reduction of dioxygen and the formation of **2**.

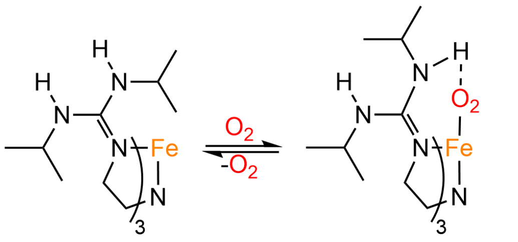

Figure 4. Preorganization of the N–H groups in **1** enables hydrogen-bond-assisted stabilization of the peroxo intermediate **2** upon O_2 binding, whereas altered hydrogen-bonding environments in acetone prevent productive O_2 reduction and instead leads to Fe(III) decay products.

3. If Fe(II) complex is a mixture in solution, it should show two different 19F peaks in its 19F-NMR spectrum, but there is only one 19F peaks in Supplementary Figure 3.

Response. The originally reported ^{19}F-NMR spectrum was recorded in CD_3CN, where solvent coordination at the axial position suppresses the formation of a bound triflate species; accordingly, only a single resonance for free triflate is observed. To allow direct comparison with the other spectroscopic data, we re-measured the ^{19}F-NMR spectrum in acetone-d_6 (Supplementary Figure 3). Again, no signal appears in the chemical-shift range expected for bound triflate. Instead, two small shoulders surround the main resonance of free triflate appear. We attribute these features to the presence of multiple species in acetone solution, most likely arising from different hydrogen-bonding environments of the triflate anions. This interpretation is fully consistent with the Mössbauer spectrum of **1/1-OTf** in acetone, which also indicates the coexistence of several species and the absence of **1-OTf** in solution (see response to Reviewer 1, point 2).

4. Is there a chemical equilibrium between species 1 and 1-OTf? How about the equilibrium constant?

Response. In frozen acetone solution, the Mössbauer spectrum of **1** displays three subspectra (**1**, **1'** and **1''**, Figure 3a). We attribute this behavior to a distribution of Fe(II) environments arising from the flexible ligand framework and multiple hydrogen-bonding arrangements involving solvent and outer-sphere triflate giving rise to four and five coordinated iron(II) species.

Consistent with this, the ^{19}F-NMR spectrum (Supplementary Figure 3) of the triflate region in solution shows signals characteristic of free/outer-sphere triflate, without additional well-resolved resonances that would indicate a distinct, strongly bound inner-sphere **1-OTf** species (see Response to Reviewer 1, Point 2). Taken together, these observations suggest that if an inner-sphere **1-OTf** species is present in solution, it constitutes at most a minor and/or rapidly exchanging component of a broader distribution of hydrogen-

bonded environments, rather than a dominant, discrete species in equilibrium with **1**.

We have therefore restricted our discussion of **1/1-OTf** in solid state and **1**, **1'** and **1''** in solution to a qualitative description and have not attempted to derive an equilibrium constant.

5. In manuscript, authors stated: in the presence of oxygen, complex 1-OTf performs a fast irreversible electron transfer process to yield high-spin iron(III) decay products". Is it an intermolecular electron transfer between 1-OTf and oxygen molecule? What is the oxidation potential of 1-OTf.

Response. The most plausible scenario is that O_2 first coordinates to the Fe(II) center of the species present in the solution, generating a highly reactive and short-lived Fe(III)-superoxido intermediate. This intermediate rapidly undergoes intramolecular hydrogen atom abstraction on the DIG_3tren ligand, leading to irreversible formation of the Fe(III) decay products for the complex species lacking the preorganization of hydrogen bonds necessary for two electron reduction of dioxygen (see Response to Reviewer 1, point 2). Because multiple Fe(II) species are present in solution for **1**, it is not feasible to assign a meaningful oxidation potential to a single, well-defined entity. For reference, the cyclic voltammogram of **1** measured in acetone under argon shows two redox couples at $E_1/_2$ = −0.61 V and +0.14 V, respectively. This spectrum is not included in the main text or SI, as we are not confident about the assignment of the redox couples.

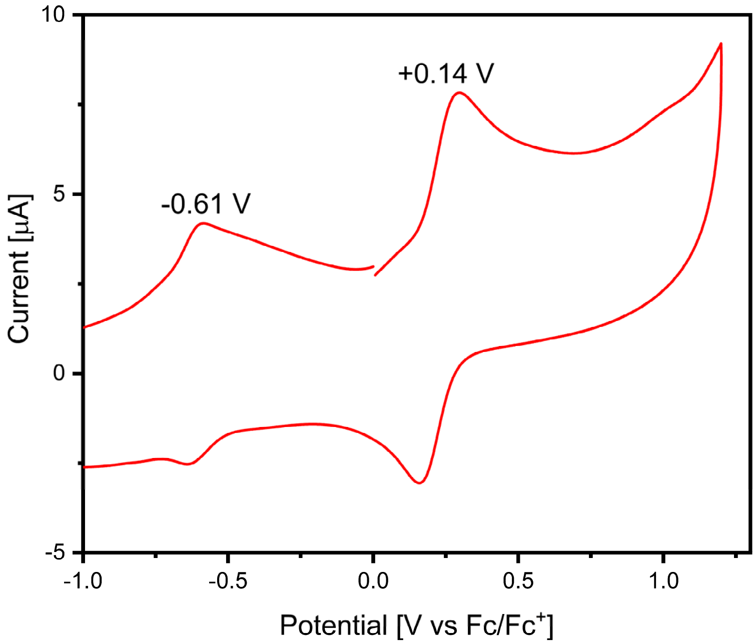

Response Figure R2. CV of **1** (1 mM) in acetone at room temperature using Bu_4NPF_6 as conducting salt.

6. In Fig.3a, Mössbauer spectrum of a CO flushed acetone solution of 1/1-OTf shows only two species, 1 and 1-CO, why did 1-OTf disappear? According to author statement, 1-OTf cannot bind with CO molecule and remains unchanged in the solution. So, Is it possible that the species (δ = 0.97 mm/s and ΔEQ = 1.67 mm/s) in Mössbauer might not be 1-OTf, rather than some impurity?

Response. We did not state that **1-OTf** cannot bind CO; rather, we understand that the uncertainty regarding the O_2-reaction pathway may have led to this assumption. In acetone solution, **1''** reacts with O_2, leading to irreversible oxidation to Fe(III) and concomitant ligand oxidation (see response to Reviewer 1, point 2). In contrast, CO binds at the axial site without any Fe-CO electron transfer, thereby preserving the Fe(II) oxidation state. Under these conditions, all solution Fe(II) species convert cleanly to **1-CO**. The observation that multiple initial species collapse into a single Mössbauer-detectable Fe(II)-CO product

supports our interpretation that they represent different isomers/rotamers arising from the flexibility of the DIG_3tren ligand and its variable hydrogen-bonding interactions with solvent and triflate. We added this discussion to the main text. In particular, we have tried toe xplain this in the conclusion

"Nevertheless, the near-quantitative formation of a single Fe(II)–CO adduct upon exposure to CO demonstrates that all Fe(II) centers in acetone solution (corresponding to **1**, **1'**, and **1''**; Fig. S14) are accessible for O_2/CO binding. The CO coordination proceeds without electron transfer, preserving the Fe(II) oxidation state and preventing ligand oxidation; consequently, all conformers converge cleanly to **1-CO** (Fig. 3a, bottom). In contrast, the productive two-electron reduction of O_2 occurs only for a specific isomer in which all N–H hydrogen-bond donors stabilize the bound O_2^{2-} species, thereby enabling formation of intermediate **2**. For other isomers lacking this preorganized hydrogen-bonding environment, O_2 binding instead results in a highly reactive Fe(III)–superoxido species, which rapidly induces ligand oxidation, resulting in reduced yields for **2**."

Importantly, **1-OTf** in solid and **1'** and **1''** in solution are not impurities: the elemental analysis confirms the purity of the isolated material. The multiplicity of species in solid/solution instead originates from the intrinsic flexibility of the DIG_3tren framework, which allows the axial site to be alternately accessible or shielded, and permits several distinct hydrogen-bonding environments for the anions or solvent resulting in four and five coordinated iron(II) species.

7. Could authors provide more structural information about the decay species (Fe(III) species), such as using MS method? Is it stable in room temperature? Could it be Fe(III)-superoxide intermediate?

Response. For structural information on the decay species, please see our response to Reviewer 1, point 2. The Fe(III) decay products are stable at room temperature, although all attempts to crystallize any of these species have so far been unsuccessful. Importantly, the multiple decay species observed are not Fe(III)-superoxide intermediates. Such an Fe(III)-superoxide species would be highly reactive and intrinsically unstable (as supported by our DFT calculations) and is therefore expected to exist only as a transient intermediate that precedes formation of the observed Fe(III) decay products.

8. I suggest that EPR measurement of the possible Fe(IV)-peroxo intermediate can be supplemented, to further exclude the potential Fe(III)-superoxide form.

Response. Please note that resonance Raman investigation shows an O-O vibration at 823 cm^{-1} of **2**, which is consistent with the peroxide assignment. Furthermore, we believe that EPR spectroscopy is not suitable to distinguish between an Fe(IV)–peroxide and an Fe(III)–superoxide formulation. Both species are expected to be EPR silent in the perpendicular mode X-band measurements: Fe(IV)–peroxo (S = 1) exhibits a large zero-field splitting (D ≈ 20 cm^{-1}) and integer spin, while an Fe(III)–superoxide species would involve antiferromagnetic or ferromagnetic couplings between the S = 5⁄2 Fe(III) center and the S = 1⁄2 superoxide radical, resulting in an overall integer spin state (S= 3 or 2). The recorded EPR spectrum of **2** (Response Figure R3) only displays signals characteristic of high-spin Fe(III) around g = 9.2 and g = 4.3, consistent with the presence of Fe(III) decay products formed during the generation of intermediate **2** (also detected in Mössbauer and ESI-MS measurements). The spectrum also shows a minor (<1%) signal around g = 2.0 consistent an S = 1⁄2 species.

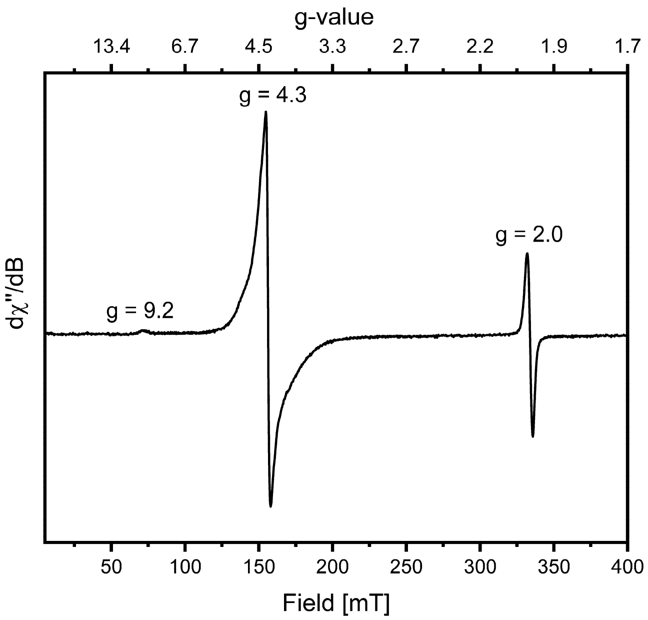

Response Figure R3. X-band EPR spectrum of **2** and decay products (1 mM) in acetone at 13 K. The S=5/2 signal correspond to high-spin Fe(III) decay products observed in the Mössbauer and ESI-MS measurements (Figure 3 and supplementary Figures 16 and 17)

9. About Fe(IV)-oxido species 3, did authors optimize its structure by DFT calculation? They can show more geometrical details of species 3 in manuscript.

Response: We present the optimized structure of the Fe(IV)-oxido species **3** (please see the attached figure below) in the SI now. The three H-bonding interactions are very strong (the (N)H-O bond lengths are ~ 1.7-1.8 angstrom.), which stretches the Fe-O bond by ~ 0.03 angstrom (1.687 Å for ^{5}**3** vs 1.655 Å for ^{5}**3’** without HB interaction.).

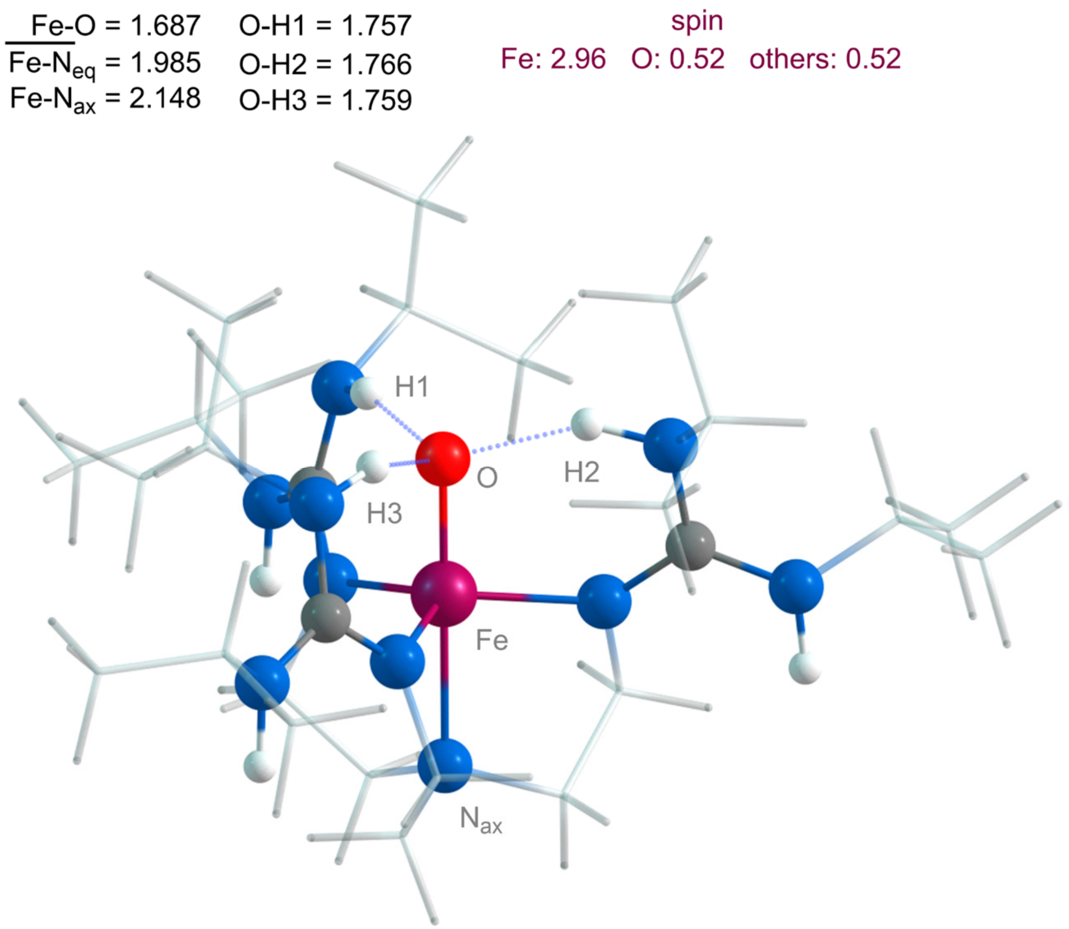

Supplementary Figure 26. The calculated geometric and spin information of the complex $[Fe^{IV}(O)(DIG_3tren)]^{2+}$ (**3**) on the ground quintet (*S* = 2) spin state with three H-bonding interactions. Bond lengths are in Å units.

10.In Supplementary Figure 27, the IR spectrum of 1 should be added for comparison. Due to the weak rRaman peak of 1-CO species, I suggest that IR spectrum of 1-CO should be supplemented to further confirming the structure of Fe-CO center, including the 13CO labeled IR spectrum.

Response. We agree that the intensity of the 1975 cm^{-1} band is very weak. We have now used the unsmoothed rRaman spectra, which clearly show that the 1975 cm^{-1} signal is above the noise level and it disappears upon decay of **1-CO** while other signals increase in intensity. The assignment to the CO stretching is justified since the frequency agrees very well with the expected values. Moreover, no other fundamentals are expected in this region. Since **1-CO** is thermally unstable and we do not have access to a low-temperature IR spectrometer, we were unable to successfully measure an IR spectrum of **1-CO**.

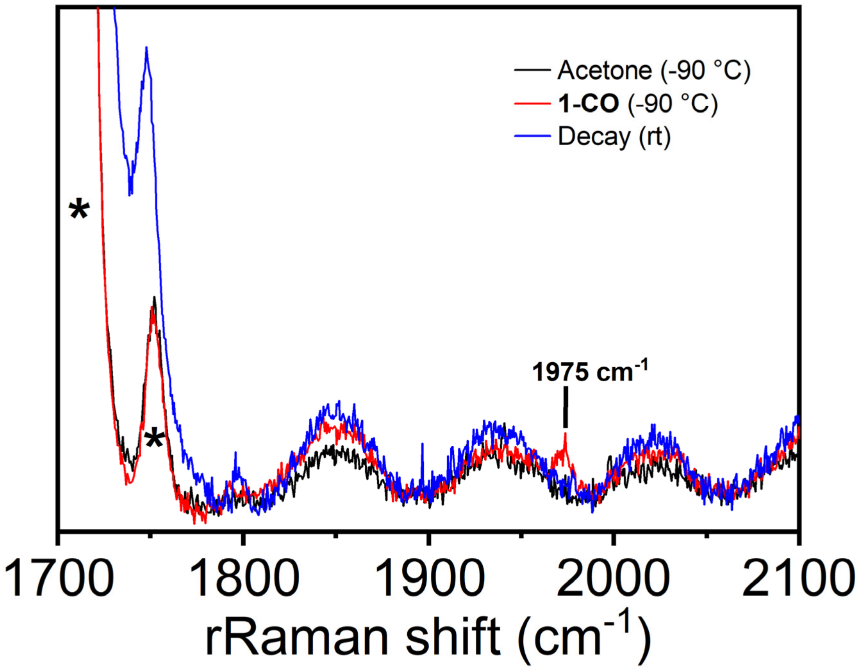

Supplementary Figure 34. rRaman spectra (λ_{exc} = 568 nm) of acetone at −90 °C (black), **1-CO** at −90 °C in acetone (red) and its decay at room temperature (blue). Solvent peaks are marked with an asterisk.

Reviewer #3 (Remarks to the Author):

The manuscript by Ray, Nam, and co-workers describes a non-heme iron complex that reversibly binds dioxygen. The authors have demonstrated that the dioxygen-bound form features an iron(IV)-peroxo species and that the complex has high affinity for dioxygen binding compared to carbon monoxide binding. This finding is interesting, as the number of nonheme iron complexes capable of reversible dioxygen binding is very small. Moreover, this complex shows exceptional discrimination for dioxygen binding versus CO. The manuscript is well written and the presentation and interpretation of the data are done at a very high level. I do, however, have one concern about the analysis, and I hope that the authors can clarify their approach.

My primary concern is in the assignment of 2 as a side-on Fe(IV)-peroxo complex. As noted by the authors, the mixed isotope experiment should allow one to distinguish between a side-on or an end-on peroxo complex. Specifically, a side-on species should have three peaks, while an end-on species should have four peaks. To my eyes, the resonance Raman data in Figure 4b have three peaks (near 792, 805, and 821 cm-1). I was somewhat surprised that these peaks are fit with five separate vibrations. In particular, the peak at 821 cm-1 is fit with three separate peaks, one at 823 cm-1 from the Fe(IV)-16O16O species, one at 817 from one of the Fe(IV)-16O18O species, and a peak from acetone-d6. It appears that the maximum for one experimental peak is not exactly 823 cm-1, as would be expected for the Fe(IV)-16O16O species. However, I wonder if other fits, with four bands instead of five, could be used to fit the data. This point is important, because, as far as I could tell, this is the decisive experiment for an end-on peroxo, and then hydrogen-bonding is inferred from this binding mode.

Response: We admit that presentation of the RR data and the description of the band fitting analysis was not sufficiently clear. We now revised this section to address the critical comments of the reviewer.

First of all, we now show the original unsmoothed spectra such that one may assess the quality of the experimental results and the fitting analysis. In the new version, we subtracted the weak solvent band (817 cm^{-1}) from the experimental spectrum of the complex generated with $^{16}O_2$/$^{18}O_2$-mixed gas. Subtraction was made on the basis of the band fitting analysis of the neat solvent spectrum that yielded the spectral parameters of this band. Visual inspection of the resultant spectrum of the $^{16}O_2$/$^{18}O_2$-complex immediately reveals that at least three bands remain in the region between 785 and 840 cm^{-1}, which are due to modes involving oxygen. This observation rules out the assignment of the complex to an Fe=O species where only two bands are expected, and leaves the option of either a side-on or end-on O-O arrangement, corresponding to three or four bands, respectively. These two options were checked by the band fitting

analysis. In both cases, the critical boundary condition was that two of the bands must be at positions observed for the complexes generated with pure $^{16}O_2$ and $^{18}O_2$, i.e. at 823 and 792 cm^{-1}, respectively. The spectral parameters of these complexes were determined in separate experiments (half width, frequency). To check the end-on configuration we used four Lorentzian functions in which a half width of 12.4 cm^{-1} was kept constant for all bands. Thus, the degrees of freedom of the fitting routine was considerably reduced to 4 frequencies and 4 intensities. As a result, we obtained a very good fit with the residuals corresponding to the noise level of the spectrum and the positions of the lowest and highest frequency bands corresponding to the values observed in the spectra of the pure $^{16}O_2$ or $^{18}O_2$ complexes. Also, the additional two bands fall in the expected range of the corresponding $^{16}O^{18}O$-complexes for an end-on configuration. We also analysed the spectrum by three Lorentzians as suggested by reviewer. In fact, a fit using three Lorentzians yielded a similar good simulation as the four-Lorentzian fit (very similar residues). However, the band positions of the pure $^{18}O_2$- and $^{16}O_2$-complexes could not be reproduced. Thus, we discard the option of a side-on arrangement.

In the revised version of the manuscript, we revised the Raman part (Figure 5a, b) and largely revised the corresponding text. We also added a new Figure in the Supporting Information and revised the description of the band fitting analysis.

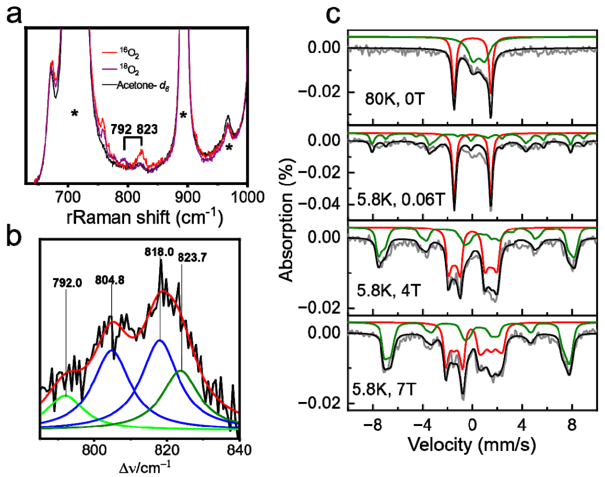

Fig. 5 | rRaman and Mössbauer characterizations of 2. **a**, rRaman spectra (−90 °C, λ_{exc} = 568 nm) of acetone-d_6 (black) and **2** prepared at −90 °C in acetone-d_6, generated by using $^{16}O_2$ (red) and $^{18}O_2$ (purple). **b**, band fitting analysis of the spectral region between 785 and 840 cm^{-1} of **2**, prepared at −90 °C in acetone-d_6 using a mixture of $^{16}O_2$, $^{18}O_2$, ^{16}O-^{18}O. The weak band of the solvent at 817 cm^{-1} was subtracted from the experimental spectrum (black trace). Four Lorentzian functions were fitted to the experimental spectrum, representing the complexes with $^{16}O_2$ (light green), $^{16/18}O_2$ and $^{18/16}O_2$ (blue), and $^{18}O_2$ (dark green). The red trace shows the cumulative fit. Details of the fitting procedure are given in the SI. **c**, Mössbauer spectra of **2** (red component) recorded at 80 K without applied magnetic field or at 5.8 K in a magnetic field of 0.06, 4 or 7 T applied parallel to the direction of the γ-rays. Green lines correspond to the Fe^{III} decay products formed during the reaction. Spin-Hamiltonian simulation parameters in SI Table 2.

It is unfortunate that the experiments using the deuterated ligand could not help in establishing hydrogen-bonding between the supporting ligand and the bound peroxo. What about other options, such as ligands that have reduced hydrogen-bonding capabilities (such as electron-withdrawing groups on the hydrogen-bond donors)? If hydrogen-bonding is important, than the lack of interactions, or fewer interactions, should influence the K for dioxygen binding.

Response. The critical role of hydrogen bonding is underscored by the observation that the TMG_3tren analogue, which lacks N–H donors, does not react with O_2 at all. Accordingly, the presence of N–H hydrogen bonds in **1** is directly linked to its ability to bind O_2 and to form intermediate **2** in the first place. Consistent with this interpretation, isotopic substitution of the N–H groups by N–D suppresses the formation of **2**, providing direct evidence that hydrogen bonding is essential for its generation. Moreover, the differing interactions (or lack thereof) between various anions and the ligand scaffold (see Response to Reviewer 2, point 2) further demonstrate that hydrogen bonding interactions control the outcome of the reaction of **1** with oxygen. In particular, the triflate anion preorganizes the hydrogen-bonding network in a manner that is crucial for the formation of **2**. Taken together, these observations clearly establish the decisive influence and necessity of hydrogen bonding in the two-electron reduction of dioxygen leading to **2**.

In principle, tuning the hydrogen-bonding strength via electron-withdrawing substituents on the guanidines would be an attractive strategy. In practice, however, the synthetic flexibility of this ligand platform is extremely limited. Although the diisopropylcarbodiimide route to DIG_3tren is straightforward, commercially available carbodiimides with different steric/electronic properties are scarce. We attempted to prepare a tert-butyl analogue using tert-butylcarbodiimide, but its markedly reduced reactivity requires temperatures above 170 °C, leading to extensive side reactions and ultimately insoluble polyguanidines rather than the desired tris-substituted product. The synthesis of carbodiimides themselves is also nontrivial, and our efforts to access methyl- or ethyl-substituted variants have been unsuccessful to date. Consequently, systematic functionalization of the ligand to modulate hydrogen bonding is currently not synthetically feasible within this scaffold.

References

1. Kurtz, D. M. Dioxygen-binding Proteins. in *Comprehensive Coordination Chemistry II* 229–260 (Elsevier, 2003). doi:10.1016/B0-08-043748-6/08171-8.

2. Mot, A. C., Roman, A., Lupan, I., Kurtz, D. M. & Silaghi-Dumitrescu, R. Towards the Development of Hemerythrin-Based Blood Substitutes. *Protein J.* **29**, 387–393 (2010).

3. England, J. *et al.* The crystal structure of a high-Spin oxoiron(IV) complex and characterization of Its self-Decay pathway. *J. Am. Chem. Soc.* **132**, 8635–8644 (2010).

4. Usharani, D., Lacy, D. C., Borovik, A. S. & Shaik, S. Dichotomous Hydrogen Atom Transfer vs Proton-Coupled Electron Transfer During Activation of X–H Bonds (X = C, N, O) by Nonheme Iron–Oxo Complexes of Variable Basicity. *J. Am. Chem. Soc.* **135**, 17090–17104 (2013).

5. Mandal, D. & Shaik, S. Interplay of Tunneling, Two-State Reactivity, and Bell–Evans–Polanyi Effects in C–H Activation by Nonheme Fe(IV)O Oxidants. *J. Am. Chem. Soc.* **138**, 2094–2097 (2016).

6. Gütlich, P., Bill, E. & Trautwein, A. X. *Mössbauer Spectroscopy and Transition Metal Chemistry*. (Springer Berlin Heidelberg, Berlin, Heidelberg, 2011). doi:10.1007/978-3-540-88428-6.

7. Searls, C. E. *et al.* Trigonal bi- and monopyramidal cobalt(II) complexes of a novel guanidine-based tripodal ligand. *Inorg. Chem.* **49**, 11261–11263 (2010).

Reviewer #1 (Remarks to the Author)
The authors have revised the manuscript properly in accordance with comments provided by reviewers. This reviewer would like to ask the authors to consider following three points.

1. The wrong M value of 1 for Hr in the original version confused me to say that it is comparable with the present case; however, if Hr does not form a CO complex, as for CO binding, it is not appropriate to compare the complex reported herein with Hr. To clarify the property of Hr in CO binding, not only in Fig 1(c), but the authors would be better to add "for Hr, M = 0" after "for Hb, M = 243" in the parenthesis on page 2.

As suggested, we have included "for Hr, M = 0" after "for Hb, M = 243" in the parenthesis on page 2. Furthermore, we have modified the abstract and removed the statement saying that **2** is a suitable model complex of Hr.

2. Concerning the calculated structure of the CO complex shown in Suppl. Fig 36(a), although the authors claim that weak hydrogen bonding is operative among the oxygen of CO ligand and the amide N-H protons, this reviewer cannot imagine hydrogen bonding considering the interatomic distances in the figure (2.45, 2.27, 2.74 Å). Does the hydrogen bonding contribute to the comparable stability (M = 0.6) of the Fe(II)-CO complex to the peroxo complex 2?

According to Jeffrey's categorization (Jeffrey, George A.; An introduction to hydrogen bonding, Oxford University Press, 1997. Page 12), the traditional hydrogen bond (HB) by electrostatic interaction can be of significance at distances between 1.6 - 3.1 Å. The HB lengths of 2.37, 2.36, and 2.40 Å, as obtained in our calculation for the S=1 Fe(II)-CO complex is, therefore, within the range of HB distances. However, the strength of this HB is weak, when compared to the iron-peroxo complex, where significantly shorter HB distances @1.74, 1.92, 2.02 Å were obtained. As a response to the comment of the reviewer we have now added a statement on page 6

"Thus, the secondary coordination sphere provided by the DIG_3tren ligand, functions similarly as the distal histidine in Hb/Mb; it provides an ideal environment for strong H-bonds (N–H···O distances of 1.73, 1.95, and 2.00 Å; Fig. 7) with bound oxygen in $[Fe(\eta^1\text{-}O_2)(DIG_3tren)]^{2+}$, but out of place for optimal interaction with a linear bound CO (very weak N–H···O(CO) H-bonding interactions @ 2.37, 2.36, and 2.40 Å; supplementary Fig. 36a for ^{3}**1-CO**) in $[Fe(CO)(DIG_3tren)]^{2+}$. This is believed to be crucial for increasing the dioxygen affinity for $[Fe^{II}DIG_3tren]$ compared to carbon monoxide."

We have also modified the abstract by adding the following sentence

"Mononuclear $[Fe^{II}(DIG_3tren)]^{2+}$ exhibits lower affinity for CO than for O_2, which is attributed to stronger H-bonding stabilization in the $[Fe^{IV}(\eta^1\text{-}O_2)(DIG_3tren)]^{2+}$ complex ."

3. To argue the unusual accessibility to the triplet Fe(IV) state as a peroxo (peroxido) complex, electrochemical data for determining the redox potentials of the Fe(II)-OTf complex (1-OTf) and complex 1 should be reported. The authors need to explain the reason why complex 1 can form a mononuclear trigonal bipyramidal end-on peroxo Fe(IV) complex, since the ligand set is not so electron-donating and the hydrogen bonding formed with the peroxo ligand is expected to reduce its electron-donating ability to stabilize a high-valent state. Although the authors have mentioned to the enhancement of electron acceptability of the superoxo ligand in the Fe(III)-superoxo intermediate by hydrogen bonding with the N-H protons, it should be discussed also based on the redox potentials of Fe(III)/Fe(II) and Fe(IV)/Fe(III) processes of the iron center.

We have now included the electrochemical data in SI Figures 39 and 40. Please note that DIG_3tren ligand is a strongly electron donating ligand, because of the known superbasic character (Sundermeyer et al., 2001) of the guanidine moieties. This is also reflected in the relatively low $Fe^{II/III}$ potential, as determined by cyclic voltammetry (CV) of **1/1-OTf** in CH_3CN. Notably, the value of +0.17 V vs $Fc^{+/o}$ is significantly lower than that recorded for other ligands that are known to stabilize the Fe(IV) oxidation state (see the Table below; *J. Am. Chem. Soc.* **127**, 4178–4179 (2005)). Furthermore, the CV of **3** shows a $Fe^{IV/III}$ reduction wave at -1.63 V vs $Fc^{+/o}$, which is siginificantly cathodically shifted as compared to known Fe^{IV}=O complexes in the literature (*Proc. Natl.*

Acad. Sci. U.S.A. 104 (49) 19181-19186; and *Chem. Sci.*, 2013,**4**, 282-291)

Table S1. $Fe^{III/II}$ Redox Potentials of Nonheme Iron(II) Complexes in Various Solvents[a]

solvent system	$E_{1/2}$ (V)[b,c]		
	$[Fe(TPA)]^{2+}$	$[Fe(N4Py)]^{2+}$	$[Fe(BPMEN)]^{2+}$
CH_3CN	0.36 (r)	0.27 (r)	0.36 (r)
CH_3CN / butyl ether (1:1)	0.38 (r)	0.34 (r)	0.43 (qr)
CH_3CN / THF (1:1)	0.57 (ir)	0.54 (r)	0.68 (ir)

[a] See detailed experimental procedures in Experimental Section of this Supporting Information.

[b] $Fe^{III/II}$ redox potentials are versus Fc^+/Fc.

[c] The r, qr, and ir in parenthesis stand for reversible, quasi-reversible, and irreversible electrochemical processes, respectively, in the given solvent systems.

We have now added statements in the abstract and in the conclusion to address this point of the reviewer.

Abstract: "The O_2^{2-} dianion is stabilized by strong hydrogen bonds to N-H groups from DIG_3tren, which, together with strong electron donation from superbasic DIG moieties reduce the barrier for the unusual Fe(II) mediated two-electron reduction of O_2."

Conclusion: "The facile two-electron electron transfer from Fe^{II} to O_2 is further facilitated by the electron-rich nature of $[Fe(DIG_3tren)]^{2+}$. This is evident from the low $Fe^{III/II}$ potential of +0.17 V vs $Fc^{+/o}$ (determined by cyclic voltammetry; see SI Fig. X), and can be attributed to excellent donor quality of DIG_3tren containing the superbasic guanidine moieties.[103]"

103. Raab, V., Kipke, J., Burghaus, O. & Sundermeyer, J. Copper Complexes of Novel Superbasic Peralkylguanidine Derivatives of Tris(2-aminoethyl)amine as Constraint Geometry Ligands. *Inorg. Chem.* **40**, 6964–6971 (2001).

4. Concerning the Suppl. Table 5, the numbers of complexes should not be 1 but 2.

This has been corrected in Supl. Tables 4 and 5.
